# Electronic Textiles Based on Conductive Metal–Organic Frameworks as Scavengers and Sensors of Toxic Oxyanions from Water

**DOI:** 10.1021/jacs.5c05275

**Published:** 2025-07-28

**Authors:** Patrick Damacet, Priyanshu Chandra, Emma K. Ambrogi, Hyuk-Jun Noh, Ericka L. Asmus, Elissa O. Shehayeb, Giovanni Barcaro, Susanna Monti, Katherine A. Mirica

**Affiliations:** † Department of Chemistry, Burke Laboratory, 3728 Dartmouth College, Hanover, New Hampshire 03755, United States; ‡ CNR-IPCF, Institute for Chemical and Physical Processes, Area della Ricerca, Pisa I-56124, Italy; § CNR-ICCOM, Institute of Chemistry of Organometallic Compounds, Area della Ricerca, Pisa I56124, Italy

## Abstract

Water contamination by toxic oxyanions poses a severe threat to ecosystems and human health. While various adsorbents have been developed for oxyanion sequestration, designing a single material that simultaneously achieves high selectivity, rapid adsorption kinetics, and real-time sensing capabilities remains a challenge. This study explores the first use of layered, conductive metal–organic frameworks (cMOFs) based on hexahydroxy- and hexaimino-triphenylene (HHTP and HITP) cores coordinated with nickel and copper for the dual sensing and filtration of oxyanions from water. Systematic investigations of Ni_3_(HHTP)_2_, Cu_3_(HHTP)_2_, Ni_3_(HITP)_2_, and Cu_3_(HITP)_2_ reveal that Ni_3_(HITP)_2_ exhibits unprecedented adsorption capacities, capturing up to 827 mg of MnO_4_^–^ and 497 mg of Cr_2_O_7_^2–^ per gram of MOF, while filtering up to 99% of these oxyanions within 10 min of exposure. Ni_3_(HITP)_2_ also demonstrates high applicability in real-world scenarios, maintaining a remarkable adsorption performance across various water matrices, pH conditions, and competing anion interferences. Spectroscopic and computational investigations reveal a multimechanistic scavenging process involving chemisorption, physisorption, and redox reactions. Grafting Ni_3_(HITP)_2_ onto cotton textiles via a layer-by-layer approach yields mechanically robust, easy to handle, and flexible electronic textile capable of filtering oxyanions for up to 32 cycles without performance loss, while allowing their detection with high sensitivity and low detection limits reaching 2.2 ppm for MnO_4_^–^ and 6 ppm for Cr_2_O_7_^2–^. Taken together, these findings pave the way for MOF-based next-generation water treatment technologies that integrate efficient filtration and real-time sensing capabilities.

## Graphical abstract

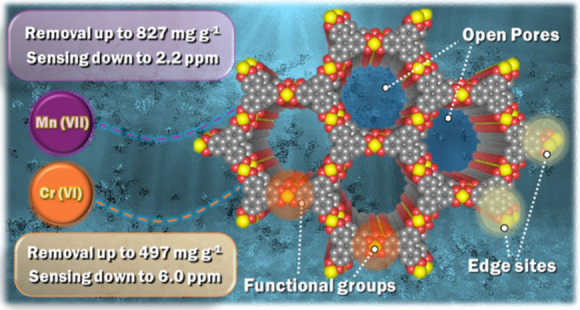

## 1 Introduction

The rapid proliferation of toxic metal oxyanions in water bodies, spurred by the exponential growth in industrialization and urbanization has recently surfaced as a critical environmental concern that imperils both, ecosystems and human health alike.^1–3^ Among these oxyanions, dichromate (Cr_2_O_7_^2–^) and permanganate (MnO_4_^–^) are highly worrisome due to their acute mutagenic, cytotoxic, and carcinogenic effects, earning them recognition as hazardous substances under federal environmental guidelines.^4–7^ The high discharge rate of these pollutants into natural water sources, driven by their widespread use in steel manufacturing, leather tanning, wood preservation, textile dyeing, and metal finishing, has created an urgent need for novel remediation technologies.^8–10^ While a variety of techniques have been employed for scavenging oxyanions from water, including electrocatalytic reduction,^11,12^ chemical precipitation,^13,14^ photodecomposition,^15^ biological treatment,^16,17^ and membrane filtration,^18,19^ adsorptive decontamination remains one of the most promising and versatile approaches due to its high efficiency, cost-effectiveness, minimal waste generation, and ease of operation.^20–22^ To date, several adsorbents based on layered double hydroxides (LDHs),^23,24^ polyaniline-loaded materials,^25,26^ carbonaceous materials,^27,28^ and metal oxides^29,30^ have been explored for oxyanion removal from water. However, their low specificity, limited reusability, suboptimal adsorption efficiency, and slow adsorption kinetics continue to pose significant challenges, restricting their practical implementation in real-world applications.^31–33^ Moreover, most of these adsorbents are intrinsically monofunctional and therefore, lack the capability to simultaneously detect and remove oxyanion contaminants from water. This functional limitation hinders their practical utility, as oxyanions must be monitored at low parts-per-million (ppm) or even parts-per-billion (ppb) concentrations to meet environmental safety standards.^7,34^ Consequently, there is a growing need for dual-function materials that can not only capture oxyanions efficiently, but also monitor their concentrations in real time, providing both remediation and early warning capabilities in contaminated water sources.^20^

Metal–organic frameworks (MOFs) represent a promising class of porous, crystalline materials with significant potential for removing toxic pollutants from water.^35,36^ Their permanent porosities, tunable functionalities, and high surface areas have made them highly effective adsorbents for a diverse range of contaminants, including heavy metals,^37^ pesticides,^38^ nanoplastics,^39^ pharmaceuticals,^40^ organic dyes,^41^ metal-derived oxyanions,^20^ and radioactive nuclear waste.^42^ Despite their advantages, traditional MOFs face four key limitations that hinder their widespread use. First, many reported MOFs suffer from metal ion and/or ligand leaching into solution during remediation, which can reduce their effectiveness in oxyanion scavenging and introduce additional water contamination.^43,44^ Second, the high hydration energy, relatively large size, and high charge density of most oxyanions make their adsorption by conventional 3D and charge neutral MOFs challenging.^45–47^ Third, the intrinsic electrical insulating nature and redox inactivity of most MOFs used in water filtration limits their multifunctional capabilities, preventing simultaneous sensing, filtration, and detoxification of toxic pollutants.^48^ Fourth, MOF powders are often difficult to handle due to their small particle size and tendency to agglomerate in solution, complicating their practical use in filtration systems. Integrating MOFs within textile fabrics offers a promising strategy to improve their handling and deployment in water filtration systems.^49^ However, achieving uniform and stable MOF deposition on textiles remains a significant challenge with the MOFs currently employed for water remediation.^50–54^ Building upon these limitations, two-dimensional conductive MOFs (2D cMOFs) offer promising solutions due to their (i) multifunctional nature,^55^ (ii) ability to be deposited onto textiles,^56,57^ (iii) relative stability in aqueous media,^58^ (iv) abundance of open metal sites and edge sites that act as Lewis acid–base sites,^59,60^ (v) surface charge, and (vi) redox-activity, thus allowing for contaminant filtration through a synergistic mechanism involving chemisorption, physisorption, and redox reactions. Consequently, 2D cMOFs are expected to exhibit superior performance in oxyanion scavenging, overcoming the key challenges faced by conventional MOF adsorbents.

Herein, we present the first use of layered 2D cMOFs for the simultaneous capture, detection, and detoxification of two model oxyanions, Cr_2_O_7_^2–^ and MnO_4_^–^, from water. The as-synthesized MOFs, based on hexahydroxy- and hexaimino-triphenylene (HHTP and HITP) cores coordinated with nickel and copper metal ions exhibit exceptional performance in oxyanion removal, excelling in both uptake capacity and adsorption kinetics. Concentration- and time-dependent adsorption studies demonstrate experimental uptakes of up to 827  ±  79 mg of MnO_4_^–^ and 497  ±  11 mg of Cr_2_O_7_^2–^ per gram of MOF, with a 99% removal efficiency achieved for 25 ppm contaminant solutions in under 10 minutes of contact. The best performing MOF, Ni_3_(HITP)_2_, is found to display adsorption capabilities across a broad pH range (4–10), high selectivity in the presence of competing anions, and consistent performance across diverse water matrices, making it highly suitable for real-world applications. Spectroscopic investigations at the molecular-level, coupled with Quantum Chemistry (QC) simulations using Density Functional Theory (DFT) reveal a synergistic scavenging mechanism involving chemisorption, physisorption, and redox reactions. To enhance practical handling, recovery, and deployment in water filtration systems, while simultaneously enabling their use as electrochemical sensors, we grafted Ni_3_(HITP)_2_ onto cotton fabrics using a layer-by-layer (LbL) approach. The resulting MOF@textile composite maintains an adsorption capacity comparable to the bulk material, exhibits high durability, and can be regenerated for up to 32 cycles without noticeable performance loss, while enabling oxyanion detection in the low ppm range. By combining high-efficiency adsorption with real-time sensing, this work not only introduces a new class of MOF-based adsorbents for oxyanion remediation but also establishes a foundation for the development of textile-integrated materials in next-generation water treatment nanotechnologies.

## 2 Experimental Design

### Choice of Metal-Derived Oxyanions

We selected MnO_4_^–^ and Cr_2_O_7_^2–^ as representative model oxyanions for our dual adsorption and sensing studies for five major reasons. First, Cr_2_O_7_^2–^ is regarded as one of the most hazardous oxyanions, due to its mutagenic and carcinogenic effects on living organisms.^4^ It is known to cause DNA damage, chronic respiratory diseases, and extensive cell and tissue damage,^61^ and has consequently been classified as a Group 1 carcinogen by the International Agency for Research on Cancer (IARC).^62^ In contrast, while MnO_4_^–^ is generally considered less toxic than Cr_2_O_7_^2–^, it remains a potent neurotoxin, capable of causing Parkinsonism, tissue damage, and gastrointestinal distress in humans when present at relatively high concentrations.^7,63^ Second, incidents of drinking water contamination involving manganese and chromium ions have been widely reported across various countries,^3,64,65^ underscoring the urgent need for the development of cost-effective and efficient water remediation technologies. Third, the United States Environmental Protection Agency (USEPA) has established guideline values of 50 ppb for chromium ions and 300 ppb for manganese ions in drinking water, emphasizing the ongoing need to (i) monitor and (ii) limit the concentrations of these contaminants to protect public health.^3,66^ Fourth, concentrations of these oxyanions in industrial wastewater have been reported to reach as high as 270 ppm, necessitating the development of high-efficiency adsorbent materials capable of reducing Mn­(VII) and Cr­(VI) levels well below the discharge standards set by environmental agencies for safe release into aquatic environment.^67–69^ Well-documented cases of water contamination by these oxyanions include the Hinkley groundwater incident, as well as widespread manganese contamination in groundwater and drinking water sources in the United States and Bangladesh.^70–73^ Fifth, MnO_4_^–^ and Cr_2_O_7_^2–^ differ in their hydration energies, molecular sizes, and charge densities. Understanding the interaction mechanisms between 2D cMOFs and these oxyanions through structure–property interconnections is expected to guide the future design of hierarchical adsorbent materials with tailored properties for efficient oxyanion removal from water.

### Choice of MOF Materials

We selected four representative 2D cMOFs based on HHTP and HITP cores with copper and nickel metal nodes for the simultaneous sensing and filtration of MnO_4_^–^ and Cr_2_O_7_^2–^ for six major considerations (Figure 1). First, these MOFs possess a unique combination of structural features, including (i) unsaturated open metal sites, (ii) vertically aligned porous channels, (iii) aqua-rich capping edge sites, and (iv) high chemical stability in aqueous solutions.^59,60,74^ These properties endow 2D cMOFs highly effective for ion adsorption under various conditions. Second, the redox-active nature of 2D cMOFs allows not only for effective adsorption but also for the detoxification of oxyanions in water. In particular, the reduction of MnO_4_^–^ can result in the formation of the less toxic MnO_2_, which is known to have adsorptive remediation properties.^75,76^ Likewise, the reduction of Cr_2_O_7_^2–^ to Cr­(III) species can substantially lower chromium toxicity in aqueous media, as Cr­(III) is significantly less harmful than Cr­(VI), and is even used in trace amounts as a nutritional and dietary supplement.^77^ Third, both experimental and computational studies have shown that HHTP- and HITP-based MOFs exhibit a measurable surface charge in water, which may promote electrostatic interactions with the charged oxyanions.^78,79^ Fourth, the intrinsic electrical conductivity equips these materials with multifunctional capabilities, allowing their usage, besides adsorbents, as electrochemical sensors for monitoring oxyanion concentrations in water. Fifth, the unique structural features of this class of 2D cMOFs provide valuable insights into the structure–function relationships that govern oxyanion adsorption, particularly in terms of the roles of metal nodes and functional groups. Sixth, the synthetic precursors and preparation methods for these MOFs are readily accessible and well-established,^80,81^ offering a cost-effective and straightforward route for fabricating high-performance adsorbents and sensors for water filtration applications.

**Figure 1:**
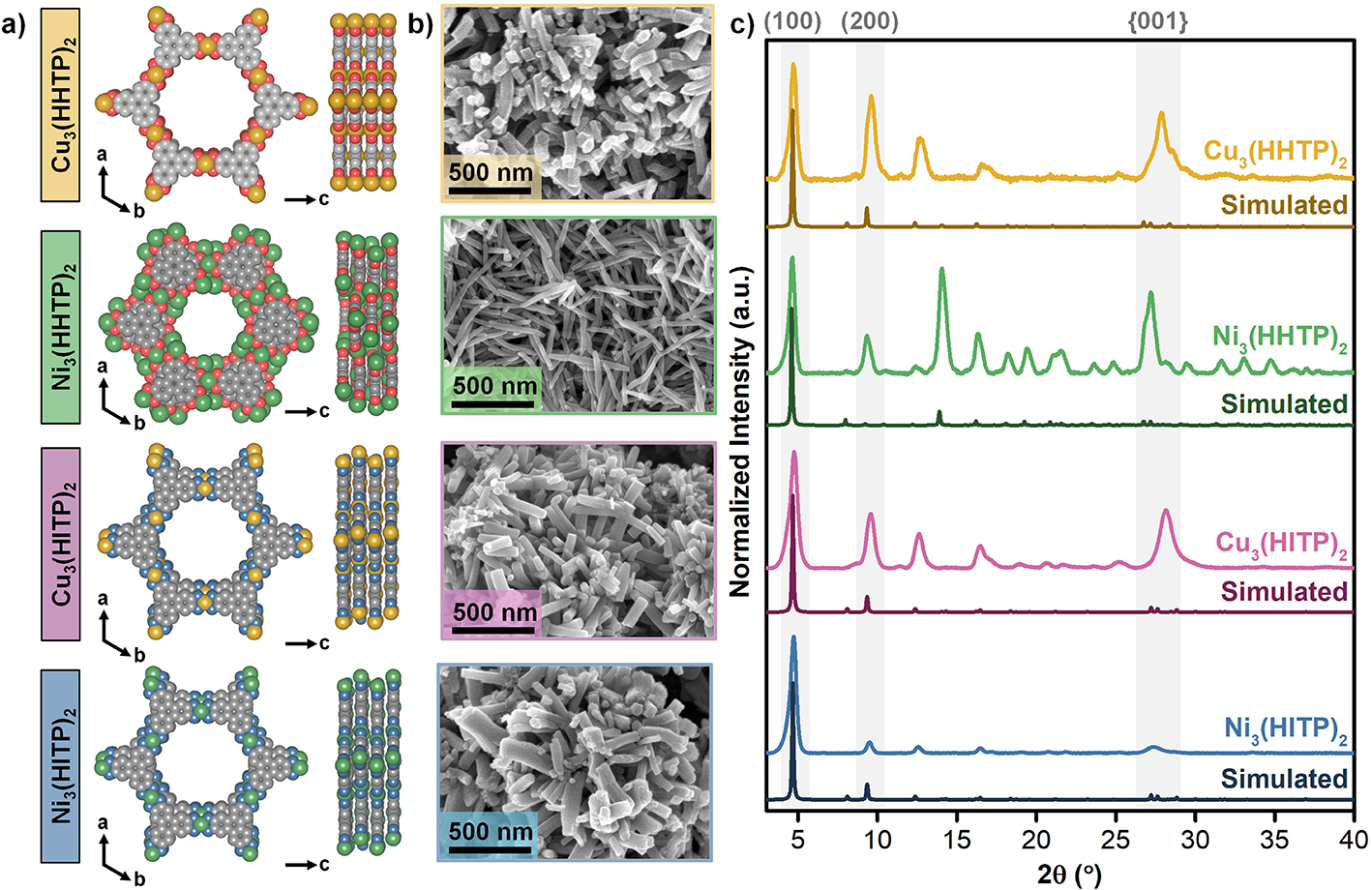
a) Simulated crystal structures b) SEM micrographs, and c) PXRD patterns of the series of HHTP- and HITP-based 2D cMOFs employed in this work.

## 3 Results and Discussion

### Characterization of MOF Materials

We employed well-established solvothermal procedures to generate microcrystalline powders of four layered cMOFs: Cu_3_(HHTP)_2_ and Ni_3_(HHTP)_2_, based on HHTP cores, and Cu_3_(HITP)_2_ and Ni_3_(HITP)_2_, derived from HITP cores (see Section S2). In addition to differences in metal nodes (Cu^2+^ vs Ni^2+^) and functional groups (−OH vs -NH_2_), these MOFs exhibit distinct stacking arrangements. While Cu_3_(HITP)_2_ and Ni_3_(HITP)_2_ adopt a slipped-parallel stacking configuration,^81^ their HHTP-based counterparts display unique patterns. Specifically, Ni_3_(HHTP)_2_ forms a bilayered structure wherein extended two-dimensional sheets alternate with intercalated layers of nickel catecholate complexes, whereas Cu_3_(HHTP)_2_ adopts a near-eclipsed, C-centered monoclinic crystal structure (Figure 1a).^80,82^ Prior to conducting the adsorption experiments, we characterized the house-made HATP·6HCl (Triphenylene-2,3,6,7,10,11-hexaamine hexahydrochloride) molecular precursor and the MOF adsorbents to confirm their purity and structural integrity. Nuclear magnetic resonance (NMR) and mass spectrometry (MS) analyses verified the successful synthesis and high purity of HATP·6HCl (Figures S1–S3). Powder X-ray diffraction (PXRD) measurements revealed the formation of highly crystalline MOF particles, with sharp, intense peaks that closely matched the simulated PXRD patterns of the corresponding crystal structures, suggesting the high phase purity of the resulting materials (Figures 1a,c and S4–S7). Scanning electron microscopy (SEM), in conjunction with energy-dispersive X-ray spectroscopy (EDX), revealed that the MOFs exhibit well-defined rod-like morphologies, with uniform elemental distributions throughout the crystals (Figures 1b and S8–S11). Importantly, these structural and morphological features remained consistent across two independent synthesis batches, confirming the reproducibility and reliability of the synthetic procedure in producing phase-pure 2D cMOF crystals (Figures S12–S17). Additionally, the activated MOFs exhibited satisfactory thermal stabilities of up to 200 °C as indicated by thermogravimetric analysis (TGA), highlighting their potential for use under various operational conditions (Figure S18). Detailed synthetic procedures and characterization features for the MOF series are provided in Section S2 of the Supporting Information.

### Removal of Oxyanions from Water Using 2D cMOFs

We began our investigations by evaluating the uptake capacities of the four triphenylene-based MOFs toward two model oxyanions through batch adsorption experiments. We varied the concentrations of MnO_4_^–^ and Cr_2_O_7_^2–^ between 5 and 500 ppm, and estimated the equilibrium uptake capacities (Q_e_) of the MOFs following 24 h of adsorption using inductively coupled plasma mass spectrometry (ICP-MS) via Equation S1. As shown in Figure 2a, all MOFs exhibited high and comparable adsorption capacities for MnO_4_^–^ across the tested concentration range, as evidenced by the sharp and linear increase in Q_e_ with the initial MnO_4_^–^ concentration. Notably, we found the MOFs to filter up to 94 ± 2% of MnO_4_^–^ from water at an initial concentration of 500 ppm, suggesting highly favorable interactions between the frameworks and Mn­(VII) (Figure S19). Estimating the experimental maximum uptake capacity (*Q*_max_) of these MOFs via saturation adsorption experiments revealed that Ni_3_(HITP)_2_ exhibits the highest uptake performance, reaching a *Q*_max_ up to 827 ± 79 mg of MnO_4_^–^ per gram of MOF within 9 h (Figures S19–S20). To the best of our knowledge, this performance rivals that of the most efficient MOF-based adsorbents reported to date for MnO_4_^–^ (Figure S21).

**Figure 2:**
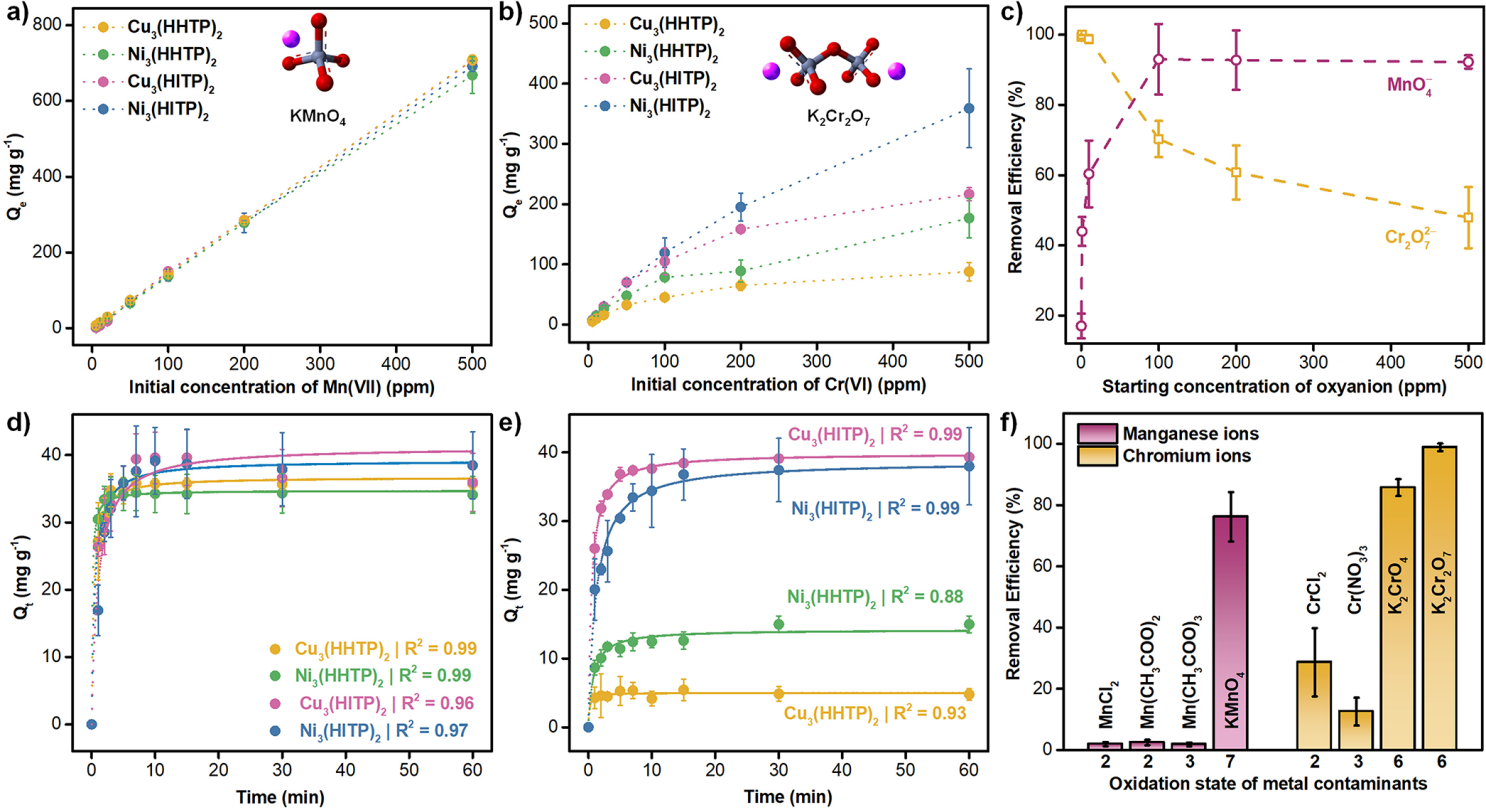
Concentration-dependent adsorption uptakes of MOFs toward a) MnO_4_^–^ and b) Cr_2_O_7_^2–^ oxyanions. c) Removal efficiency of Ni_3_(HITP)_2_ toward different concentrations of MnO_4_^–^ and Cr_2_O_7_^2–^. Time-dependent adsorption uptakes of MOFs toward d) MnO_4_^–^ and e) Cr_2_O_7_^2–^ oxyanions. f) Selectivity of Ni_3_(HITP)_2_ to manganese and chromium species (20 ppm) with different charges and oxidation states. Error bars represent standard deviation from the mean value of three independent experiments. Conditions: m_MOF_ = 2 mg, V_Solution_ = 3 mL, and *T* = 298 K. Note the initial concentration of the oxyanions in the kinetic experiments is 25 ppm.

Turning our attention toward Cr_2_O_7_^2–^, we noted Q_e_ for all MOFs to exhibit an increase with rising initial oxyanion concentrations before approaching saturation at high-ppm concentrations, where the adsorptive sites likely became fully occupied (Figure 2b). Notably, HITP-based counterparts demonstrated a superior adsorption performance compared to their HHTP-based MOFs, reaching Q_e_ values of 195 and 158 mg g^–1^ and Cu_3_(HITP)_2_, respectively, compared to 89 and 65 mg g^–1^ for their HHTP-based counterparts at an initial Cr_2_O_7_^2–^ concentration of 200 ppm (Figure S22). Saturation adsorption experiments further revealed that Ni_3_(HITP)_2_ reached a *Q*_max_ of 497 ± 11 mg g^–1^ for Ni_3_(HITP)_2_ in 9 h of contact, more than twice that of Cu_3_(HHTP)_2_ and up to 3.7 times higher than the HHTP-based analogs (Figure S22–S23). To our knowledge, this *Q*_max_ value for Ni_3_(HITP)_2_ exceeds those of most MOF-based adsorbents reported to date (Figure S24). To elucidate the surface charge properties of these MOFs and rationalize the superior *Q*_max_ values observed for the HITP-based MOFs in oxyanion adsorption, we carried out both, zeta potential measurements, and dye adsorption experiments using cationic methylene blue (MB^+^) and anionic methyl orange (MO^–^). We found the HHTP-based MOFs to display a consistently negative surface charge under ambient aqueous conditions, as evidenced by their (i) negative zeta potential values ranging from −22.5 mV to −38.6 mV, and (ii) complete adsorption of MB^+^ alongside negligible uptake of MO^–^ after 2 h of contact (Figures S25–S26 and S29–S30). In contrast, HITP-based MOFs exhibited amphoteric surface properties, with a net positive charge in water, as evidenced by their (i) complete removal of MO^–^, (ii) partial adsorption of MB^+^, and (ii) positive zeta potential values of +21.4 mV and +12.7 mV for Cu_3_(HITP)_2_ and Ni_3_(HITP)_2_, respectively (Figures S27–S28 and S31–S33). These findings underscore the role of electrostatic interactions between the MOF adsorbents and oxyanions in governing the removal capacity of the negatively charged oxyanions in water.

Despite the higher *Q*_max_ values of Ni_3_(HITP)_2_ for MnO_4_^–^ compared to Cr_2_O_7_^2–^, we found that the MOF exhibited a remarkably higher affinity toward Cr_2_O_7_^2–^ at low ppm concentrations (0.1–10 ppm). As shown in Figure 2c, the removal efficiency of Ni_3_(HITP)_2_ for Cr_2_O_7_^2–^ decreased from 100% to 47% as the contaminant concentration increased from 0.1 to 500 ppm, which aligns with the expected saturation of active adsorption sites at higher relative concentrations. In contrast, we observed the opposite trend for MnO_4_^–^, where removal efficiency increased dramatically from 17% to 93% over the same concentration range, challenging conventional expectations.^83,84^ We hypothesized that at low oxyanion concentrations (0.1–10 ppm), the limited concentration gradient of contaminants restricted both, their (i) availability near adsorptive active sites and (ii) their mass transport rate into the pores of Ni_3_(HITP)_2_, resulting in oxyanion-MOF interactions predominantly occurring at the MOF surface rather than within its internal pores.^85,86^ Given that Cr_2_O_7_^2–^ has higher polarizability, multidentate potential, and a greater number of nucleophilic oxygen atoms compared to MnO_4_^–^, we expect that it exhibited a (i) stronger physical interactions through hydrogen bonding with electrophilic hydrogen donor groups in HITP moieties and (ii) greater electrostatic attraction to charged open metal sites on the MOF surface. On the contrary, increasing the concentration of oxyanions will result in an increase in the adsorption mass transfer rate, which is expected to facilitate the diffusion of oxyanions onto the pores of the MOFs.^87^ Given that MnO_4_^–^ has a smaller radius in its hydrated form compared to Cr_2_O_7_^2–^, it is expected to access the pores of Ni_3_(HITP)_2_ more readily at higher concentrations.^88,89^

We further examined the adsorption kinetics of the frameworks by exposing them to 25 ppm solutions of MnO_4_^–^ and Cr_2_O_7_^2–^ at different time intervals. We reasoned that these experiments would provide insight into the adsorption rate and efficiency of these materials in real-world water remediation applications, where oxyanions commonly exist in similar ppm-level concentrations in industrial and polluted water sources.^90,91^ For all MOF-oxyanion pairs, we observed a rapid and steady increase in the time-dependent adsorption capacity (*Q*_t_) with adsorption time, followed by a plateau after 7 min, suggesting the saturation of the MOF adsorption sites (Figure 2d-e). While all studied MOFs displayed comparable adsorption rates for MnO_4_^–^, capturing between 92 and 99% of the oxyanion within 7 min, HITP-based MOFs outperformed their HHTP-counterparts in adsorbing Cr_2_O_7_^2–^. This trend in oxyanion uptake aligned well with our concentration-dependent adsorption studies, where HITP-based MOFs, characterized by (i) a positive surface charge and (ii) an abundance of electrophilic hydrogen donors from their nickel­(bisdiimine) linkages demonstrated superior affinity toward Cr_2_O_7_^2–^.

To elucidate the mode of interactions of the MOFs toward the oxyanions, we proceeded by fitting the kinetic uptake data into the linear forms of the pseudo-first-order and pseudo-second-order kinetic models, as described in Section S3.4 of the SI. We found the experimental adsorption data for all MOF-oxyanion pairs to align more closely with the pseudo-second-order kinetic model, as indicated by the higher correlation coefficients (0.94–0.99) obtained from their least-squares regression, compared to the pseudo-first-order model (0.42–0.67). These findings suggested that the adsorption process is predominantly chemisorption-driven, where covalent binding of MnO_4_^–^ and Cr_2_O_7_^2–^ to MOF active sites governs the overall adsorption rate (Figures S34–S43). Further examination of the kinetic data using the Weber–Morris intraparticle diffusion model indicated a multistage diffusion process governing the adsorption of oxyanions onto the MOFs, evidenced by the predominant triphasic nature of the plots obtained.^92,93^ These diffusion processes involved (i) a rapid external surface adsorption in the initial stage, followed by (ii) gradual diffusion of oxyanions into the MOF pores, where chemical interactions with active sites occur, and last (iii) a saturation phase, where most adsorption sites become occupied, limiting further uptake (Figures S38 and S43).

### Oxyanion Removal under Diverse Aquatic Environments

An essential requirement for an effective adsorbent is its ability to maintain a high performance in the presence of various environmental interferences. To assess the practical applicability of Ni_3_(HITP)_2_, the best-performing MOF in this study, we examined its removal efficiency under different aquatic conditions. We first spiked real water samples, including tap water (New Hampshire), river water (Connecticut river), and seawater (Atlantic Ocean) with 100 ppm solutions of MnO_4_^–^ and Cr_2_O_7_^2–^ and assessed their removal following 24 h of adsorption using ICP-MS. As shown in Figure 3a, Ni_3_(HITP)_2_ exhibited remarkably high removal efficiencies for both oxyanions, exceeding 89% in both, tap and river water, relative to its performance in DI water. In seawater, nonetheless, we found the removal efficiency to decrease to 82% for MnO_4_^–^ and 31% for Cr_2_O_7_^2–^, which we attributed to the high salinity of the matrix, exceeding 34 practical salinity unit (psu).^94^ Expanding our investigation to the impact of coexisting ions, we spiked 25 ppm oxyanion solutions with equimolar concentrations of six potassium salts containing chloride, nitrate, carbonate, phosphate, acetate, and sulfate counterions, which were chosen to represent a range of ionic sizes and charge densities. ICP-MS analysis of the supernatants following 24 h of adsorption revealed a relatively high retention of adsorption performance, with Ni_3_(HITP)_2_ maintaining 96% uptake of Cr_2_O_7_^2–^ and 75% uptake of MnO_4_^–^ in the presence of competing anions (Figure 3b). These results indicated that, despite the presence of potentially interfering species, Ni_3_(HITP)_2_ effectively removed the target oxyanions, highlighting its robust adsorption capabilities.

**Figure 3:**
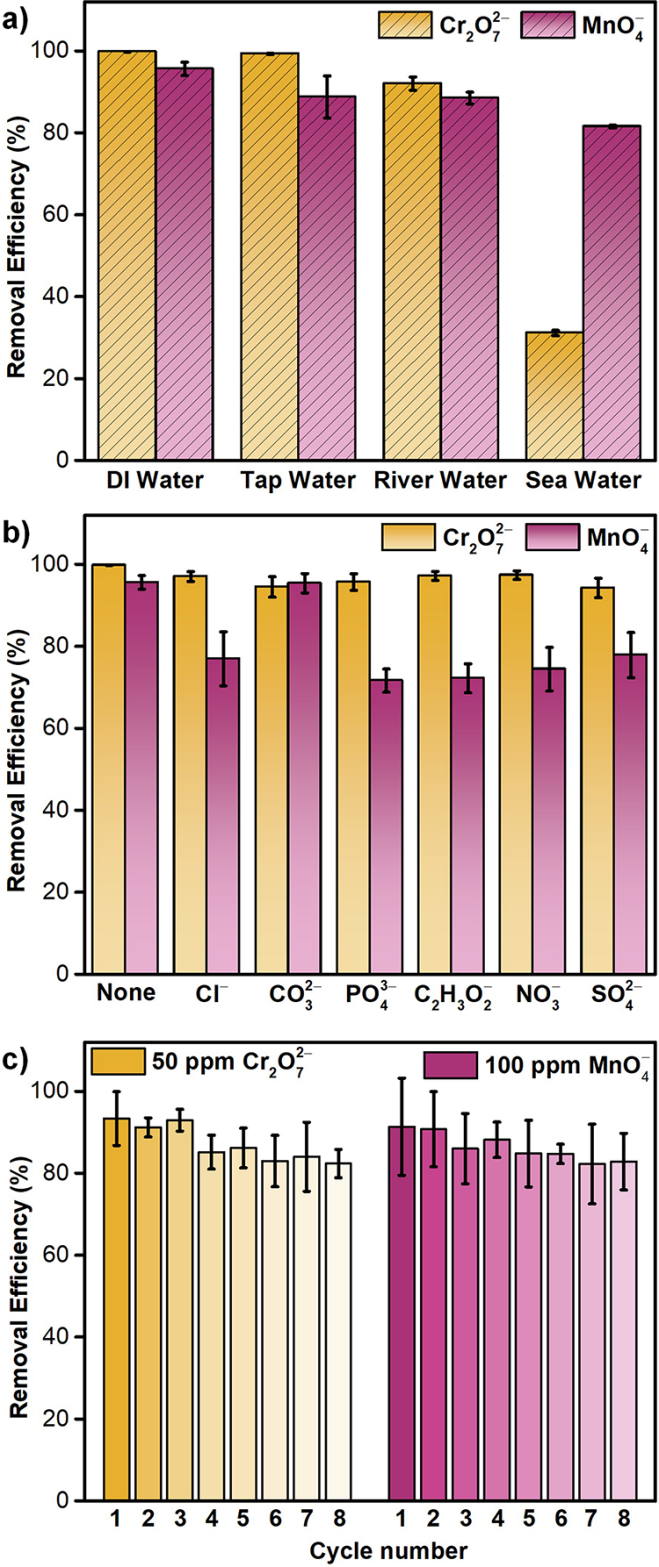
Removal efficiency of Ni_3_(HITP)_2_ toward MnO_4_^–^ and Cr_2_O_7_^2–^ oxyanions in a) different water sources, b) the presence of 25 ppm of coexisting ions and c) cyclic adsorption–desorption experiments.

Examining the removal capabilities of Ni_3_(HITP)_2_ across pH levels ranging from 4 to 10, revealed complete filtration (>99%) of MnO_4_^–^ under all tested conditions. In contrast, Cr_2_O_7_^2–^ removal experienced a gradual decline, decreasing from 68% under acidic conditions (pH 4) to 25% at pH 10, while the MOF maintained high crystallinity throughout the tested pH range (Figures S44–S45). We attributed this trend to the oxyanion speciation under varying pH levels in aqueous solutions.^95^ While Cr_2_O_7_^2–^ and hydrogen chromate (HCrO_4_^–^) are the predominant Cr­(VI) species in acidic and neutral media, chromate (Cr_2_O_4_^2–^) becomes predominant under alkaline conditions.^31,96^ We hypothesized that this change in size, geometry, and number of nucleophilic oxygen atoms of Cr­(VI) likely reduced its affinity towards the MOF in basic environment. In contrast, the complete removal of MnO_4_^–^ can be attributed to its stability in the tested pH range. Although MnO_4_^–^ can disproportionate to form hypomanganate (Mn­(V)) under strongly alkaline conditions, and decompose under strong acidic conditions to form manganese dioxide (Mn­(IV)), these transformations likely did not occur within the examined pH range.^97^ To further evaluate the selectivity of Ni_3_(HITP)_2_ toward these oxyanions, we carried out adsorption experiments for manganese and chromium species with varying charges and oxidation states. Our results showed (i) a significantly higher affinity of Ni_3_(HITP)_2_ for Cr_2_O_7_^2–^ compared to CrO_4_^2–^ (80% vs 21% removal at 100 ppm), confirming the influence of Cr­(VI) speciation, and (ii) a strong preference for negatively charged oxyanions over metal cations (Mn^2+^, Mn^3+^, Cr^2+^, and Cr^3+^), reinforcing the role of electrostatic interactions and hydrogen-bonding in the adsorption mechanism (Figures 2f and S46). Next, we assessed the regeneration capability of Ni_3_(HITP)_2_ through cyclic adsorption test. Remarkably, the removal efficiencies for both, MnO_4_^–^ and Cr_2_O_7_^2–^, remained consistently high (>83%) even after eight consecutive adsorption–desorption cycles (Figures 3c and S47). Moreover, PXRD analysis of the regenerated MOF confirmed its structural integrity following desorption with 1 M HCl for 24 h, highlighting its promising reusability for oxyanion removal (Figure S48).

### Spectroscopic Assessment of MOF–Ion Interaction

To elucidate the interaction mechanism between the oxyanions and MOF adsorbents at the molecular level, we employed a suite of spectroscopic techniques to characterize the MOFs after exposure to MnO_4_^–^ and Cr_2_O_7_^2–^. Attenuated Total Reflectance Fourier-Transform Infrared (ATR-IR) spectra revealed a blue shift in the C–N stretching peaks of HITP-based MOFs following oxyanion adsorption. Specifically, we noted shifts from 1304 cm^–1^ to 1315 cm^–1^ and 1318 cm^–1^ in Ni_3_(HITP)_2_ and from 1292 cm^–1^ to 1318 cm^–1^ and 1316 cm^–1^ in Cu_3_(HITP)_2_ after adsorption of Cr_2_O_7_^2–^ and MnO_4_^–^, respectively. In contrast, the C–O stretching peaks of HHTP-based MOFs remained unchanged after adsorption (Figures 4a and S49). These findings suggested an increase in electron density within the C–N bonds, which we attributed to physisorptive interactions between the electronegative oxygen atoms of the oxyanions and the hydrogen atoms of the bis­(diimine) moieties, a feature absent in HHTP-based MOFs.^98,99^ SEM micrographs confirmed that Ni_3_(HITP)_2_ crystals retained their morphology after adsorption, while energy-dispersive X-ray spectroscopy (EDX) verified the successful uptake of oxyanions by the MOF crystals (Figures S50–S57). The corresponding elemental analysis further revealed a correlation between the initial oxyanion concentration in solution and their corresponding weight percentages detected on Ni_3_(HITP)_2_. Specifically, as the MnO_4_^–^ concentration increased from 50 to 500 ppm, the manganese content (in terms of weight %) in Ni_3_(HITP)_2_ rose from 7.5% to 46%. Similarly, the chromium content increased from 6.7% to 17% over the same concentration range (Figures S58–S59), confirming the concentration-dependent adsorption process of Ni_3_(HITP)_2_.

**Figure 4:**
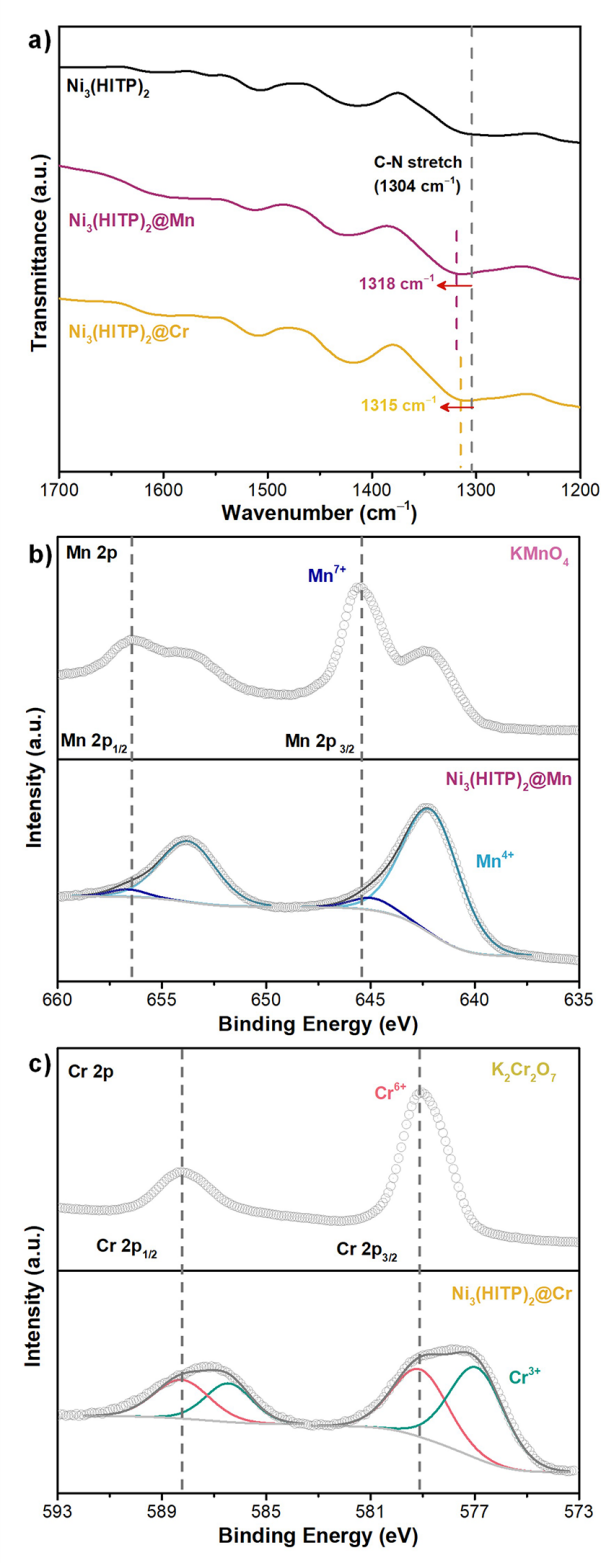
Mechanistic insights into the interaction between Ni_3_(HITP)_2_ and the oxyanions. (a) ATR-IR spectra of bulk Ni_3_(HITP)_2_ MOF before (black), and after (yellow and purple) adsorption of MnO_4_^–^ and Cr_2_O_7_^2–^. (b) High-resolution Mn 2p XPS spectra of KMnO_4_ and Ni_3_(HITP)_2_@Mn. (c) High-resolution Cr 2p XPS spectra of K_2_Cr_2_O_7_ and Ni_3_(HITP)_2_@Cr.

X-ray photoelectron spectroscopy (XPS) measurements provided evidence of chemisorptive interactions between Ni_3_(HITP)_2_ and the oxyanions, supporting the adsorption mechanism proposed by the kinetic models. First, XPS survey spectra of Ni_3_(HITP)_2_ after adsorption confirmed both, the structural integrity of the MOF, and the successful adsorption of oxyanions, as indicated by the appearance of Mn and Cr binding energy peaks (Figure S60a). High-resolution Mn 2p, Cr 2p, and N 1s spectra, depicted in Figures 4 and S61, revealed that redox reactions occurred during the adsorption process. Specifically, MnO_4_^–^, initially present as Mn­(VII) underwent partial reduction to Mn­(IV), evidenced by the emergence of binding energy peaks at 642.2 and 645.0 eV, corresponding to Mn­(IV) and Mn­(VII), respectively (Figure 4b).^100^ We further confirmed the formation of Mn­(IV) through PXRD measurements and SEM imaging, which revealed the formation of amorphous MnO_2_ nanoparticles with a sheet-like morphology after adsorption (Figures S62–S63).^101,102^ Photographs of the solutions before and after saturation adsorption experiments, shown in Figure S64, revealed a significant change in color of solutions from pink, the original color of MnO_4_^–^, to red, which we attributed to the emergence of Mn­(IV) species such as MnO_2_.^103^ Similarly, Cr_2_O_7_^2–^, initially present as Cr­(VI), underwent partial reduction during adsorption, as indicated by the appearance of a new peak at 577.1 eV in the high resolution Cr 2p spectrum, assigned to Cr­(III) (Figure 4c).^104,105^ These transformations in oxyanions coincided with the partial oxidation of the HITP ligand. High-resolution N 1s spectra showed an increase in the ratio of quinoid imine (C=N) to benzenoid amine (C–NH) from 43%/57% before adsorption to 50%/50% and 52%/48%, after MnO_4_^–^ and Cr_2_O_7_^2–^ capture, respectively (Figure S48). Meanwhile, Ni 2p spectra displayed no changes in oxidation state, suggesting that the metal nodes did not participate in the redox process (Figure 60b). We attributed the greater reduction of Mn­(VII) to Mn­(IV) compared to Cr­(VI) to Cr­(III) to the higher standard redox potential of MnO_4_^–^ (1.68 vs 1.33 eV), making the latter a stronger oxidizer and thus driving a more pronounced redox reaction and higher adsorption uptake.^106–108^ Indeed, competitive adsorption experiments using mixtures of MnO_4_^–^ and Cr_2_O_7_^2–^ at varying initial concentrations consistently showed a preferential uptake of MnO_4_^–^ over Cr_2_O_7_^2–^, further supporting this trend (Figures S65–S67). Notably, the redox-active Ni_3_(HITP)_2_ not only captured hazardous oxyanions from water but also detoxified them by reducing Mn­(VII) to Mn­(IV) and Cr­(VI) to Cr­(III) species, that are significantly less toxic and mobile in water. By mimicking the natural detoxification pathways of certain bacteria and fungi, which enzymatically reduce metal-derived oxyanions to less harmful species,^109,110^ Ni_3_(HITP)_2_ demonstrates dual functionality that positions it as a promising candidate for real-world water remediation applications.

### Computational Modeling

The modeling activity was focused on the most performant MOF, i.e. Ni_3_(HITP)_2_, to disclose the atomistic origin of its capture properties. We employed QC simulations at the Density Functional level of theory to estimate the interaction energy of Cr_2_O_7_^2–^ and MnO_4_^–^ oxyanions with the framework at low concentrations of the adsorbates. We considered both the slipped-parallel (SP) arrangement shown in Figure 1a and the structure shown in Figure 5, in which the individual layers are organized in stacking sequences analogous to those of fcc crystals (ABC type). This latter configuration was identified by RMD (Reactive Molecular Dynamics) in a previous investigation of the Cu_3_(HHTP)_2_ system.^56^ QC calculations of a reduced 4L-ABCB (1 × 1) unit cell demonstrated, as for the Cu-based systems, a close energy competition between the two types of morphologies. The peculiar characteristics of this new arrangement are the presence of open-metal sites (OMS), environmentally exposed regions (highlighted in red circles in Figures 5 and S68), and undulated layers, which more favorably interact with the environment. The results of the calculations are displayed in Figure 5, which shows the optimized structures with a quantitative analysis of the adhesion energies. Due to periodic boundary conditions, we had to model the oxyanions as neutral species (namely, the respective acids). Still, we verified that, for finite-size systems where a charge could be included (see Figure S69), the trend of interaction energy difference was comparable.

**Figure 5:**
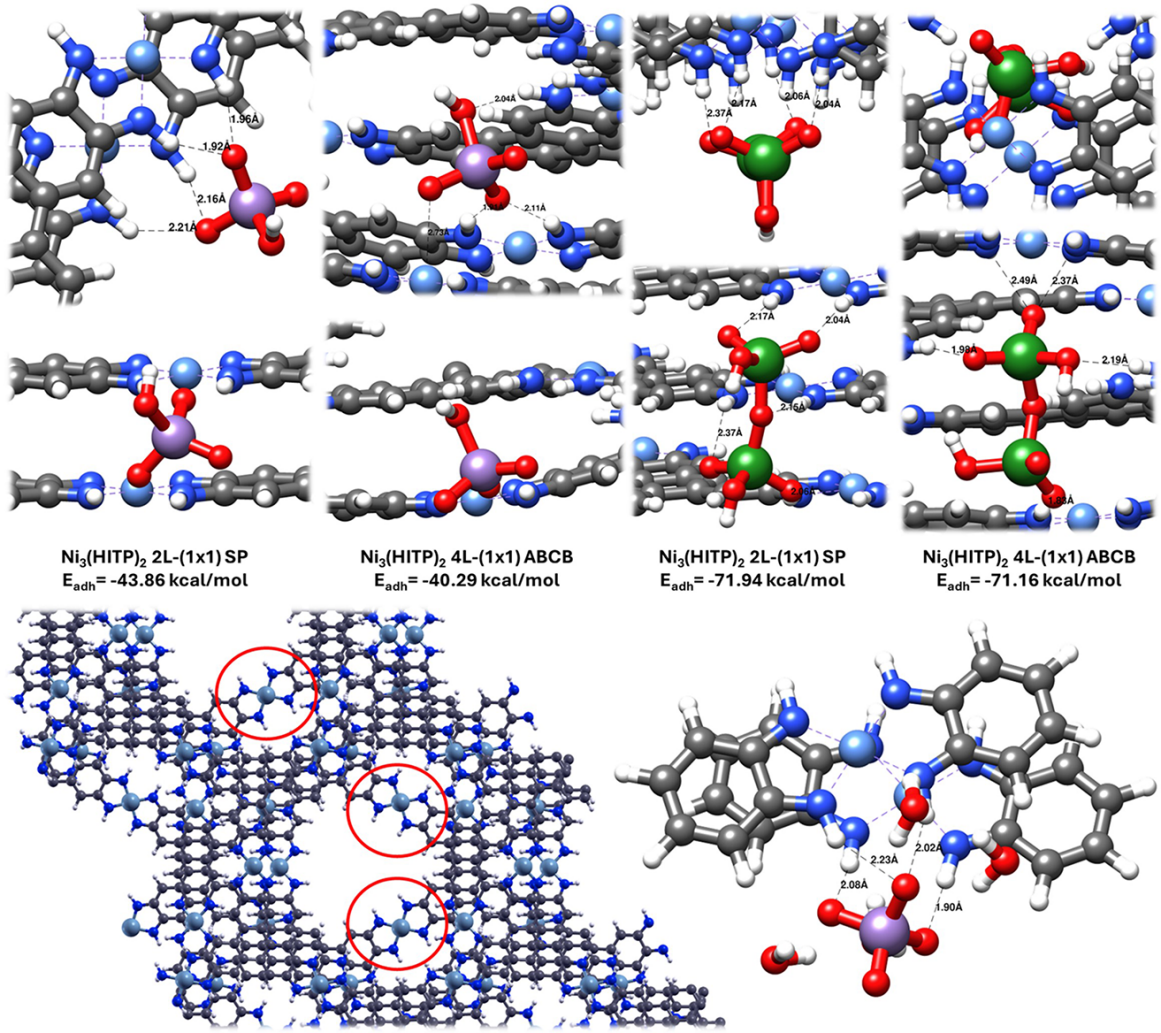
(Top panel) Optimized configurations and correspondent adhesion energies (in kcal/mol) of the HMnO_4_ and H_2_Cr_2_O_7_ adsorbates interacting with the Ni_3_(HITP)_2_ system in both SP arrangement and ABC reconstruction. (Bottom panel) On the left, the structure of the unit cell, replicated twice in both x and y directions, of the Ni_3_(HITP)_2_ MOF adopting ABC morphology; on the right, a finite model of two partial layers of the Ni_3_(HITP)_2_ material interacting with MnO_4_^–^ when surrounded by four water molecules; most characteristic distances (in Angstrom) are reported. Color coding: Ni light blue, O red, N blue, C gray, H white, Mn purple, and Cr green.

From the examination of the adsorption regions of the MOF, it can be noticed that Ni_3_(HITP)_2_ is terminated only by hydrogens. Those belonging to the amine moieties can be donors of hydrogen bonds. Both adsorbates have strong nucleophilic oxygens, which are potential acceptors of such a type of bonding. As confirmation, in the SP configuration, we observe the formation of strong hydrogen bonds between the nucleophilic O atoms of the guest and the H atoms of the metal core (distances between 1.9 and 2.2 Å), and we find that the interaction is roughly proportional to the number of bonds established. H_2_Cr_2_O_7_, in its elongated configuration in the MOF channel, exposing four O atoms to the electrophilic MOF hydrogens, is, in fact, characterized by an adhesion that is almost double the one where HMnO_4_ exposes only two oxygens. In the reconstructed structure, the situation is slightly different: the electrophilic oxygens of the guests can interact with the positive metal centers of OMS, although this further contribution does not change the values of adhesion energy much. We found a weak interaction between the O atoms of the guests and Ni; instead, the adsorbates preferred to maximize the formation of H bonds, which can provide very similar adhesion energy to that characterizing the SP arrangement for both guest species. The obtained values for both morphologies are compatible with the pseudo-second-order kinetic models, suggesting an interaction of chemical type between the adsorbates and the MOF.

For a further comparison, we also examined the interaction of both adsorbates with the less performant MOF, i.e., Cu_3_(HHTP)_2_. In agreement with the experimental findings, the weaker adhesion energies can be due to the hydrogens of the linkers and oxygens in the metal core; repulsive interactions between the O-terminated metal core and the nucleophilic guests move the adsorbates to longer distances from the metal core and are only partially compensated by weak attractive van der Waals dispersive forces between the guests and the H atoms at the edges of the linkers (distances around 2.2–2.3 Å). In the reconstructed structure of the MOFs, we observe an increased interaction between the adsorbates and the Cu centers, although the final values of the adhesion energies (Figure S70) are still well below the strength of those characterizing the Ni_3_(HITP)_2_ system.

To estimate the effect of water on the sorption performances of Ni_3_(HITP)_2_, we included four water molecules in the optimized geometry of HMnO_4_ adsorbed in the SP MOF structure and reoptimized the whole complex. The added molecules created a network of hydrogen bonds (Figure S71), inducing a slight elongation of the direct connections between the adsorbate and the MOF. The adhesion between the adsorbate and the MOF in the configuration perturbed by the water molecules was approximately −43.3 kcal/mol, which was very similar to that estimated in the absence of water (−43.9 kcal/mol). This is further proof of the adaptability of the Ni_3_(HITP)_2_ framework in maximizing its interaction with adsorbate species. We also performed a test calculation on the MnO_4_^–^ ion by using the finite model shown in the bottom panel of Figure 5, and we observed that the ion also undergoes the same slight elongation of the adsorbate/MOF equilibrium bond lengths induced by water in the neutral counterpart.

### *In Situ* Growth of Ni_3_(HITP)_2_ on Cotton Textile

Despite its promising and record-high adsorption capabilities, Ni_3_(HITP)_2_ crystals, similar to other MOF powders, present challenges for practical applications due to their small crystal size and tendency to agglomerate in solution.^49^ These challenges not only complicate the handling, deployment, and recovery of the materials in conventional water filtration systems, but also limit their reusability, leading to material loss over successive adsorption cycles. To overcome these limitations, we sought to immobilize Ni_3_(HITP)_2_ onto textile substrates, which would not only improve handling and flexibility, but also enable reusability across multiple adsorption cycles. Our group previously reported the deposition of Ni_3_(HITP)_2_ on cotton via a direct solution-phase self-assembly approach.^57^ While the resulting MOF-textile composite demonstrated promising chemiresistive gas sensing, uptake, and filtration performance, it suffered from significant mass loss and high sheet resistance upon handling, which could limit its suitability for both, water remediation and oxyanion sensing. Thus, we developed a continuous stepwise layer-by-layer (LbL) deposition method. While this method is well-established for achieving robust MOF anchoring onto textile surfaces, it has not been previously employed for anchoring cMOFs onto textile fabrics.^111–113^ This approach, illustrated in Figure 6a, involved the sequential immersion of plasma-cleaned 5 × 2 cm^2^ cotton swatches in a) a metal-containing solution and b) a ligand-containing solution, followed by intermediate washing steps. Repeating this process for a total of 10 LbL cycles resulted in uniform Ni_3_(HITP)_2_ growth on the cotton fabric. The mass difference of the fabric measured before and after MOF deposition indicated a loading of 8.5 ± 1.5 mg of Ni_3_(HITP)_2_ per cm^2^ of textile, corresponding to approximately a 2.5-fold increase in the mass of the pristine textile fabric (see Section S7 for experimental details).

**Figure 6:**
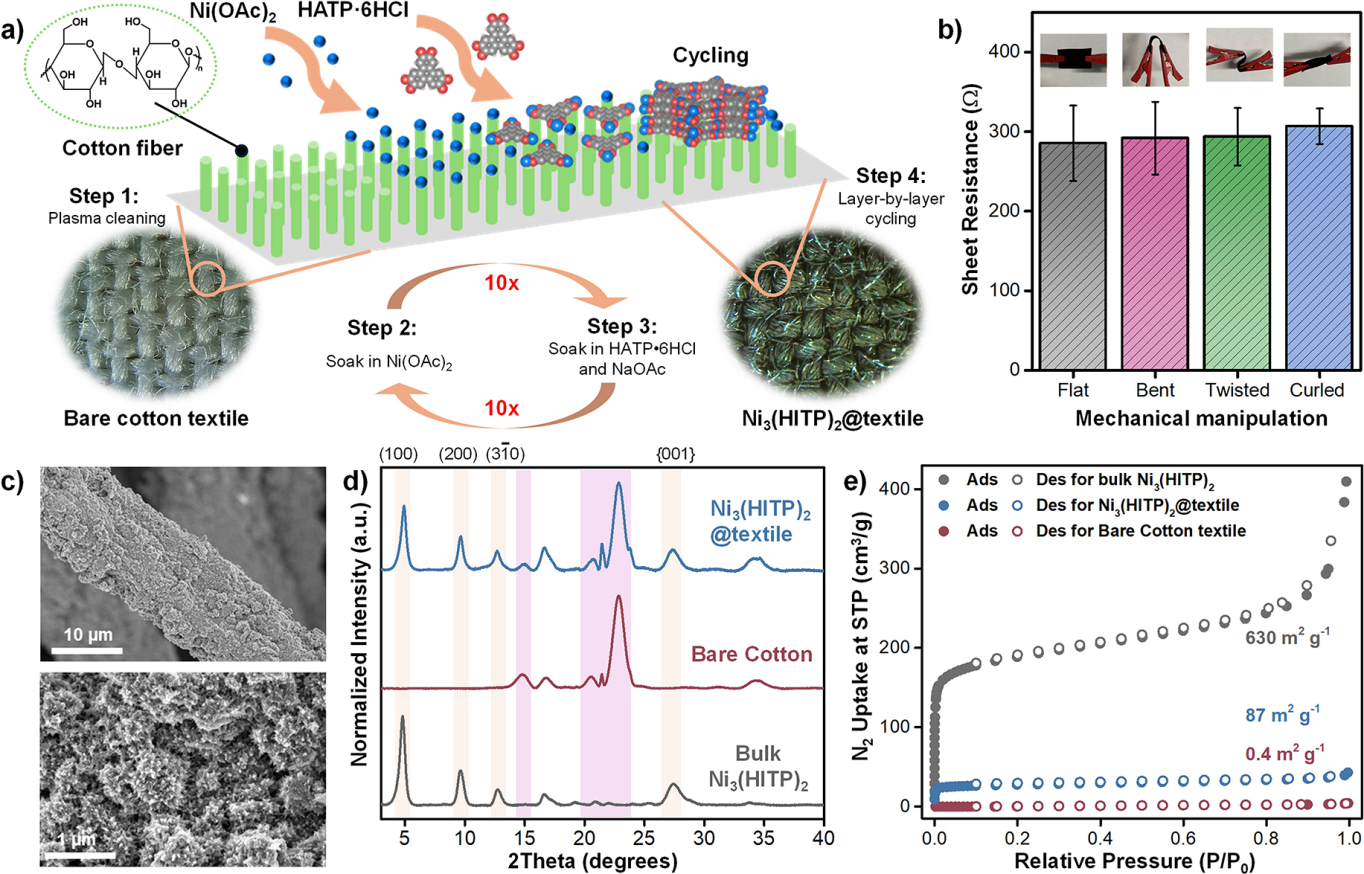
a) Schematic illustration of the preparation procedure of Ni_3_(HITP)_2_@textile via a layer-by-layer deposition method. b) Sheet resistance values of Ni_3_(HITP)_2_@textile swatches (2 cm × 1 cm) upon mechanical manipulation, showcasing their consistent electronic performance under physical stress. Inset: photographs of mechanically manipulated Ni_3_(HITP)_2_@textile swatches. c) SEM micrographs of Ni_3_(HITP)_2_@textile at two different magnifications. d) PXRD traces and e) BET surface area measurements of bare cotton (red), bulk Ni_3_(HITP)_2_ (gray), and Ni_3_(HHTP)_2_@cotton (blue).

PXRD analysis of the resulting composite, termed Ni_3_(HITP)_2_@textile, confirmed the successful formation of crystalline MOF on the cotton fabric, as evidenced by the high relative intensities of the (100), (200), and (001) crystallographic planes, which closely matched those of the bulk MOF (Figure 6d). SEM measurements, combined with elemental mapping revealed the formation of rod-like crystals uniformly coating the textile surface (Figures 6c and S72–S73). ATR-IR and XPS measurements confirmed the presence of the Ni_3_(HITP)_2_ coordination network on the fabric swatches (Figures S74–S75). Sheet resistance measurements, performed using a two-point probe, demonstrated the high stability and preserved functional properties of the swatches under various mechanical manipulations, with swatches achieving a sheet resistance of ≈ 0.3 kΩ per cm^2^ under all tested conditions (Figures 6b and S76–S79). Nitrogen adsorption–desorption isotherms, shown in Figures 6e and S80, revealed a 290-fold increase in the BET surface area, from 0.3 m^2^ g^–1^ for bare cotton up to 87 m^2^ g^–1^ for Ni_3_(HITP)_2_@textile, highlighting the substantial enhancement in porosity of the textile fabric upon MOF deposition. Additionally, scotch tape test confirmed the exceptional adhesion of Ni_3_(HITP)_2_ to the cotton textile, as indicated by negligible mass loss and unchanged resistance values post-test (Figures S81–S82).

To assess the adsorption capabilities and filtration rate of the fabricated MOF membranes, we utilized Ni_3_(HITP)_2_@textile swatches as filtration media, following the setup illustrated in Figure 7a. We placed the swatches in a glass core sand crucible, and allowed aqueous solutions of Cr_2_O_7_^2–^ (30 ppm) or MnO_4_^–^ (10 ppm) solutions to pass through them under vacuum to facilitate oxyanion transport.^114^ We selected these concentrations based on reported cases of potable and irrigation water contamination involving manganese and hexavalent chromium species, which typically fall within the low ppm range of 4 to 20 ppm.^90,91^ We found Ni_3_(HITP)_2_@textile to efficiently filter out 96% of a 10 ppm MnO_4_^–^ solution and 81% of a 30 ppm of Cr_2_O_7_^2–^ solution in less than 1 min (Figures 7b and S83), highlighting the composite potential in remediation systems requiring rapid and effective filtration. To quantitatively compare the adsorptive performance of the bulk and composite forms of Ni_3_(HITP)_2_, we conducted batch adsorption experiments using 100 ppm oxyanion solutions. We immersed 1 cm^2^ swatches of Ni_3_(HITP)_2_@textile, corresponding to a MOF loading of 8.5 ± 1.5 mg, in 13 mL of 100 ppm oxyanion solutions and stirred them at room temperature for 24 h, maintaining a volume/mass ratio of 1.5. ICP-MS analysis of the supernatants revealed 99% removal for MnO_4_^–^ and 73% for Cr_2_O_7_^2–^, comparable to the adsorption efficiencies of bulk Ni_3_(HITP)_2_, while PXRD and SEM measurements revealed the retention in morphology and crystallinity of the MOF composite (Figures 7c and S84–S88). Cyclic adsorption–desorption experiments using 25 ppm oxyanion solutions, shown in Figure 7d-e, demonstrated that Ni_3_(HITP)_2_@textile retained removal efficiencies above 94% for both, MnO_4_^–^ and Cr_2_O_7_^2–^, over 32 consecutive cycles. Interestingly, the deposition of Ni_3_(HITP)_2_ on textiles significantly improved the reusability and long-term operational stability of the MOF. Compared to bulk Ni_3_(HITP)_2_, which exhibited nickel leaching levels of 2.19 and 2.11 ppm after exposure to 20 ppm of MnO_4_^–^ and Cr_2_O_7_^2–^, respectively, Ni_3_(HITP)_2_@textile demonstrated a significantly reduced nickel leaching, averaging 4.1 ± 1.5 ppb and 9.9 ± 3.6 ppb when exposed to 25 ppm of MnO_4_^–^ and Cr_2_O_7_^2–^, respectively (Figure S89). These values are well below the safe drinking water limits for nickel set by the WHO (70 ppb) and U.S. EPA (100 ppb) agencies, underscoring the potential of Ni_3_(HITP)_2_@textiles for practical water filtration applications.^115^ Based on initial leaching values, MOF@textile composite can sustain up to ≈16 continuous adsorption cycles with Cr_2_O_7_^2–^ and 8 cycles with MnO_4_^–^ before nickel levels approach the WHO drinking water threshold, demonstrating good recyclability and safe operational performance over repeated use. Additionally, a 1 cm × 2 cm swatch of Ni_3_(HITP)_2_@textile continuously filtered up to 115 mL of MnO_4_^–^ solution (10 ppm) and 40 mL of Cr_2_O_7_^2–^ solution (10 ppm) while maintaining >90% removal efficacy, without any intermediate washing or regeneration (Figure S90). PXRD and ATR-IR measurements indicated that crystallinity is still present and the coordination network of Ni_3_(HITP)_2_ remains intact on the textile, suggesting structural robustness during the continuous filtration experiments (Figure S91).

**Figure 7:**
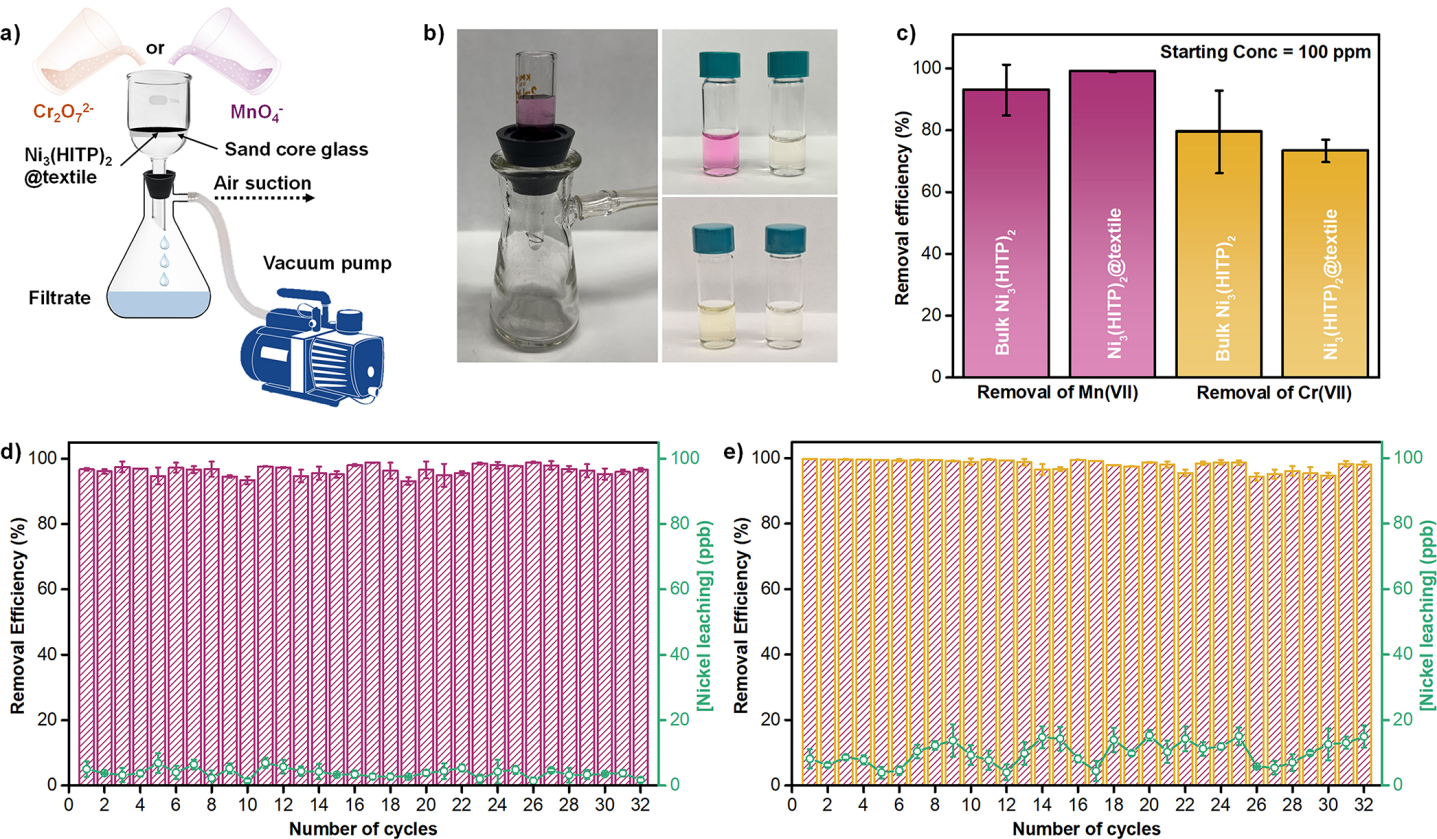
Proof-of-concept filtration tests for MnO_4_^–^ and Cr_2_O_7_^2–^ through Ni_3_(HITP)_2_@textile swatches. a) Schematic illustration of the filtration assembly, showcasing the vacuum filtration system employed. b) Photographs of the filtration system and the oxyanion solutions, before, and after filtration. c) Comparison of the removal efficiency of bulk Ni_3_(HITP)_2_ and Ni_3_(HITP)_2_@textile swatches toward 100 ppm of oxyanion solutions. Cyclic adsorption tests of Ni_3_(HITP)_2_@textile to 25 ppm solutions of d) MnO_4_^–^ and e) Cr_2_O_7_^2–^.

### Chemiresistive Detection of Oxyanions Using E-textile

Encouraged by the robustness and conductive nature of Ni_3_(HITP)_2_@textile, we sought to utilize this composite as an electrochemical sensor for detecting MnO_4_^–^ and Cr_2_O_7_^2–^ in water. We hypothesized that this approach would eliminate the need for sample pretreatment, such as drying and extraction, providing a faster and more cost-effective alternative to conventional water quality monitoring techniques. To evaluate its potential, we designed an electrochemical sensing setup, employing the MOF@textile composite as a chemiresistor (Figure S92). In brief, we partially immersed a swatch of Ni_3_(HITP)_2_@textile in water, applied a constant potential at both ends of the swatch, and allowed the system to equilibrate until we observed a constant current (see Section S8). Upon the subsequent additions of different concentrations of MnO_4_^–^ or Cr_2_O_7_^2–^ to the system, we recorded an increase in the output current within seconds, indicating a rise in the conductivity of the MOF composite due to its interaction with the oxyanions (Figures 8 and S93–94). Estimating the theoretical limit of detection (LoD) from these experiments using the protocol described in Section S8.4, we determined LoDs of 2.2 ± 1.1 ppm for MnO_4_^–^ and 6.0 ± 4.5 ppm for Cr_2_O_7_^2–^ (Figures S95–S96). These results suggested that Ni_3_(HITP)_2_@texitle can achieve dual sensing and capture with high efficiency, outperforming most reported fluorescent MOF materials in adsorption capacity while demonstrating moderate sensing capabilities (Tables S2–S3). To further assess the suitability of Ni_3_(HITP)_2_@textile for real-world applications, we evaluated additional sensing parameters, including sensitivity and selectivity. We estimated the sensitivity, defined as the change in output signal per unit concentration of the analyte to be 0.49 μA ppm^–1^ for MnO_4_^–^ and 0.47 μA ppm^–1^ for Cr_2_O_7_^2–^, suggesting effective oxyanion detectability and signal transduction. Moreover, we found the sensor to retain its sensing capability toward MnO_4_^–^ even in the presence of 50 ppm sulfate ions, whereas a control experiment using carbon cloth showed insignificant response to oxyanion addition (Figures S97–S99). Overall, the low ppm-level LoD, high sensitivity, rapid response time, and selectivity in the presence of interfering species highlight the potential of Ni_3_(HITP)_2_@textile as a promising candidate for real-time water quality monitoring.

**Figure 8:**
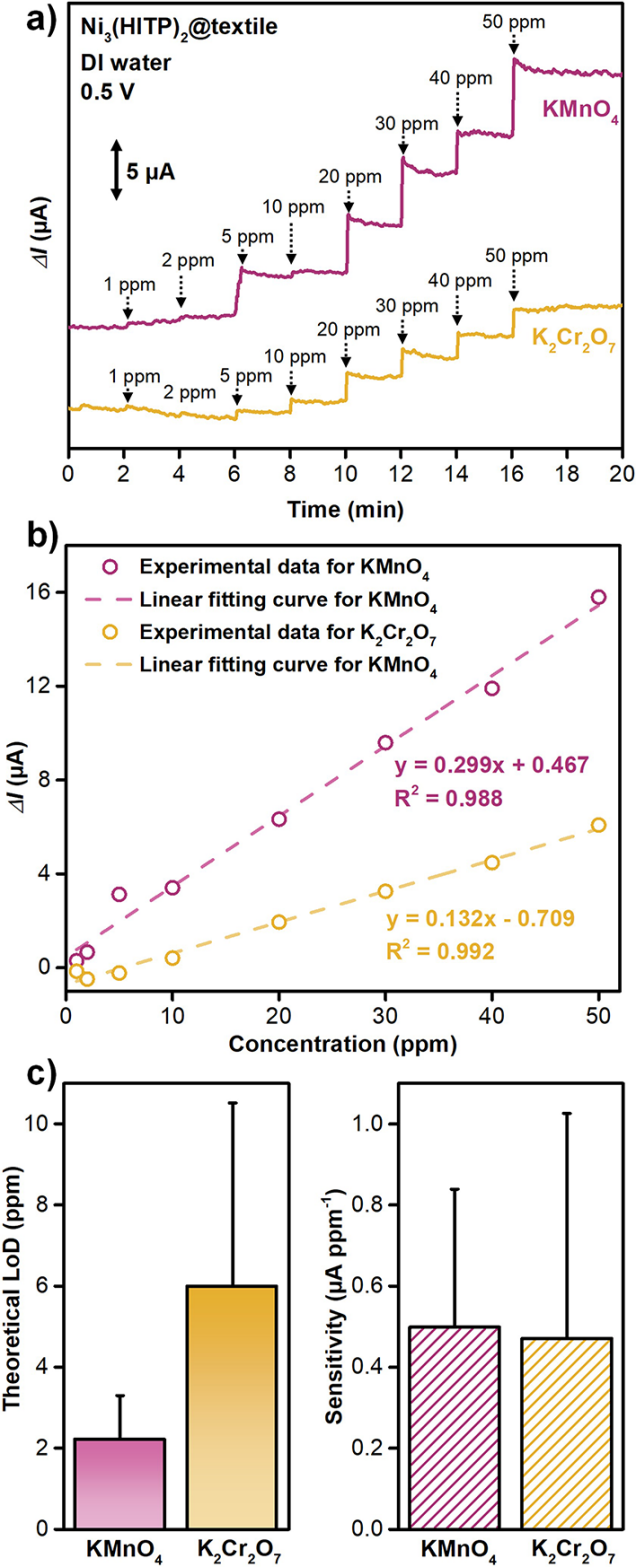
Chemiresistive detection of oxyanions with Ni_3_(HITP)_2_@textile swatches. a) Amperometric sensing traces showing the change in the current response of Ni_3_(HITP)_2_@textile at 0.5 V upon the successive additions of aliquots of K_2_Cr_2_O_7_ and KMnO_4_ in DI water. b) Response (change in current) vs concentration curves of Ni_3_(HITP)_2_@textile swatches exposed to K_2_Cr_2_O_7_ and KMnO_4_ in DI water. c) Calculated sensitivity (μA/ppm) and theoretical limit of detection (based on 3× S/N) for the MOF@textile swatches in response to each analyte of detection (based on 3× S/N). Error bars represent the standard deviation of three replicates.

While the MOF@textile demonstrated a chemiresistive response to increased ionic strength, the adsorption of oxyanions into the framework can also be detected using impedance measurements, which have previously been employed for MOF-based detection of redox-inactive analytes.^116,117^ As a proof of concept, we used electrochemical impedance spectroscopy (EIS) to detect the adsorption of MnO_4_^–^ oxyanions by a thin film of Ni_3_(HITP)_2_, dropcasted onto a glassy carbon electrode (see Section S9). EIS was conducted before and after the addition of MnO_4_^–^ to the cell (Figures S100–S101), and the resulting data was fit to the equivalent circuit shown in the inset of Figure S100, based on previously reported EIS experiments with 2D cMOFs.^118^ We observed the impedance of the electrode to increase following exposure to 15.8 ppm KMnO_4_ in 0.1 M KCl (Figure S102). In contrast, the addition of KCl alone did not result in any increase in impedance, while a bare glassy carbon electrode showed a decrease in impedance in response to the addition of MnO_4_^–^ (Figure S103). The high selectivity of Ni_3_(HITP)_2_ toward the oxyanions, even in the presence of chloride and sulfate species, are likely arising from the favorable binding interactions between the oxyanions and host sites of the MOF. These interactions likely include (i) hydrogen-bonding interactions between the nucleophilic oxygen of oxyanions and hydrogen donor groups in HITP moieties, (ii) electrostatic interactions between oxyanions and the nickel­(bisdiimine) linkages, (iii) chemical interactions at the adsorptive sites of the MOF, and (iv) redox interactions, where the oxyanions are reduced and HITP is oxidized.

## 4 Conclusion

This work presents the first demonstration of triphenylene-based, layered cMOFs with varying metal nodes and functionalities for the dual sensing and filtration of oxyanions from water. The as-synthesized cMOFs exhibited exceptional adsorption performance, surpassing most previously reported MOF-based adsorbents. Among them, Ni_3_(HITP)_2_ demonstrated unprecedented adsorption capacities, filtering up to 827 mg of MnO_4_^–^ and 497 mg of Cr_2_O_7_^2–^ per gram of MOF, while displaying rapid kinetics, achieving 99% removal within 10 min. Examining the performance of Ni_3_(HITP)_2_ in various aqueous environments, further highlighted its selectivity and efficiency, demonstrating (i) adsorption capabilities across a broad pH range (4–10), (ii) high selectivity in the presence of competing anions, and (iii) consistent performance across diverse water matrices. Spectroscopic and computational investigations revealed a multifaceted scavenging mechanism, involving chemisorption, hydrogen-bonding, electrostatic interactions, and redox reactions. Additionally, depositing Ni_3_(HITP)_2_ onto cotton fabrics produced mechanically robust, flexible, multifunctional electronic textiles, capable of simultaneously capturing (for at least 32 adsorption cycles) and detecting Cr_2_O_7_^2–^ and MnO_4_^–^ at low ppm concentrations (2.2 ppm for MnO_4_^–^ and 6.0 ppm for Cr_2_O_7_^2–^). This durability underscores the potential of these MOF@textile composites for long-term, sustainable water filtration and sensing applications.

With these advantages in mind, we identified two key challenges that must be addressed to further advance the practical application of 2D cMOFs in water filtration. First, while conducting adsorption studies on the bulk MOF over a 24-h period, we observed significant nickel ion leaching and structural degradation, with concentrations reaching as high as 10 ppm at elevated Mn­(VII) and Cr­(VI) levels (Figure S89). Given the fast kinetics demonstrated by these MOFs in oxyanion removal, reducing exposure time is thus necessary. Further studies should determine the optimal exposure duration for complete oxyanions remediation, while minimizing framework degradation. Second, the sensing experiments we performed relied mostly on changes in the ionic strength of the contaminated solution, which were monitored via impedance and chemiresistive measurements. While these proof-of-concept experiments demonstrated the feasibility of using these frameworks as sensors, they remain less sensitive compared to fluorescent-based sensors. To achieve ultrasensitive and selective detection, enhancing device fabrication and integration is essential.^119^ Taken together, we believe our study lays the foundation for the development of next-generation environmental remediation technologies for use as point-of-use (POU) devices and portable filtration systems. By combining high-efficiency adsorption with real-time sensing, Ni_3_(HITP)_2_ cMOF not only purifies water but also enables continuous monitoring, making this class of materials highly promising for advanced water treatment applications in both fixed installations and mobile units.

## Supplementary Material











## Notes

Experimental methods, additional characterization, adsorption capacities, kinetic models, computational data, electrochemical sensing measurements (PDF)
Mn­(VII) filtration demo (MPG)
Control experiment for Mn­(VII) filtration (MPG)
Cr­(VI) filtration demo (MPG)
Control experiment for Cr­(VI) filtration (MPG)
