# Supplementary material for: Electronic Textiles Based on Conductive Metal–Organic Frameworks as Scavengers and Sensors of Toxic Oxyanions from Water
Source: J Am Chem Soc. 2025 Jul 28;147(31):27561–75. doi: 10.1021/jacs.5c05275 (PMC12333345; doi:10.1021/jacs.5c05275)
Supplement: Supplementary file 1 [file ja5c05275_si_001.pdf]

Electronic Textiles Based on Conductive Metal−Organic Frameworks as Scavengers and Sensors of Toxic Oxyanions from Water

Patrick Damacet[1#], Priyanshu Chandra[1#], Emma K. Ambrogi[1], Hyuk-Jun Noh[1], Ericka L. Asmus[1], Elissa O. Shehayeb,[1] Giovanni Barcaro[2], and Susanna Monti[3*], and Katherine A. Mirica[1*]

1 Department of Chemistry, Burke Laboratory, Dartmouth College, Hanover, New Hampshire 03755, United States

2 CNR-IPCF, Institute for Chemical and Physical Processes, Area della Ricerca, Pisa I-56124, Italy

3 CNR-ICCOM, Institute of Chemistry of Organometallic Compounds, Area della Ricerca, Pisa I56124, Italy

[#]Equal Contribution

Susanna Monti-Email: susanna.monti@cnr.it

Katherine A. Mirica-Email: katherine.a.mirica@dartmouth.edu

Table of Contents

1. Materials and methods 3

2. Synthesis and characterization of molecular precursors and 2D cMOFs 4

2.1 Synthesis and characterization of HATP·6HCl 4

2.2 Synthesis and characterization of $Ni_3(HHTP)_2$ 6

2.3 Synthesis and characterization of $Cu_3(HHTP)_2$ 7

2.4 Synthesis and characterization of $Ni_3(HITP)_2$ 8

2.5 Synthesis and characterization of $Cu_3(HITP)_2$ 9

2.6 Energy-dispersive X-ray spectroscopy 10

2.7 Characterization of the 2nd batch of MOFs 12

2.8 Particle size analysis based on SEM micrographs 14

2.9 Thermogravimetric analyses of MOFs 15

3. Adsorption experiments 16

3.1 Batch adsorption experiments 16

3.2 Maximum adsorption capacities (Q_{max}) of MOFs 16

3.2.1 Maximum adsorption capacities for MnO_4^- 17

3.2.2 Maximum adsorption capacities for $Cr_2O_7^{2-}$ 18

3.3 Surface charge of MOFs 20

3.3.1 Dye adsorption experiments 20

3.3.2 Zeta potential measurements 21
3.4 Adsorption kinetic studies 24
3.4.1 Kinetic isotherms for MnO_4^- 26
3.4.2 Kinetic isotherms for $Cr_2O_7^{2-}$ 29
3.5 Oxyanion removal under diverse aquatic environments 31
3.5.1 Effect of co-existing anions 31
3.5.2 Effect of pH 32
3.5.3 Selectivity towards different manganese and chromium species 33
3.5.4 Recyclability of $Ni_3(HITP)_2$ 34
4. Mechanistic insights into the MOF-oxyanion interactions 36
4.1 ATR-FTIR spectroscopy 36
4.2 SEM-EDX 37
4.3 XPS 43
4.4 Additional Characterization 45
5. Simultaneous adsorption 46
6. Multi-scale modeling 47
7. Electronic textiles based on $Ni_3(HITP)_2$ 51
7.1 Fabrication method 51
7.2 Characterization 54
7.3 Filtration performance and characterization post-adsorption 58
8. Chemiresistive detection of oxyanions 63
8.1 Sensing setup 63
8.2 Replicates of chemiresistive detection of MnO_4^- 64
8.3 Replicates of chemiresistive detection of $Cr_2O_7^{2-}$ 65
8.4 Calculation of the theoretical limit of detection (LoD) 66
8.4.1 Method 1 66
8.4.2 Method 2 67
8.4.3 Comparison of LoDs 68
8.5 Comparison of LoDs with literature 69
8.6 Chemiresistive detection of oxyanions in the presence of an interference 70
8.7 Control experiments 71
9. Impedance-Based Sensing with $Ni_3(HITP)_2$ films 71
10. References 74

1. Materials and methods

Triphenylene, racemic-BINAP, tris(dibenzylideneacetone)dipalladium(0), benzophenone imine, celite 545, toluene (lab grade), dichloromethane (99.6%, ACS grade), 2,3,6,7,10,11-hexahydroxytriphenylene Hydrate (HHTP), and tetrahydrofuran (99%, extra pure) were purchased from ThermoFisher Scientific. Sodium tert-butoxide, liquid bromine (reagent grade), copper(II) trifluoroacetyl acetonate, catalytic iron shavings, hexane (ACS reagent grade), and *N,N*-Dimethylacetamide (>99.9%) were acquired from Sigma-Aldrich. Copper(II) sulfate pentahydrate was purchased from Carolina. Potassium dichromate and potassium permanganate were acquired from Fisher Scientific. Hydrochloric acid, nickel(II) acetate tetrahydrate, and nitrobenzene (99%) were obtained from VWR. Dichlorobenzene (99%, HPLC grade), and diethyl ether (>99%, ACS grade) were purchased from Lab Alley.

Powder X-ray diffraction (pXRD) measurements were collected on a Rigaku sixth generation MiniFlex X-ray diffractometer with a 600 W (40kV, 15 mA) CuKα (α = 1.54 Å) radiation source following background subtraction. The range between 3° and 45° 2Θ was scanned with a step size of 0.02° and a scan rate of 2° per minute. Inductively coupled plasma Mass spectroscopy (ICP-MS) was collected on the 8900-ICP-MS with the PrepFast M5 autosampler/autodilution system. Integration times are 100 msecs - 500 msecs, generally minimum of 3 reps and 100 sweeps per reading. NMR spectra were recorded on a Bruker 600 MHz NMR spectrometer. Scanning Electron Microscopy (SEM) measurements were performed on a Thermo Scientific Helios 5CX Dual Beam instrument equipped with UltimMax 100 X-Ray detector and an EBSP camera. X-ray photoelectron spectroscopy (XPS) measurements were carried out on Kratos Analytical AXIS Supra X-ray Photoelectron Spectrometer under ultrahigh vacuum (base pressure 10^{-7} Torr) equipped with a monochromatic Al (Kα) X-ray source. All survey and high-resolution spectra were obtained using a beam diameter of 200 µm. Thermogravimetric analysis (TGA)

measurements were carried out on a TGA 55 instrument with 10°C/min ramp rate and nitrogen (N_2) as the purging gas. Brunauer-Emmett-Teller (BET) nitrogen gas measurements were performed on a 3Flex (Micromeritics, Norcross, Georgia) instrument at 77 K. Attenuated total reflectance infrared spectroscopy (ATR-IR) was performed on a Nicolet IS50 Spectrometer with a DLATGS detector. Electrical resistance measurements were collected on a KAIWEETS ST600Y digital multimeter smart auto range voltmeter. Mass Spectra were carried out on a Waters Synapt G2-Si Electron Spray Ionization (ESI) Mass Spectrometer. UV-Vis spectra were collected on a JASCO V-570 spectrophotometer at the scan rate of 200 nm min^{-1} under ambient conditions.

2. Synthesis and characterization of molecular precursors and 2D cMOFs

2.1 Synthesis and characterization of HATP·6HCl

Triphenylene-2,3,6,7,10,11-hexaamine hexahydrochloride (HATP·6HCl) was synthesized from triphenylene following a previously reported procedure.[1] The final product was characterized by ^{1}H-NMR (**Figure S1**), ^{13}C-NMR (**Figure S2**), and high-resolution mass spectroscopy (**Figure S3**, ESI method) to confirm its purity.

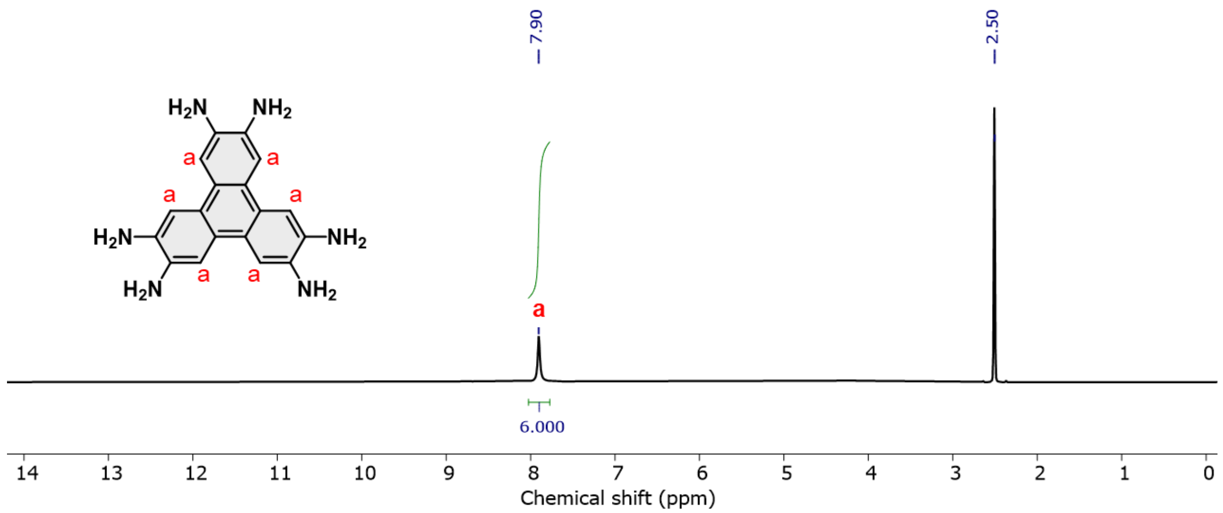

Figure S1. ^{1}H-NMR spectrum of HATP·6HCl in DMSO at 600 MHz.

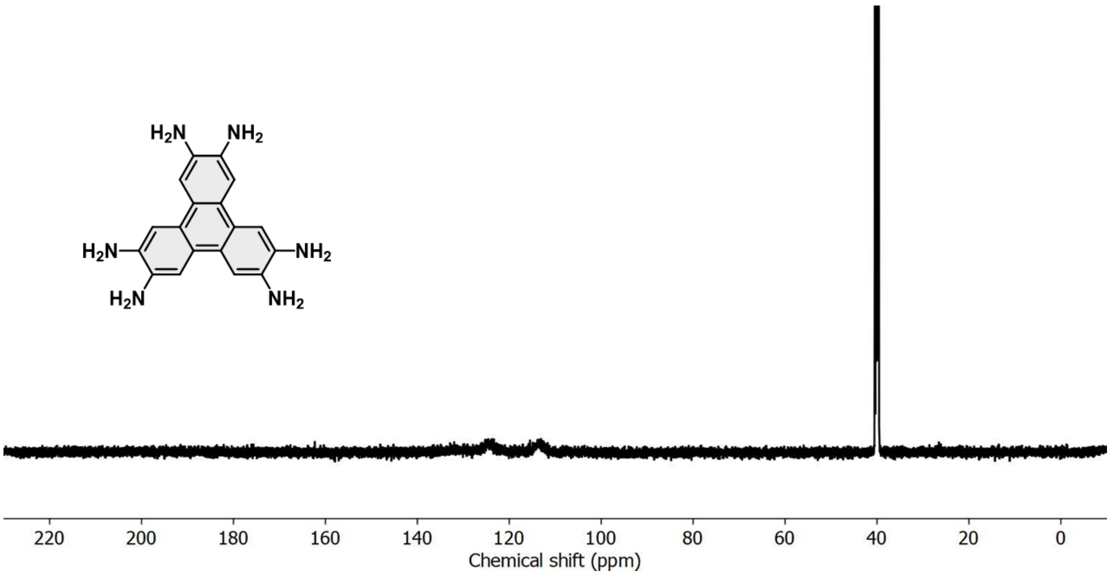

Figure S2. ^{13}C-NMR spectrum of HATP·6HCl in DMSO. The poor signal intensity is due to the low solubility of the ligand in DMSO.

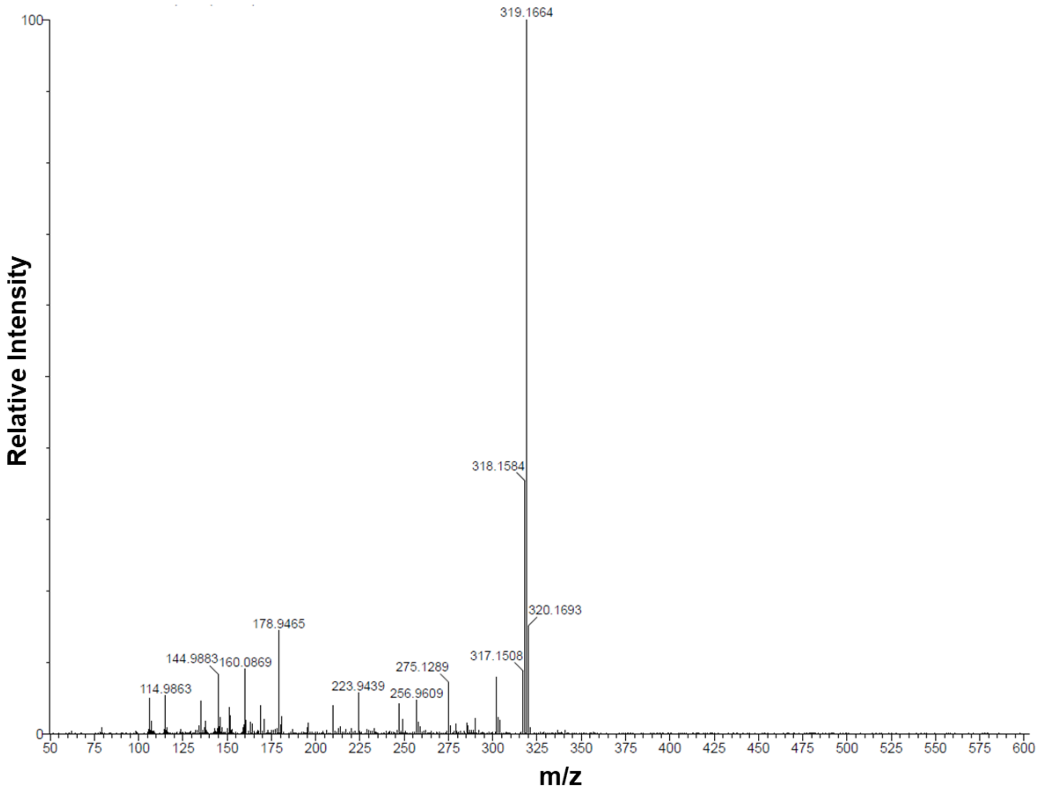

Figure S3. High-resolution mass specturm (ESI method) of HATP·6HCl.

2.2 Synthesis and characterization of $Ni_3(HHTP)_2$

$Ni_3(HHTP)_2$ was synthesized following a previously reported procedure with some modifications.[2] In brief, 20 mg of $Ni(OAc)_2 \cdot 4H_2O$ (0.08 mmol, 2 eq) was dissolved in 15 mL of DI water. To the metal solution, 13 mg HHTP (0.04 mmol, 1 eq.) was added. The resulting mixture was sonicated for 15 minutes, before being heated on a hot plate set at 85 °C for 12 hours. The resulting black powder, corresponding to $Ni_3(HHTP)_2$ MOF was filtered under vacuum followed by washing with DI water (15 mL), methanol (15 mL), ethanol (15 mL), and acetone (30 mL). The resulting powder was finally dried in a vacuum oven set at 72 °C for 24 hours.

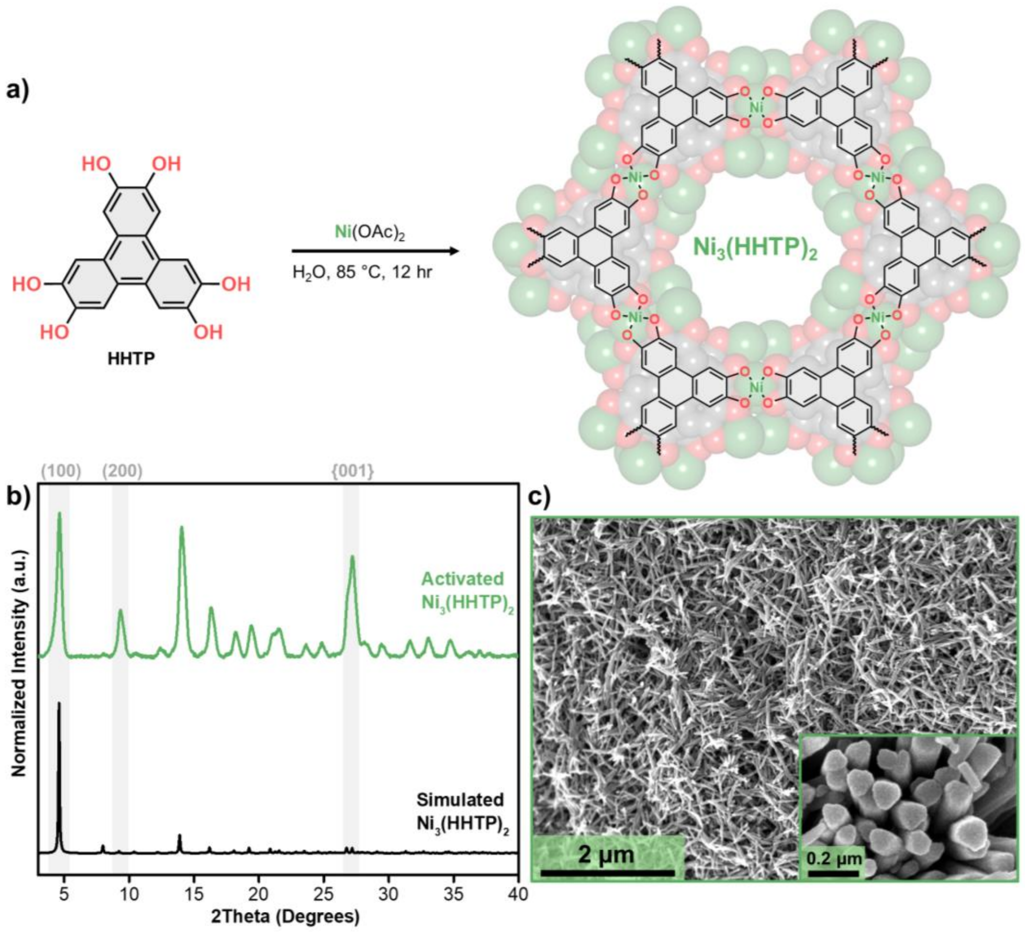

Figure S4. a) Synthetic scheme for the synthesis of $Ni_3(HHTP)_2$ MOF. b) PXRD patterns of simulated and activated $Ni_3(HHTP)_2$. c) SEM micrographs of $Ni_3(HHTP)_2$ after activation at two magnifications.

2.3 Synthesis and characterization of $Cu_3(HHTP)_2$

In an 8 mL scintillation vial, 2.3 mL of DI water was added to a powder mixture made of 15 mg of HHTP (0.046 mmol, 1 eq.) and 21 mg of copper trifluoroacetyl acetonate (0.057 mmol, 1.24 eq.). The resulting mixture was sonicated to allow for a homogeneous suspension. After 15 minutes, 0.21 mL of NMP was added dropwise to the mixture. The resulting mixture was sonicated for 2 mins and placed in a preheated oven set at 85 °C for 12 hours. The resulting powder (dark blue/black in color) was filtered, washed with DI water (30 mL), methanol (15 mL), ethanol (30 mL), and acetone (30 mL), before being dried in a vacuum oven set at 72 °C for 24 hours.

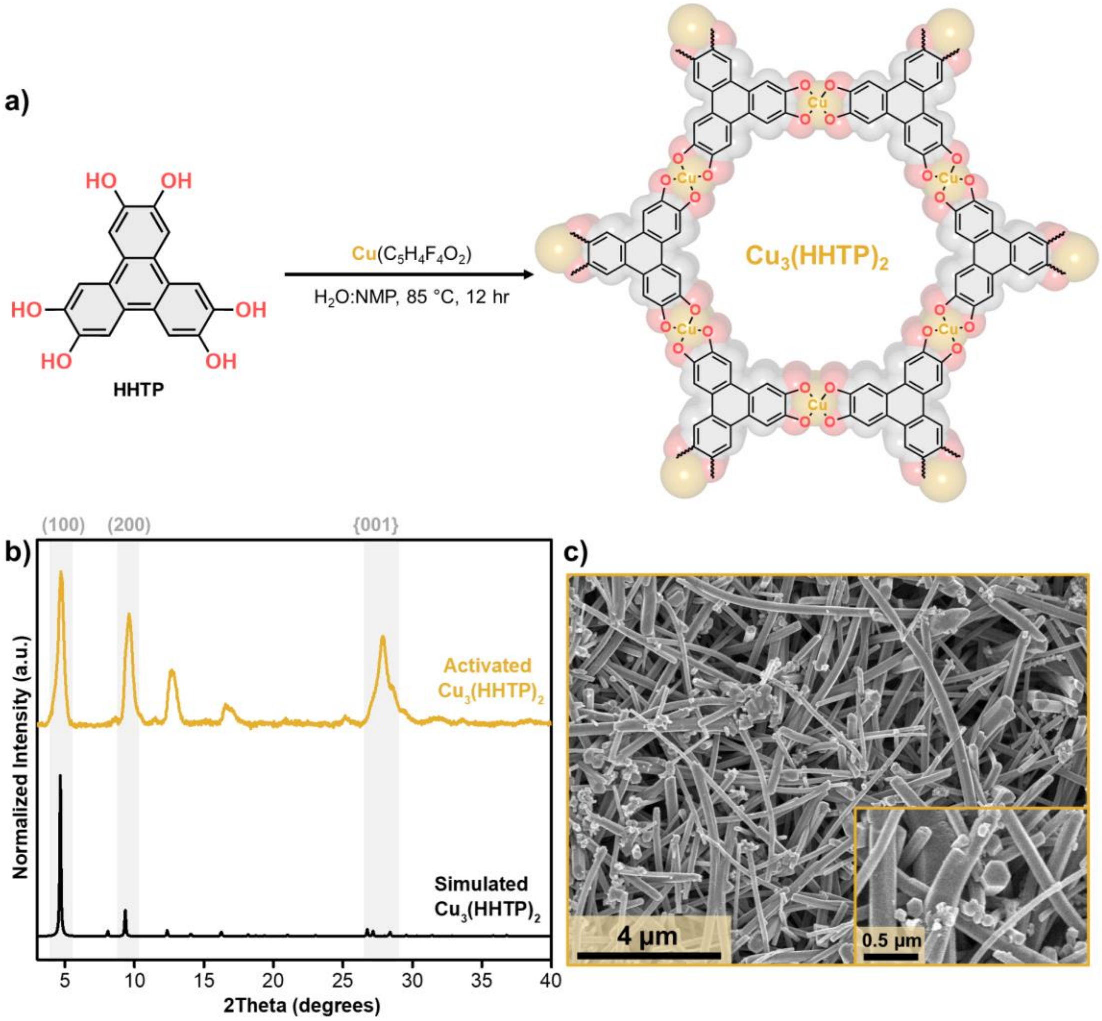

Figure S5. a) Synthetic scheme for the synthesis of $Cu_3(HHTP)_2$ MOF. b) PXRD patterns of simulated and activated $Cu_3(HHTP)_2$. c) SEM micrographs of $Cu_3(HHTP)_2$ after activation at two magnifications.

2.4 Synthesis and characterization of $Ni_3(HITP)_2$

$Ni_3(HITP)_2$ was synthesized as previously reported with some modifications.[3] In brief, 4.0 mg (0.016 mmol, 1.45 eq) of $Ni(OAc)_2{\cdot}4H_2O$ was dissolved in 6 mL of a 1:1 mixture of DMF:DMAC. The resulting solution was left heating on a hot plate set at 65 °C for 10 mins. 4 mL of an aqueous sodium acetate solution (containing 650 mg of NaOAc) was added to the metal salt solution, followed by a 1.5 mL aqueous HATP solution (0.011 mmol, 1.0 eq). The resulting solution was heated on a hot plate set at 65 °C for 2 hours while stirring. The resulting powder was filtered under vacuum, washed with 15 mL of DI water, 15 mL of methanol, and 30 mL of acetone, before being placed in a vacuum oven set at 72 °C for 24 hours.

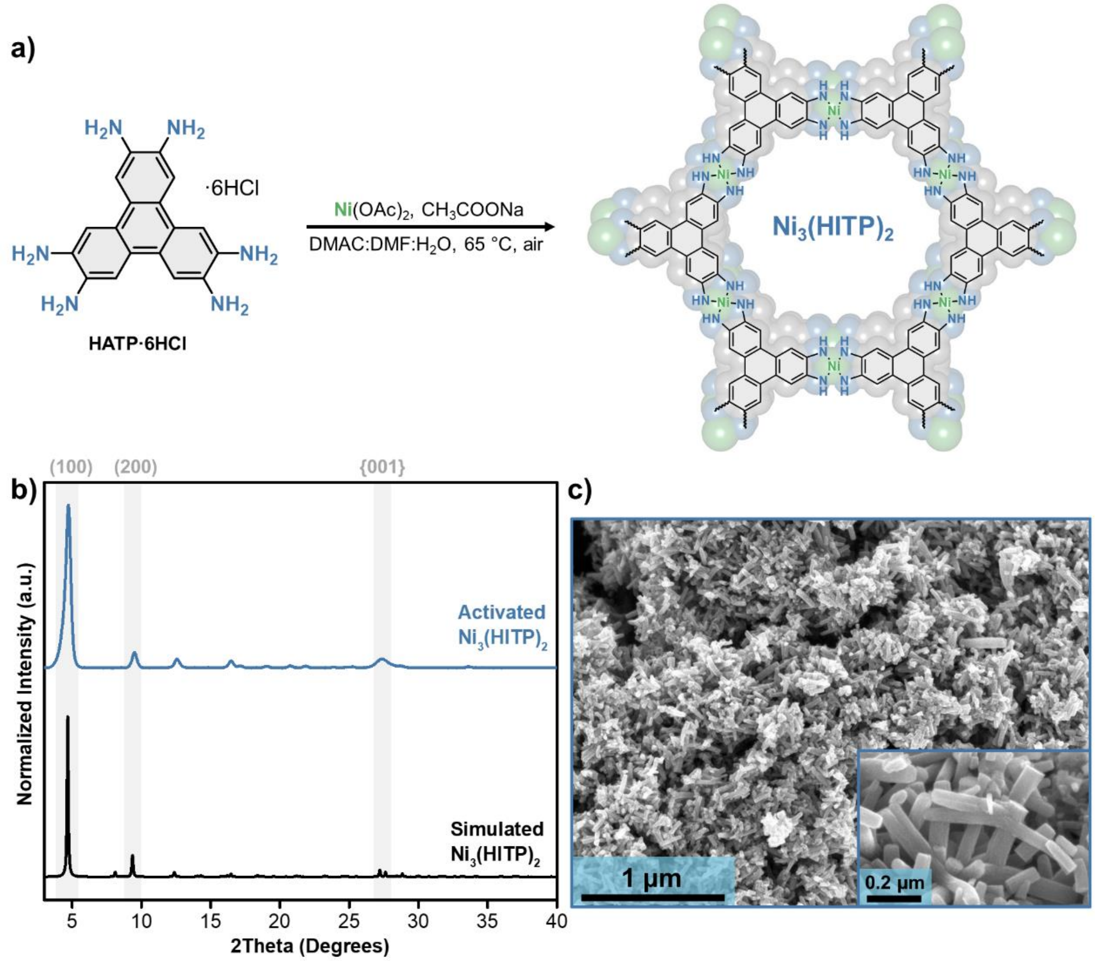

Figure S6. a) Synthetic scheme for the synthesis of $Ni_3(HITP)_2$ MOF. b) PXRD patterns of simulated and activated $Ni_3(HITP)_2$. c) SEM micrographs of $Ni_3(HITP)_2$ after activation at two magnifications.

2.5 Synthesis and characterization of $Cu_3(HITP)_2$

$Cu_3(HITP)_2$ was synthesized as previously reported with some modifications.[3] In brief, 4.1 mg (0.0164 mmol, 1.49 eq.) of $CuSO_4 \cdot 5H_2O$ was suspended in 1.7 mL of DMAC and sonicated for 20 min. To the suspension was added 6 mg (0.011 mmol, 1 eq.) of HATP·6HCl dissolved in 1.5 mL of DI water. The resulting mixture was sonicated for 5 mins. 328 g of sodium acetate, dissolved in 2 mL of DI water were added to the mixture. The resulting solution was shaken for a few seconds, before being placed on a preheated hot plate set at 65 °C for 2 hours while stirring. The resulting black powder was filtered under vacuum, washed with 10 mL of DI water, 10 mL of methanol, 20 mL of acetone, before being placed in a vacuum oven set at 72 °C for 24 hours.

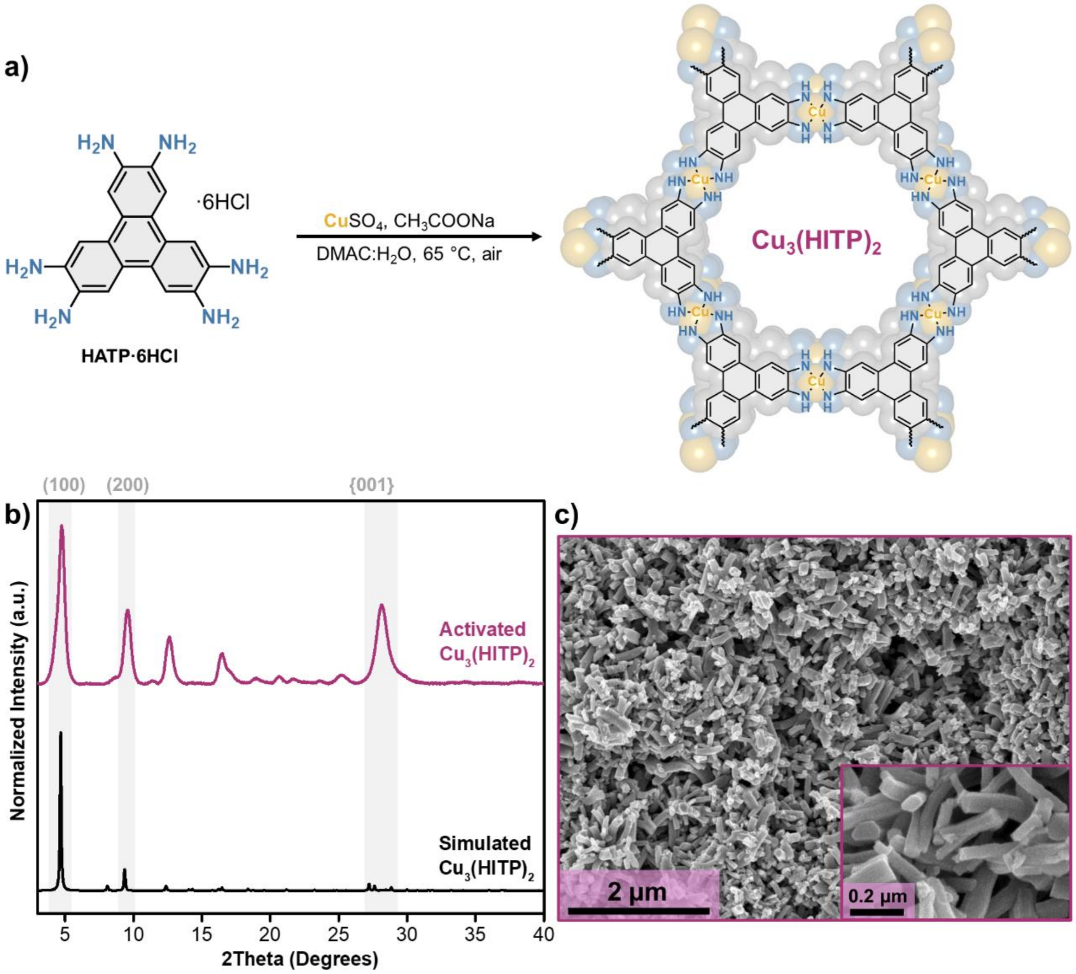

Figure S7. a) Synthetic scheme for the synthesis of $Cu_3(HITP)_2$ MOF. b) PXRD patterns of simulated and activated $Cu_3(HITP)_2$. c) SEM micrographs of $Cu_3(HITP)_2$ after activation at two magnifications.

Given the redox-active nature of HATP·6HCl and HHTP ligands, their exact oxidation states and electronic structures within the MOF structures can vary depending on the synthesis conditions and environmental exposure. This redox variability often leads to deviations from the idealized chemcial structures represented in **Figures S4-S7**. For further discussion on the electronic states of these ligands, we refer the readers to Ref.[4-7]

All studied MOFs were activated following the same procedure. Briefly, the MOF crystals were soaked in ethanol for 2 days, with the solvent being exchanged with fresh ethanol every 12 hours. The solvent was then exchanged with acetone following the same process. The resulting crystals were finally dried for 48 hours in a vacuum oven set at 75°C prior to characterization, adsorption, and sensing studies. The {001} set of crystallographic planes in the MOFs appeared slightly shifted compared to the simulated patterns, likely due to variations in interlayer spacing introduced by residual solvent molecules and/or stacking disorders during synthesis.

2.6 Energy-dispersive X-ray spectroscopy

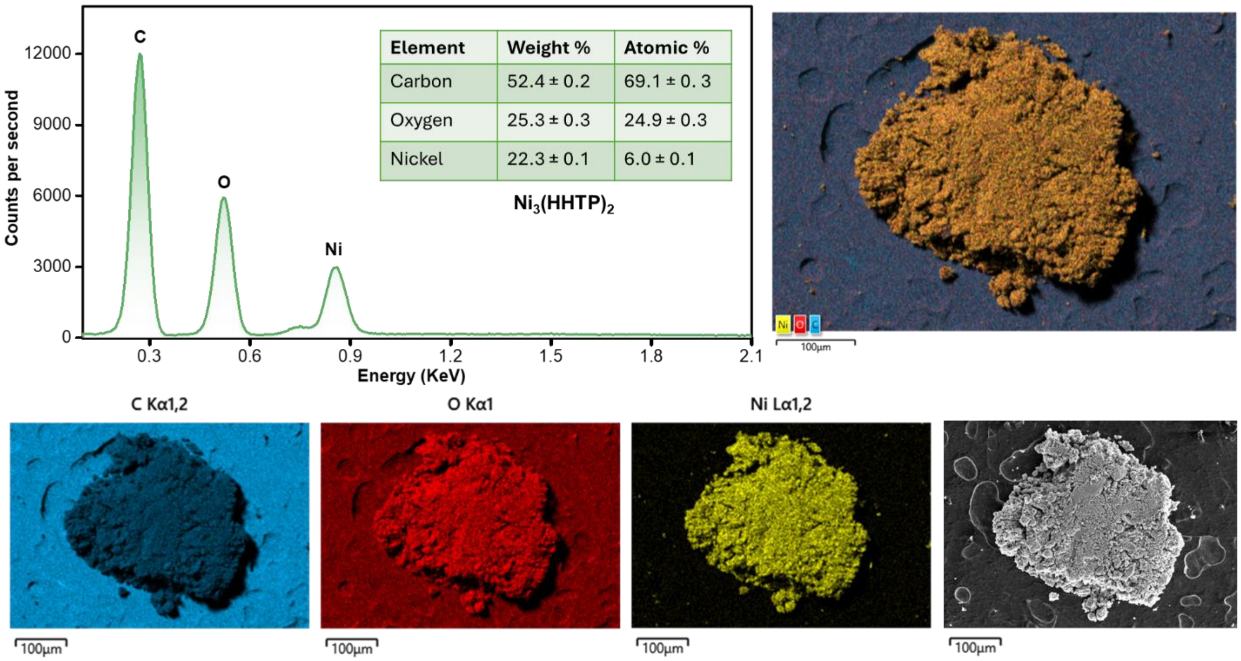

Figure S8. EDX spectrum and elemental mapping images of $Ni_3(HHTP)_2$.

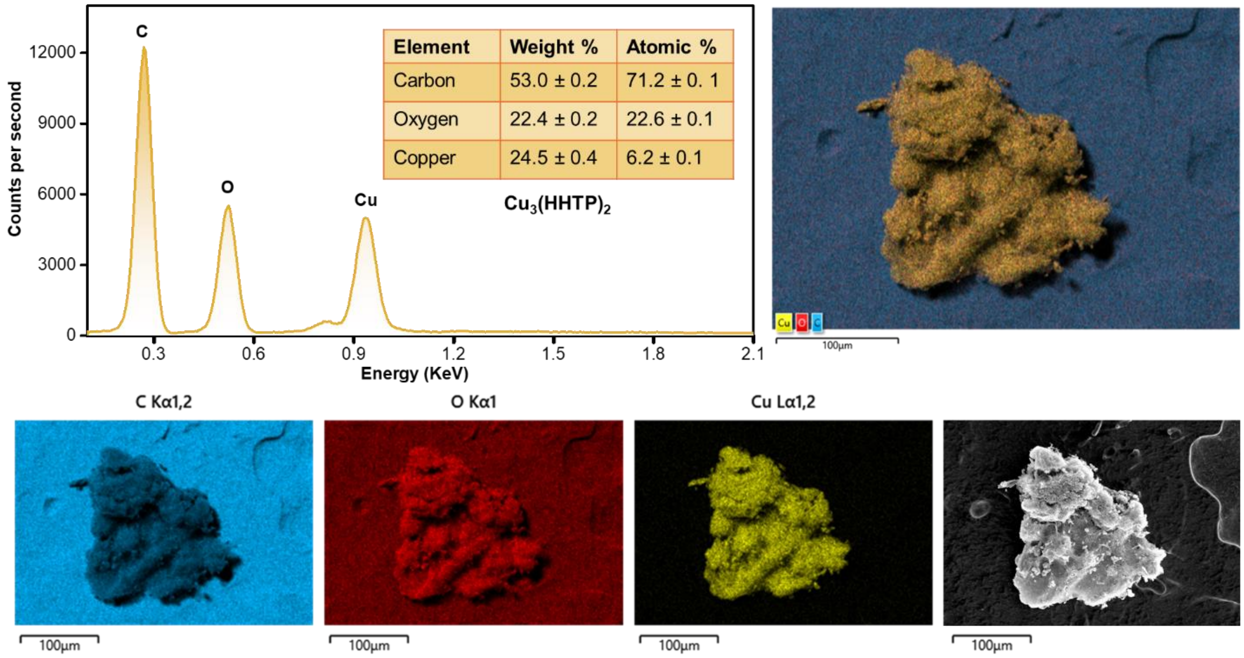

Element	Weight %	Atomic %
Carbon	53.0 ± 0.2	71.2 ± 0. 1
Oxygen	22.4 ± 0.2	22.6 ± 0.1
Copper	24.5 ± 0.4	6.2 ± 0.1

Figure S9. EDX spectrum and elemental mapping images of $Cu_3(HHTP)_2$.

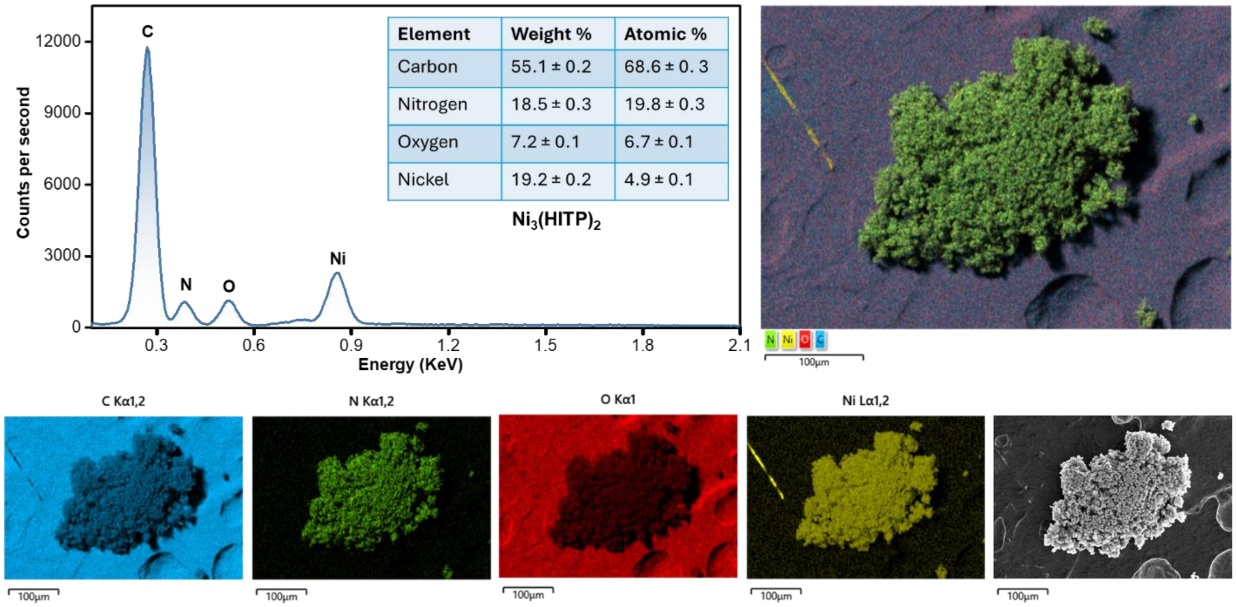

Element	Weight %	Atomic %
Carbon	55.1 ± 0.2	68.6 ± 0. 3
Nitrogen	18.5 ± 0.3	19.8 ± 0.3
Oxygen	7.2 ± 0.1	6.7 ± 0.1
Nickel	19.2 ± 0.2	4.9 ± 0.1

Figure S10. EDX spectrum and elemental mapping images of $Ni_3(HITP)_2$.

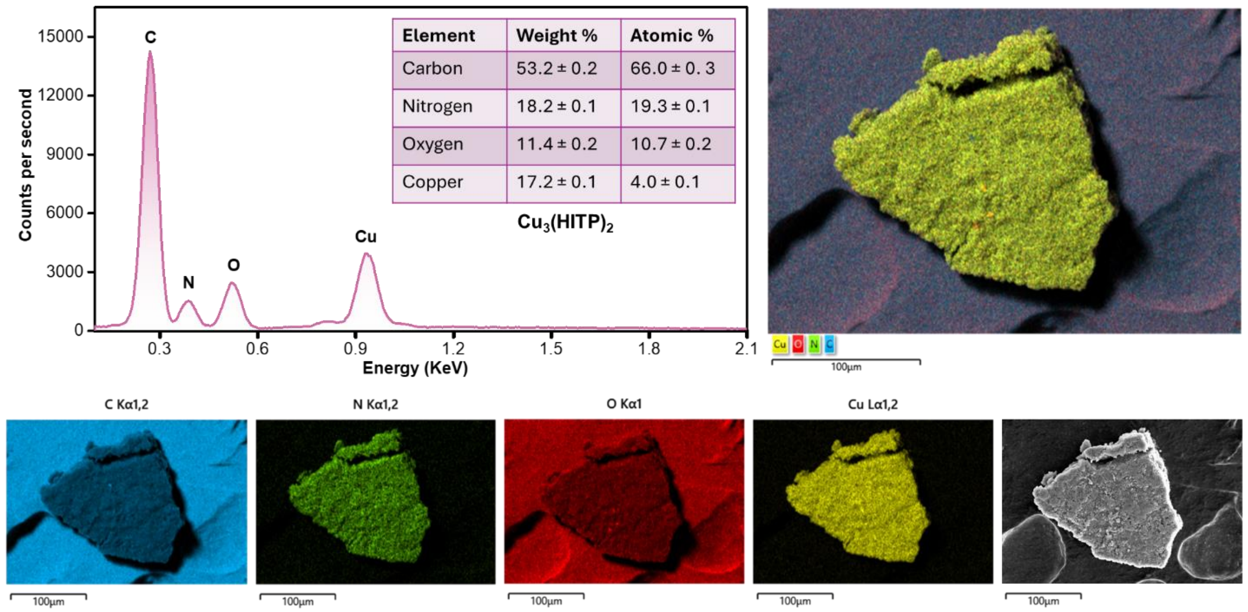

Figure S11. EDX spectrum and elemental mapping images of $Cu_3(HITP)_2$.

2.7 Characterization of the 2nd batch of MOFs

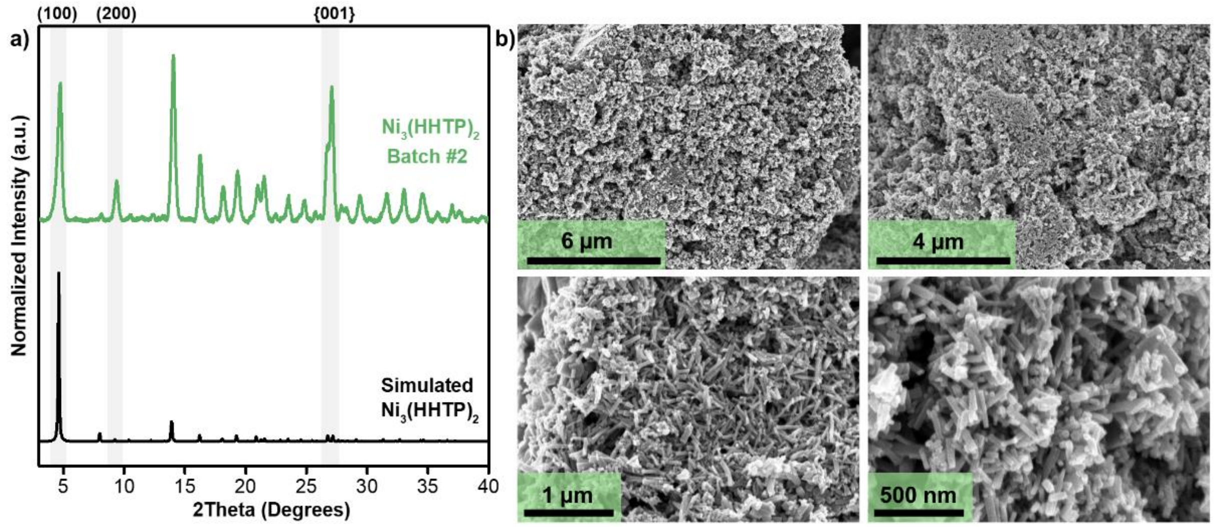

Figure S12. a) PXRD patterns and b) SEM micrographs of batch #2 of $Ni_3(HHTP)_2$.

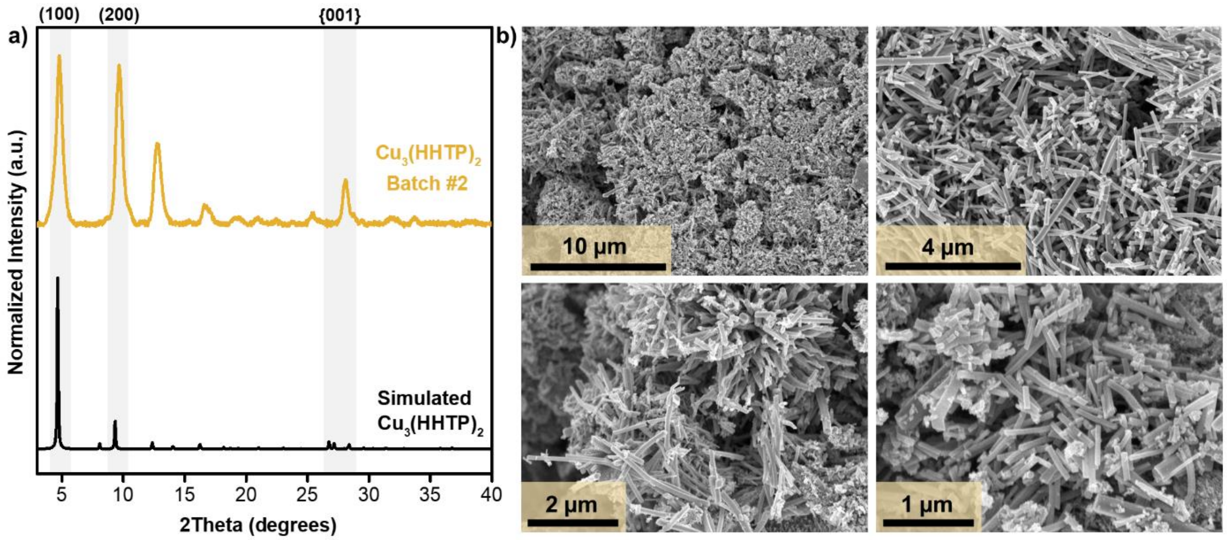

Figure S13. a) PXRD patterns and b) SEM micrographs of batch #2 of $Cu_3(HHTP)_2$.

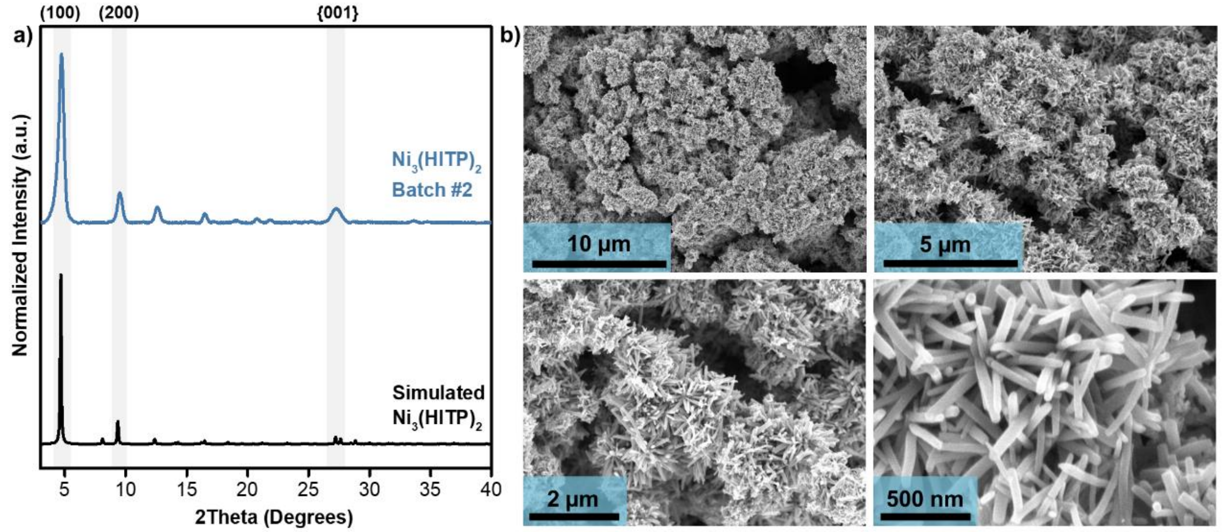

Figure S14. a) PXRD patterns and b) SEM micrographs of batch #2 of $Ni_3(HITP)_2$.

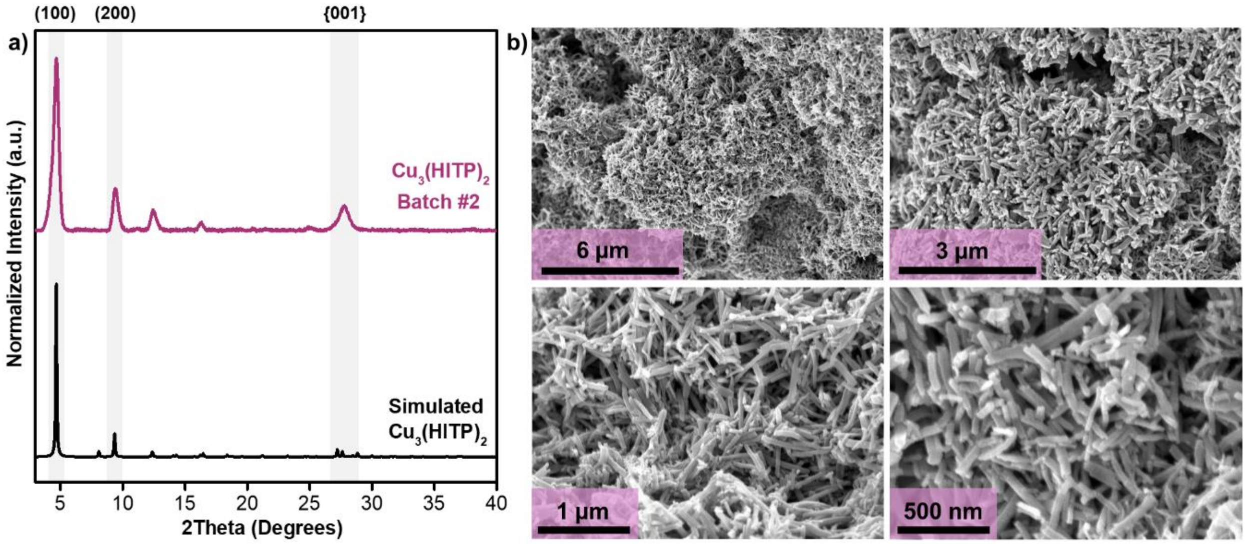

Figure S15. a) PXRD patterns and b) SEM micrographs of batch #2 of $Cu_3(HITP)_2$

2.8 Particle size analysis based on SEM micrographs

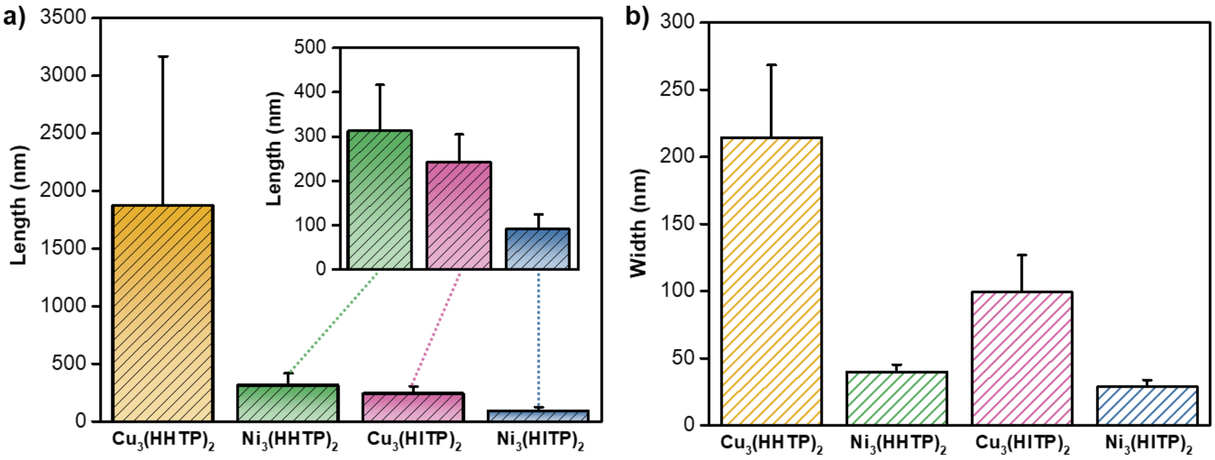

Figure S16. Average a) length and b) width of the MOF particles (Batch #1) collected from bulk syntheses of the HHTP- and HITP-based MOFs (in nm). Error bars represent the standard deviation of 70-100 particles for each MOF.

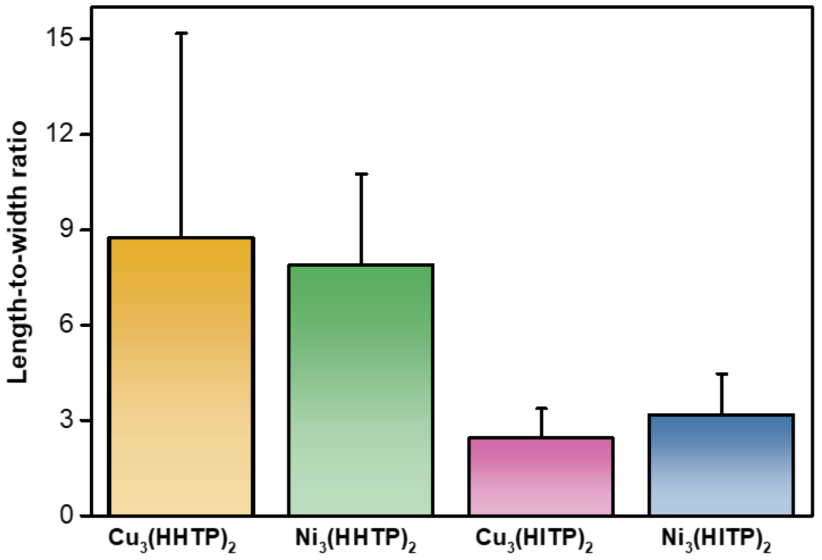

Figure S17. Average length-to-width aspect ratio (L/W) of the MOF particles (Batch #1) collected from bulk syntheses of the HHTP- and HITP-based MOFs shown in Figure S16.

2.9 Thermogravimetric analyses of MOFs

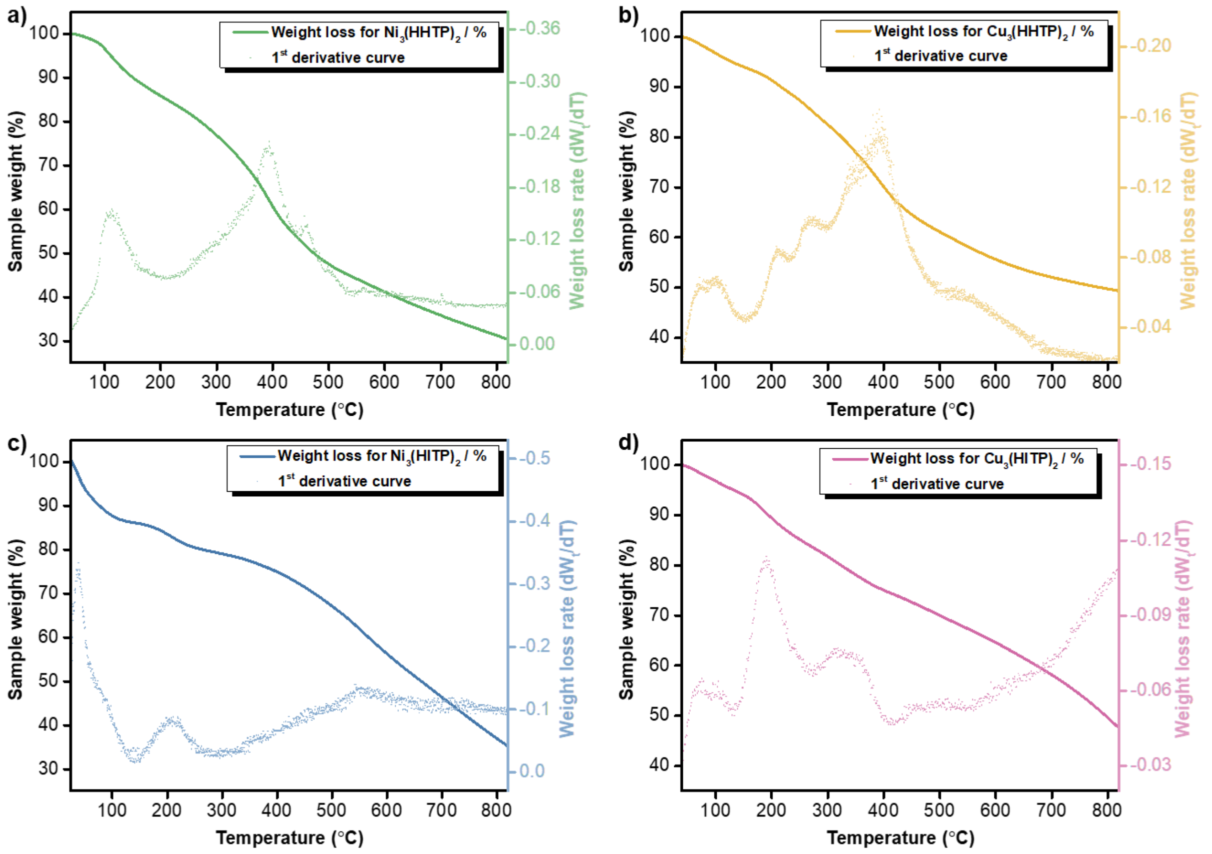

Figure S18. TGA and their first derivative curves of a) $Ni_3(HHTP)_2$, b) $Cu_3(HHTP)_2$, c) $Ni_3(HITP)_2$, and d) $Cu_3(HITP)_2$ carried out under nitrogen flow between 40 and 800 °C.

3. Adsorption experiments

3.1 Batch adsorption experiments

All adsorption experiments, unless otherwise noted, were conducted at room temperature using 8 mL glass scintillation vials on a Scilogex SCI-M-S10 10-Place Analog Magnetic Stirrer. Specifically, 2 mg of respective MOF adsorbents were added to 3 mL oxyanion solutions with concentrations ranging from 5 ppm to 500 ppm. The mixtures were stirred at room temperature for 24 hours. After this period, the MOF powders were separated from the solutions using 0.45 µm PTFE syringe filters. The supernatants were then analyzed using inductively coupled plasma mass spectrometry (ICP-MS) to quantify the remaining oxyanion concentrations.

The equilibrium adsorption capacity (Q_e), representing the amount of oxyanions adsorbed per gram of MOF material (mg of oxyanion per gram of MOF) after 24 hours of adsorption was determined using **Equation S1**:

$$Q_e(mg\ g^{-1}) = \frac{C_0 - C_e}{m} \times V \qquad \textbf{(Equation S1)}$$

Where C_0 represents the initial concentration of oxyanions (in ppm), C_e represents equilibrium concentration of the oxyanions in solution after adsorption (in ppm), *V* represents the volume of the solution (in mL), and *m* represents the mass of the MOF adsorbent (in mg).

For all adsorption experiments conducted in this study, without exception, we performed three independent tests. The error bars shown in the plots and referenced in the text represent the standard deviation of these three measurements from the calculated mean value.

3.2 Maximum adsorption capacities (Q_{max}) of MOFs

To determine the experimental Q_{max} values of all MOFs towards MnO_4^- and $Cr_2O_7^{2-}$, we carried out saturation adsorption experiments as follow: 2 mg of each MOF material was added to 20 mL of 200 ppm oxyanion solutions. The resulting solutions were left stirring at room temperature for

different time intervals after which the MOF powders were separated from the solutions using 0.45 μm PTFE syringe filters and the supernatants were analyzed by ICP-MS.

3.2.1 Maximum adsorption capacities for MnO_4^-

MOF samples	% removal at 500 ppm MnO_4^-	Experimental Q_{max} (mg g^{-1})
$Ni_3(HHTP)_2$	89 ± 6	688 ± 94
$Cu_3(HHTP)_2$	94 ± 2	820 ± 100
$Ni_3(HITP)_2$	92 ± 2	827 ± 79
$Cu_3(HITP)_2$	94 ± 1	781 ± 143

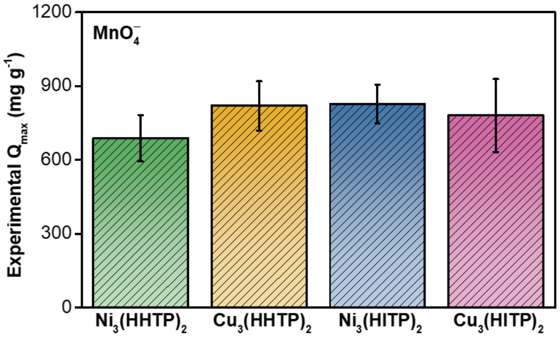

Figure S19. Experimental Q_{max} (mg g^{-1}) of the triphenylene-based MOFs towards MnO_4^- as determined via saturation adsorption experiments.

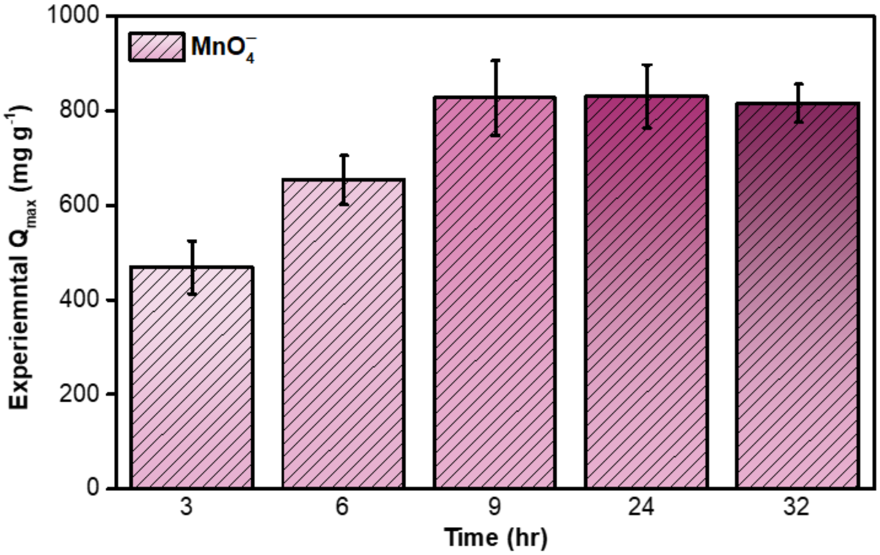

Figure S20. Experimental Q_{max} (mg g^{-1}) values of $Ni_3(HITP)_2$ for MnO_4^- at different time intervals as determined via saturation adsorption experiments.

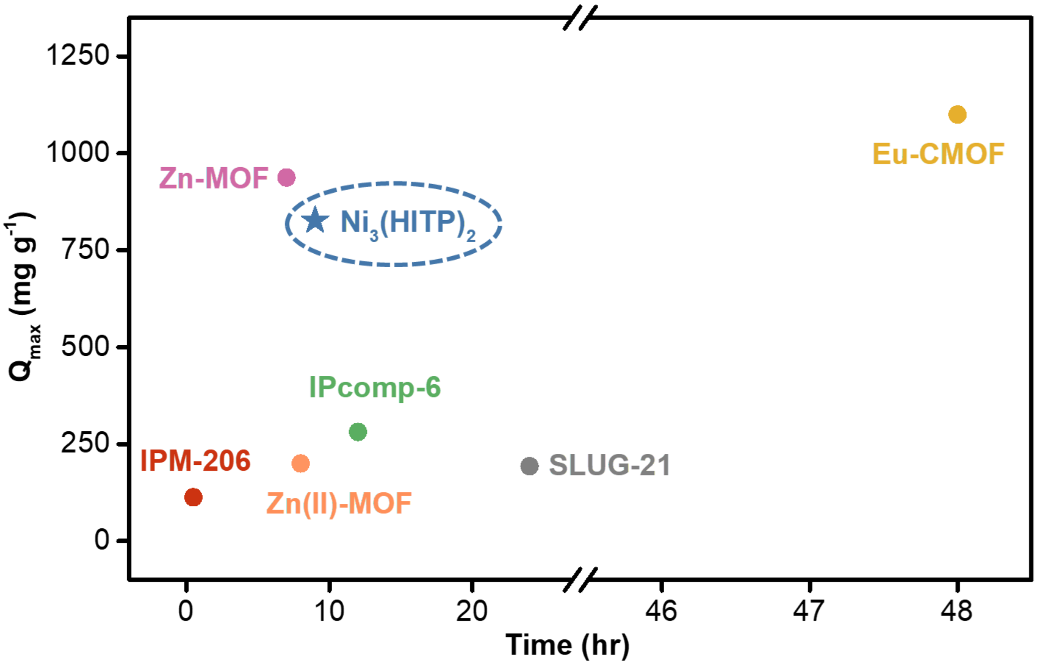

Figure S21. Comparison of the maximum adsorption uptake of MnO_4^- oxyanion from water using reported MOF-based adsorbents. For studies reporting Q_{max} values of MOF-adsorbents, please refer to the following Refs[8-13]

<u>3.2.2 Maximum adsorption capacities for $Cr_2O_7^{2-}$</u>

MOF samples	Q_e for 200 ppm $Cr_2O_7^{2-}$	Experimental Q_{max} (mg g^{-1})
$Ni_3(HHTP)_2$	89 ± 18	225 ± 26
$Cu_3(HHTP)_2$	65 ± 8	132 ± 22
$Ni_3(HITP)_2$	195 ± 23	497 ± 11
$Cu_3(HITP)_2$	158 ± 7	237 ± 56

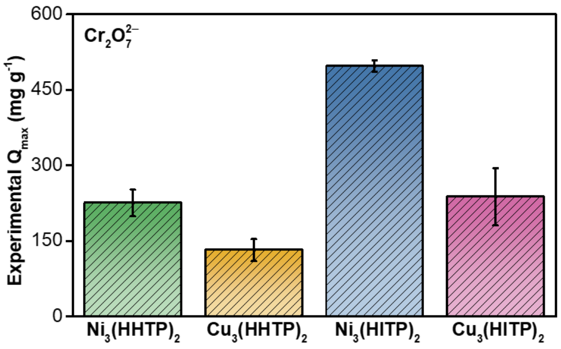

Figure S22. Experimental Q_{max} (mg g^{-1}) of the triphenylene-based MOFs towards $Cr_2O_7^{2-}$ oxyanion as determined via saturation adsorption experiments.

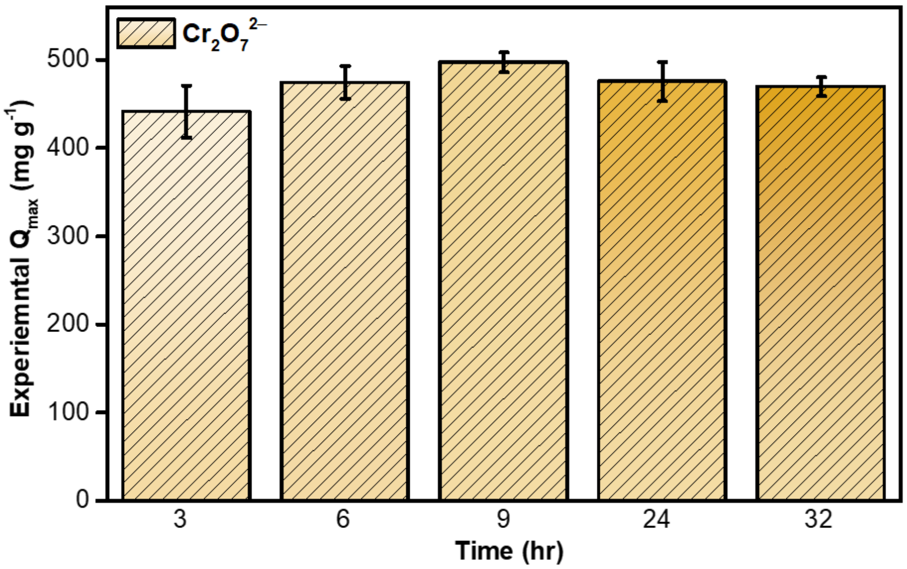

Figure S23. Experimental Q_{max} (mg g^{-1}) values of $Ni_3(HITP)_2$ for $Cr_2O_7^{2-}$ at different time intervals as determined via saturation adsorption experiments.

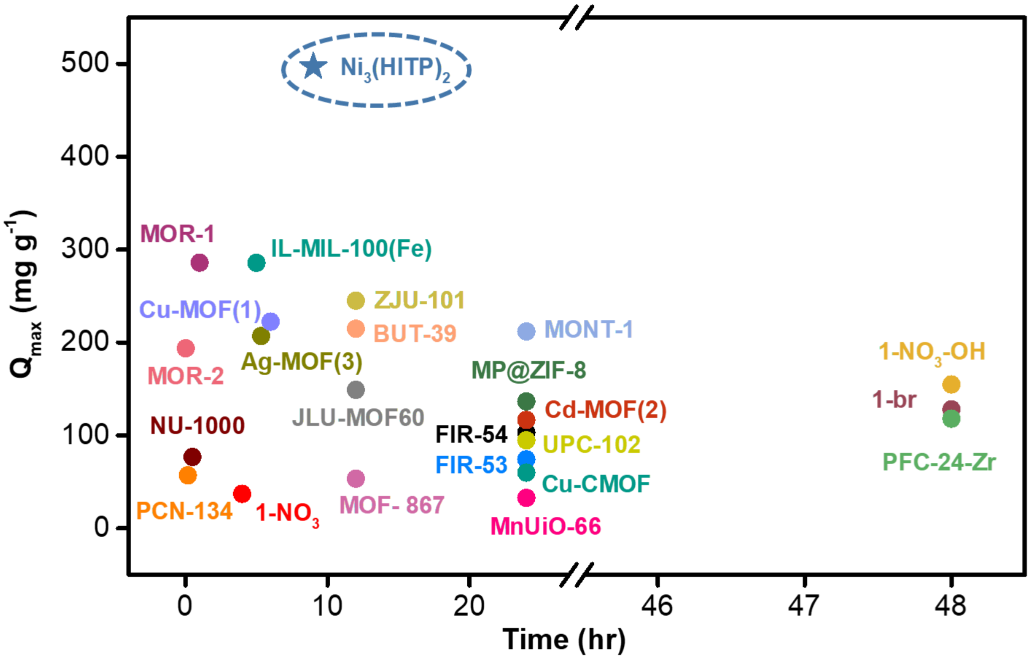

Figure S24. Comparison of the maximum adsorption uptake of $Cr_2O_7^{2-}$ oxyanion from water using reported MOF-based adsorbents. For studies reporting Q_{max} values of MOF-adsorbents, please refer to the following Refs[14-35]

3.3 Surface charge of MOFs

3.3.1 Dye adsorption experiments

We determined the surface charge of the MOFs in aqueous solution through dye adsorption experiments. Briefly, we dispersed 2 mg of MOF powder in 3 mL of a 20 ppm aqueous solution of either the cationic dye methylene blue (MB^+) or the anionic dye methyl orange (MO^-). The mixtures were stirred at room temperature for 2 hours after which we separated the MOF powder using 0.45 μm syringe filters. Finally, we analyzed the supernatants via UV-Vis spectroscopy to evaluate the dye adsorption capacities of the MOFs.

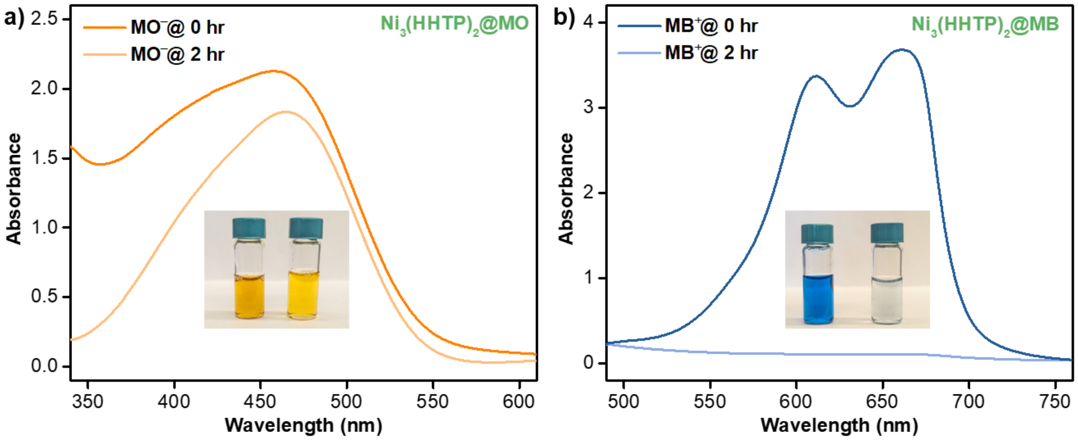

Figure S25. UV-vis absorption spectra of a) MO^- and b) MB^+ dye solutions before and after adsorption experiments in DI water using $Ni_3(HHTP)_2$. Inset: Photographs of the aqueous solutions before and after dye adsorption.

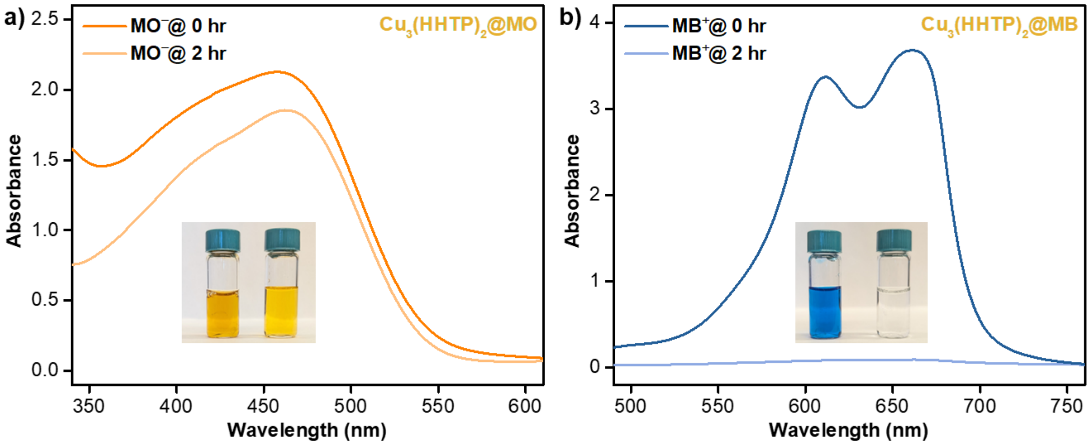

Figure S26. UV-vis absorption spectra of a) MO^- and b) MB^+ dye solutions before and after adsorption experiments in DI water using $Cu_3(HHTP)_2$. Inset: Photographs of the aqueous solutions before and after dye adsorption.

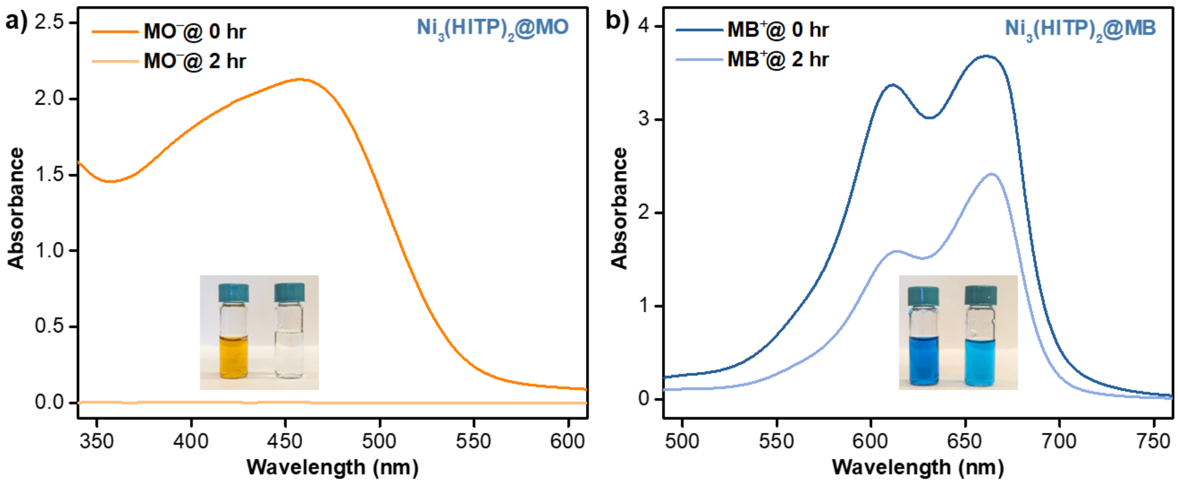

Figure S27. UV-vis absorption spectra of a) MO^- and b) MB^+ dye solutions before and after adsorption experiments in DI water using $Ni_3(HITP)_2$. Inset: Photographs of the aqueous solutions before and after dye adsorption.

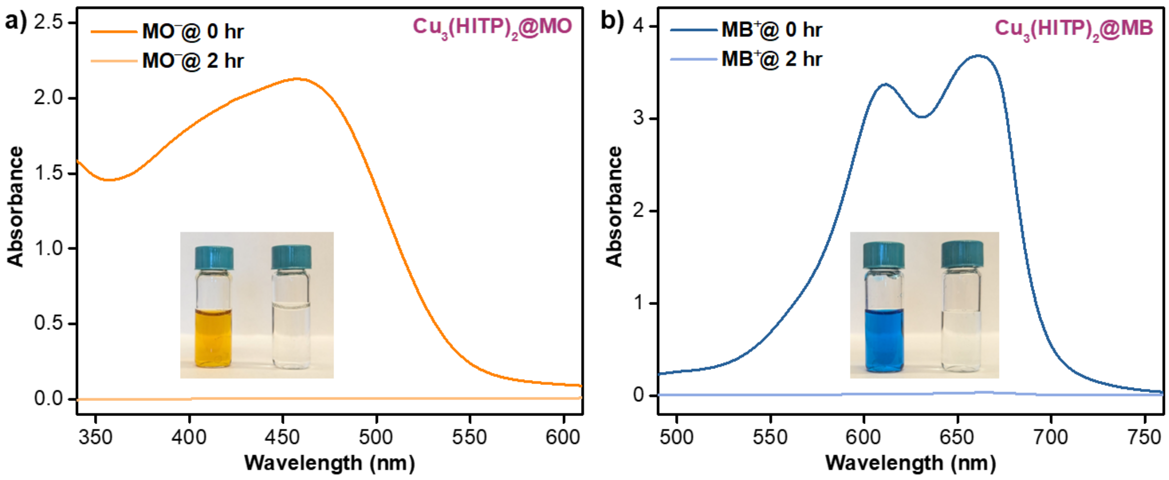

Figure S28. UV-vis absorption spectra of a) MO^- and b) MB^+ dye solutions before and after adsorption experiments in DI water using $Cu_3(HITP)_2$. Inset: Photographs of the aqueous solutions before and after dye adsorption.

3.3.2 Zeta potential measurements

Zeta potential measurements were carried out on a Malvern ZetaSizer Nano. In brief, 1 mg of MOF powders were homogeneously dispersed in 1 mL of Milli-Q water by sonication overnight

(12 hours). The resulting suspension was transferred into a Malvern panalytical folded capillary zeta cell and the Zeta potential values were recorded at room temperature.

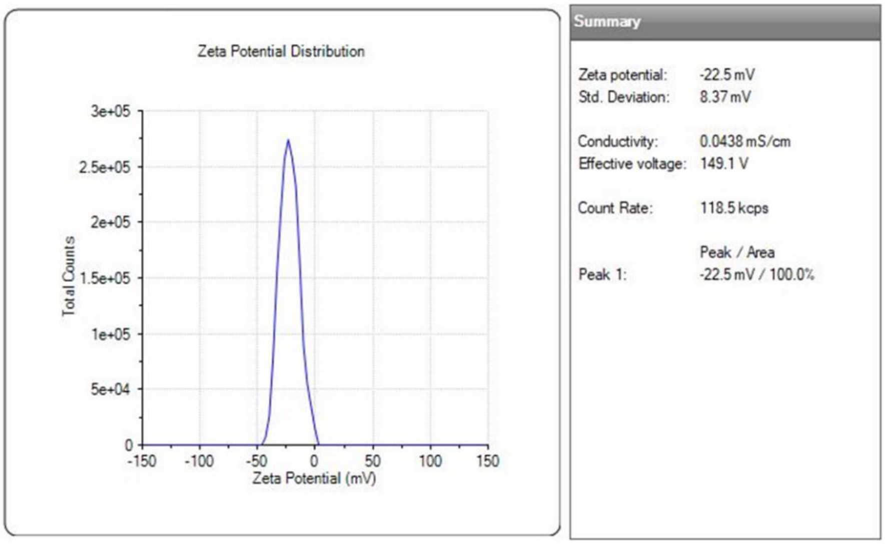

Figure S29. Zeta potential measurement of $Ni_3(HHTP)_2$ in water (1 mg mL^{-1}).

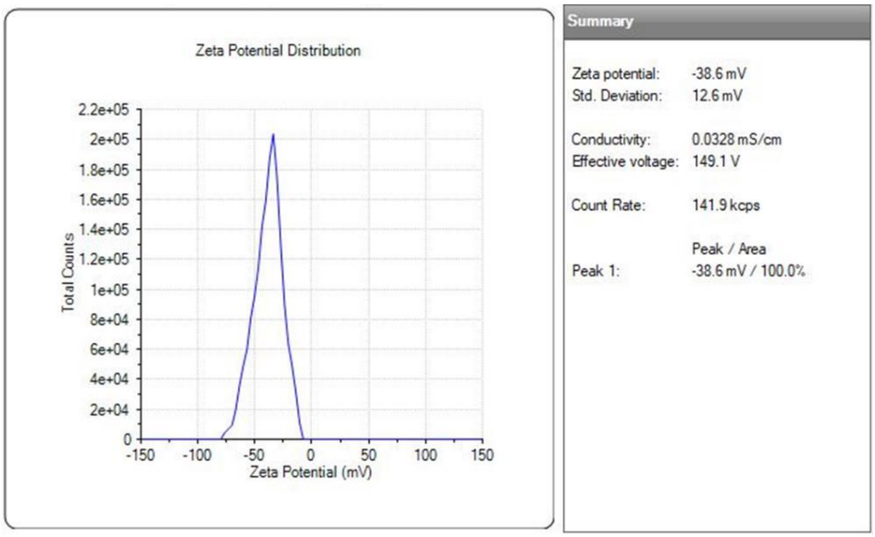

Figure S30. Zeta potential measurement of $Cu_3(HHTP)_2$ in water (1 mg mL^{-1}).

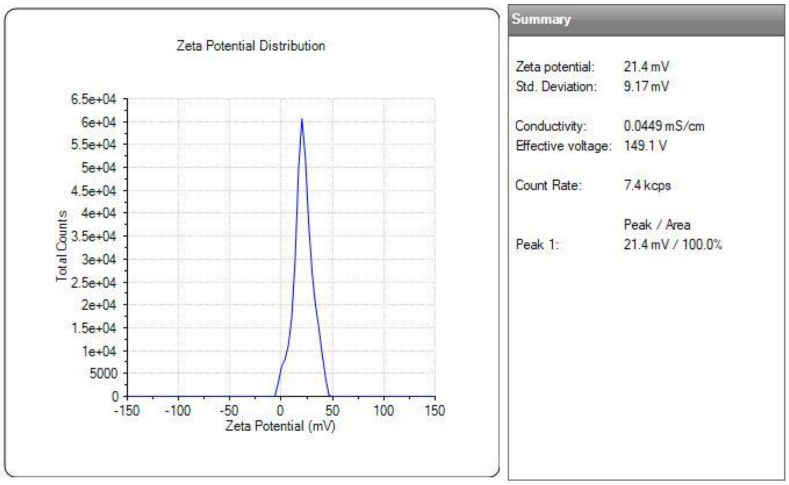

Figure S31. Zeta potential measurement of $Ni_3(HITP)_2$ in water (1 mg mL^{-1}).

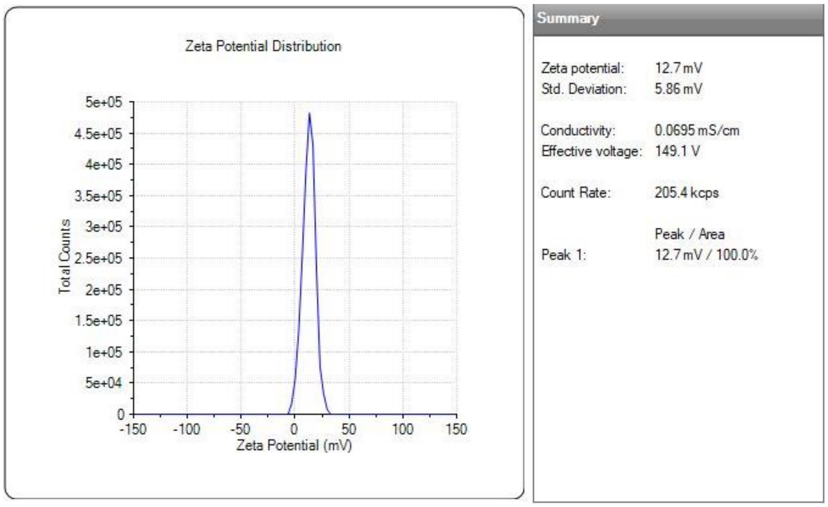

Figure S32. Zeta potential measurement of $Cu_3(HITP)_2$ in water (1 mg mL^{-1}).

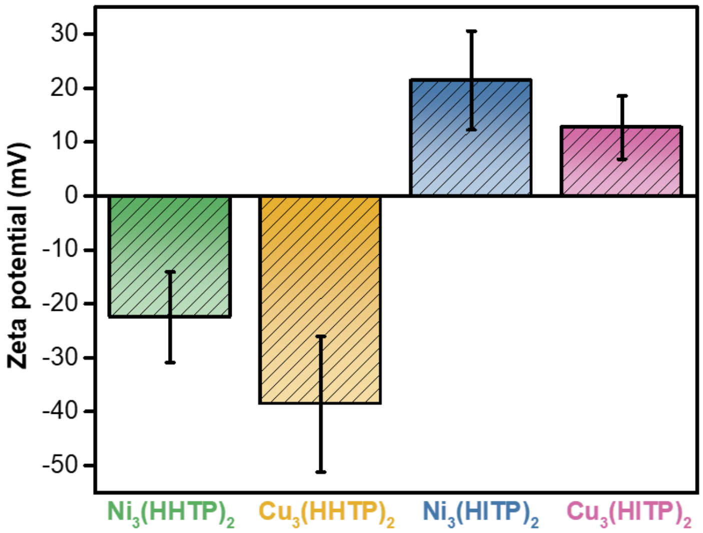

Figure S33. Comparison of Zeta potential values of the HHTP- and HITP-based MOFs (1 mg mL^{-1}) used in this study.

3.4 Adsorption kinetic studies

Kinetic studies were conducted to evaluate the adsorption rate and removal efficiency of the MOF adsorbents toward oxyanions over time. In these experiments, multiple vials were prepared, each containing 2 mg of MOF dispersed in 3 mL of a 25 ppm oxyanion solution. The vials were continuously stirred, and at designated time intervals ranging from 1 to 60 minutes, individual vials were removed for analysis. The supernatants from these vials were collected at 1, 2, 3, 5, 7, 10, 15, 30, and 60 minutes and analyzed using ICP-MS to determine the uptake capacity of each MOF at different time points.

The time-dependent uptake capacity, Q_t, representing the amount of oxyanion adsorbed per gram of MOF material (mg of oxyanion per gram of MOF) at a given time *t*, was calculated using **Equation S2**:

$$Q_t(mg\ g^{-1}) = \frac{C_0 - C_t}{m} \times V \qquad \textbf{(Equation S2)}$$

where C_0 is the initial oxyanion concentration (25 ppm), C_t is the concentration of oxyanion remaining in solution at time t (ppm), V is the volume of solution (mL), and m is the mass of the MOF adsorbent (mg).

To predict the mode of interactions between the oxyanions and MOF adsorbents, the experimental data were fitted with two theoretical kinetic models, mainly the pseudo-first order (**Equation S3**) and pseudo-second order (**Equation S4**) models. The pseudo-first order kinetic model assumes the adsorption process to be dominated by external and/or internal diffusion processes rather than chemical interactions between the MOF adsorbents and oxyanions.[36] A good fit of the experimental adsorption data, based on the correlation coefficient (R^2) value indicates a predominant physical adsorption process involving non-covalent interactions, such as Van der Waals, hydrogen bonds, and π-π interactions. Conversely, the pseudo-second order kinetic model assumes the adsorption process of oxyanion onto the active sites of the MOF adsorbents to be the main mechanism and rate-controlling step of the adsorption mechanism.[36] A good fit of the experimental adsorption data, based on R^2 value indicates a predominant chemical adsorption process involving covalent interactions.

$$\log(Q_e - Q_t) = \log Q_e - (\frac{K_1}{2.303})t \qquad \textbf{(Equation S3)}$$

$$\frac{t}{Q_t} = \frac{t}{Q_e} + \frac{1}{K_2 Q_e^2} \qquad \textbf{(Equation S4)}$$

Q_e represents the maximum uptake capacity of the MOF at the studied concentration (in mg g^{-1}), Q_t represents the uptake capacity at time t (in mg g^{-1}), K_1 is the pseudo-first order rate constant (in min^{-1}), K_2 is the pseudo-second-order rate constant (g mg^{-1} min^{-1}), and t is the adsorption time (in min).

3.4.1 Kinetic isotherms for MnO_4^-

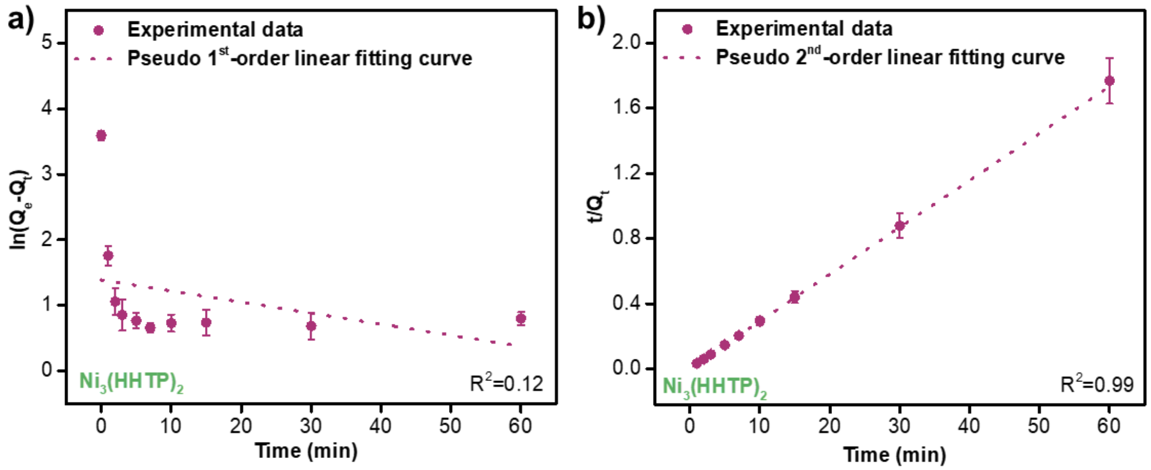

Figure S34. Kinetic data fitting of MnO_4^- oxyanion via the a) pseudo-first and b) pseudo-second order models for $Ni_3(HHTP)_2$ samples. Conditions: m_{MOF} = 2 mg, $V_{solution}$ = 3 mL, and T = 298 K at different time intervals. Error bars represent standard deviation from the mean value of three independent kinetic experiments.

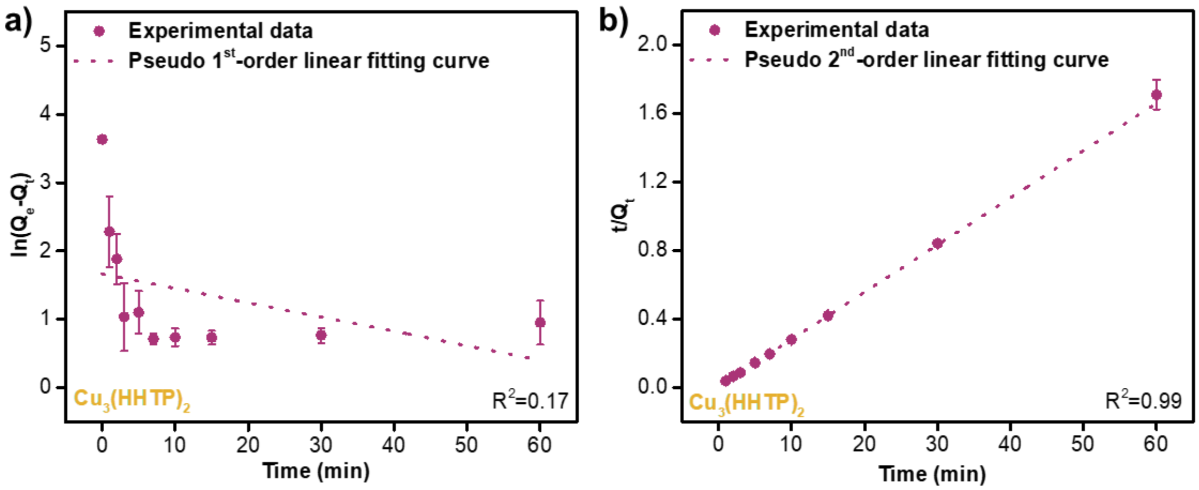

Figure S35. Kinetic data fitting of MnO_4^- oxyanion via the a) pseudo-first and b) pseudo-second order models for $Cu_3(HHTP)_2$ samples. Conditions: m_{MOF} = 2 mg, $V_{solution}$ = 3 mL, and T = 298 K at different time intervals. Error bars represent standard deviation from the mean value of three independent kinetic experiments.

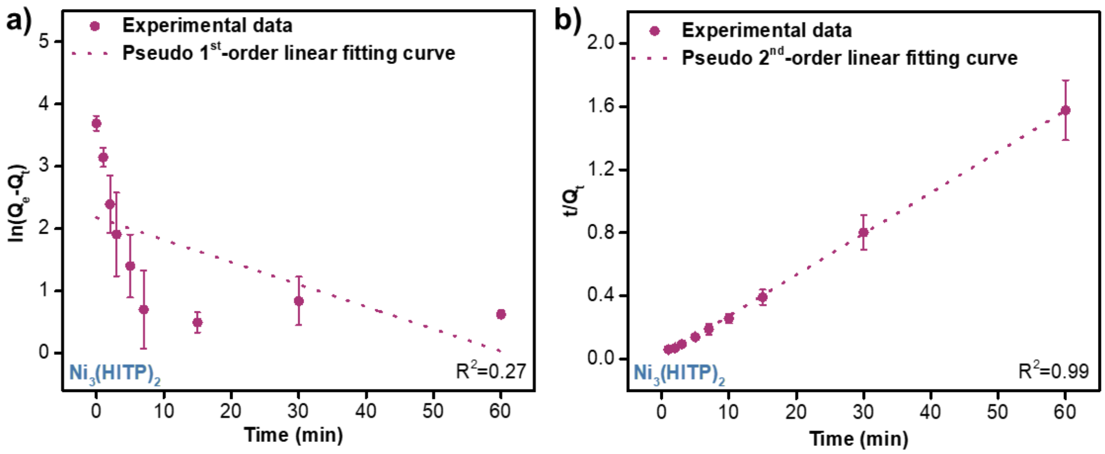

Figure S36. Kinetic data fitting of MnO_4^- oxyanion via the a) pseudo-first and b) pseudo-second order models for $Ni_3(HITP)_2$ samples. Conditions: m_{MOF} = 2 mg, $V_{solution}$ = 3 mL, and T = 298 K at different time intervals. Error bars represent standard deviation from the mean value of three independent kinetic experiments.

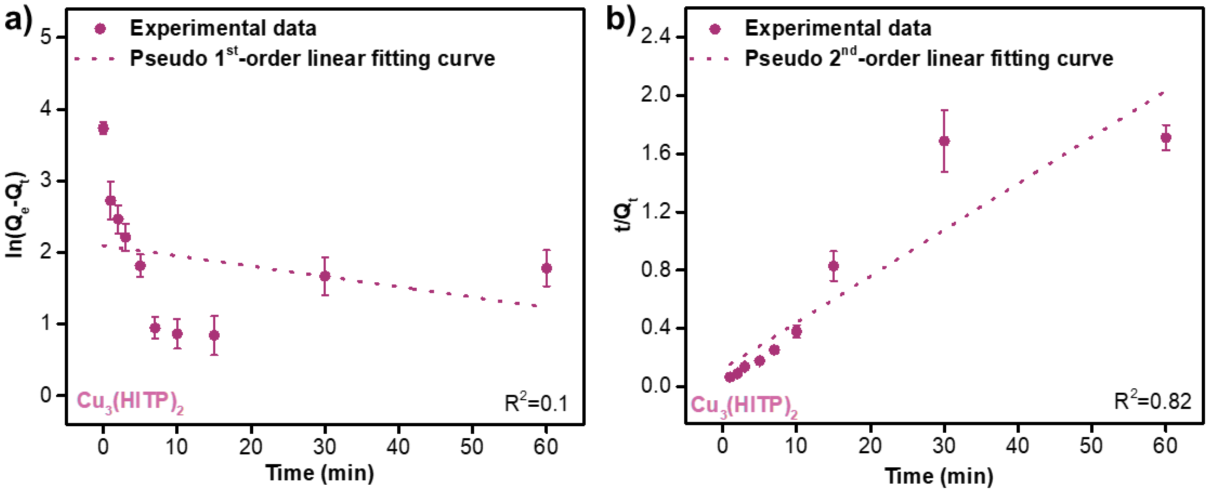

Figure S37. Kinetic data fitting of MnO_4^- oxyanion via the a) pseudo-first and b) pseudo-second order models for $Cu_3(HITP)_2$ samples. Conditions: m_{MOF} = 2 mg, $V_{solution}$ = 3 mL, and T = 298 K at different time intervals. Error bars represent standard deviation from the mean value of three independent kinetic experiments.

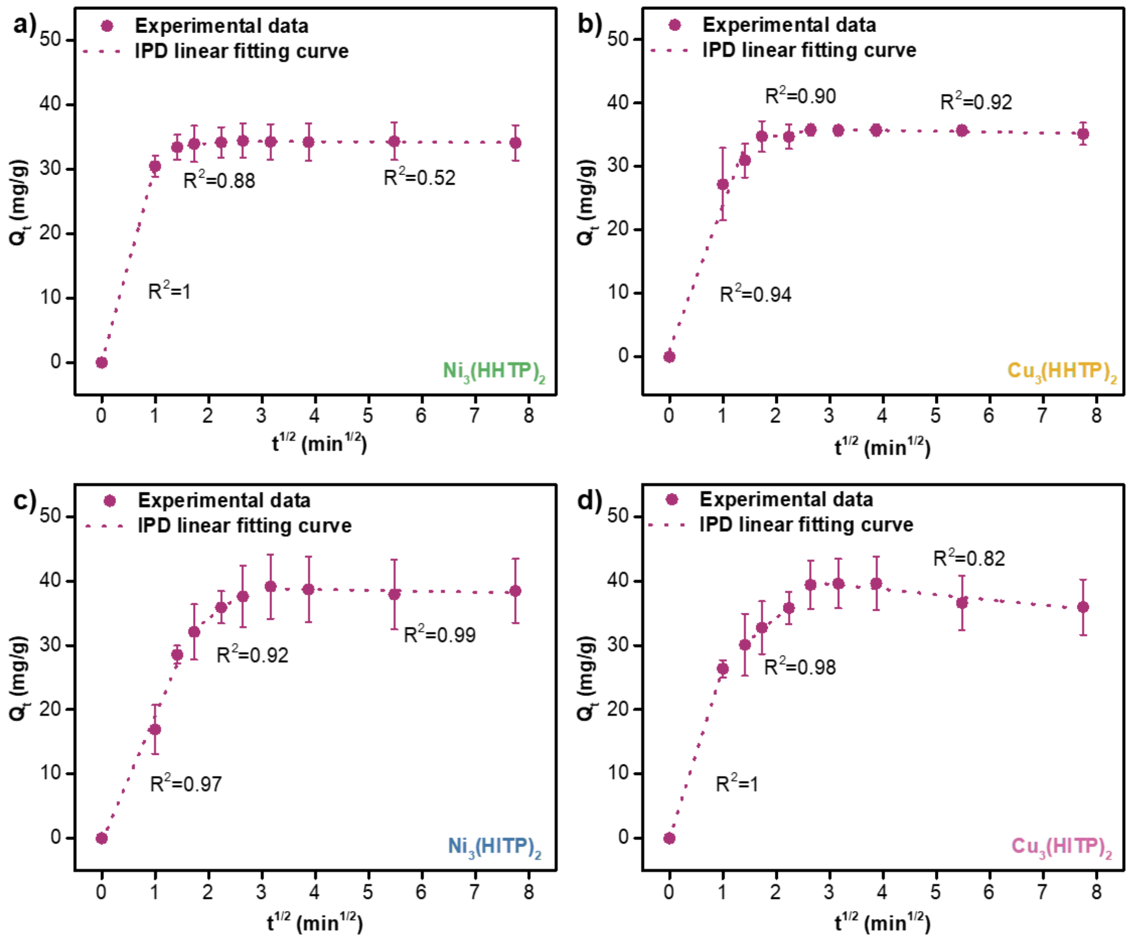

Figure S38. Intra-particle diffusion model of MnO_4^- oxyanion on a) $Ni_3(HHTP)_2$, b) $Cu_3(HHTP)_2$, c) $Ni_3(HITP)_2$, and d) $Cu_3(HITP)_2$ MOFs.

3.4.2 Kinetic isotherms for $Cr_2O_7^{2-}$

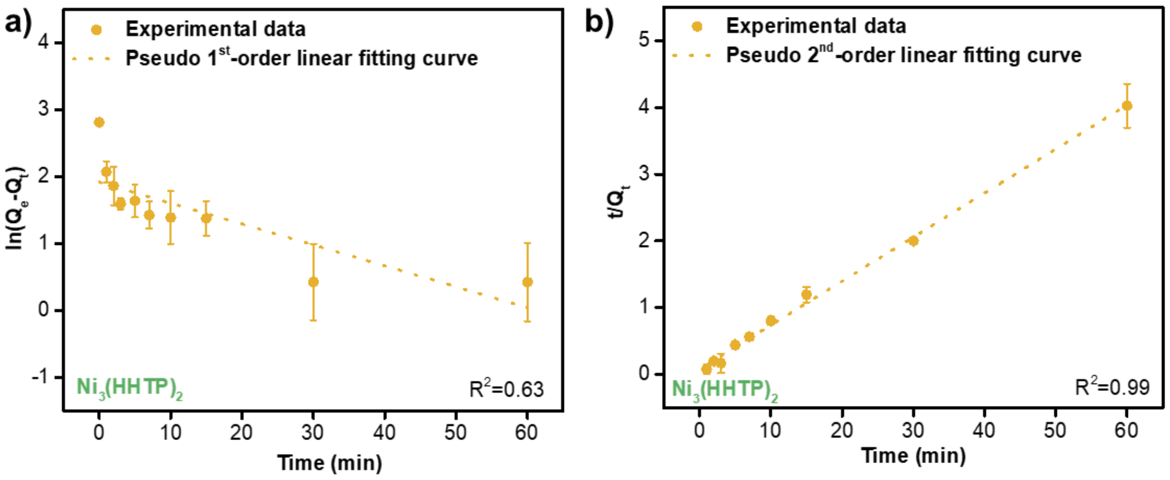

Figure S39. Kinetic data fitting of $Cr_2O_7^{2-}$ oxyanion via the a) pseudo-first and b) pseudo-second order models for $Ni_3(HHTP)_2$ samples. Conditions: m_{MOF} = 2 mg, $V_{solution}$ = 3 mL, and T = 298 K at different time intervals. Error bars represent standard deviation from the mean value of three independent kinetic experiments.

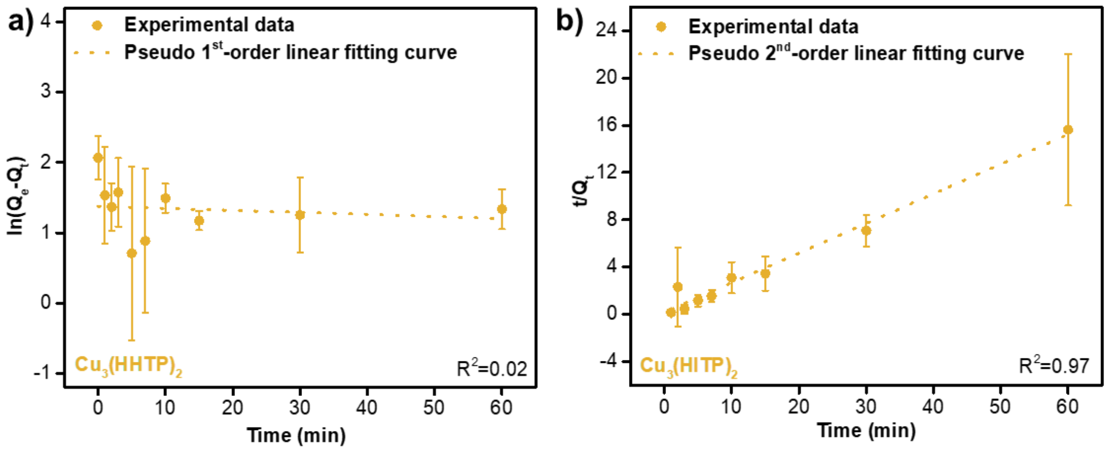

Figure S40. Kinetic data fitting of $Cr_2O_7^{2-}$ oxyanion via the a) pseudo-first and b) pseudo-second order models for $Cu_3(HHTP)_2$ samples. Conditions: m_{MOF} = 2 mg, $V_{solution}$ = 3 mL, and T = 298 K at different time intervals. Error bars represent standard deviation from the mean value of three independent kinetic experiments.

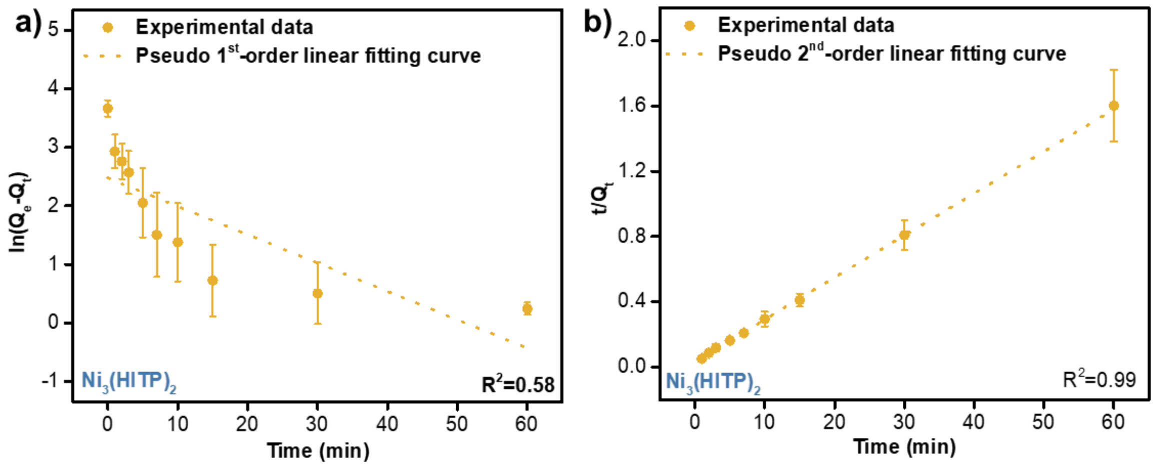

Figure S41. Kinetic data fitting of $Cr_2O_7^{2-}$ oxyanion via the a) pseudo-first and b) pseudo-second order models for $Ni_3(HITP)_2$ samples. Conditions: m_{MOF} = 2 mg, $V_{solution}$ = 3 mL, and T = 298 K at different time intervals. Error bars represent standard deviation from the mean value of three independent kinetic experiments.

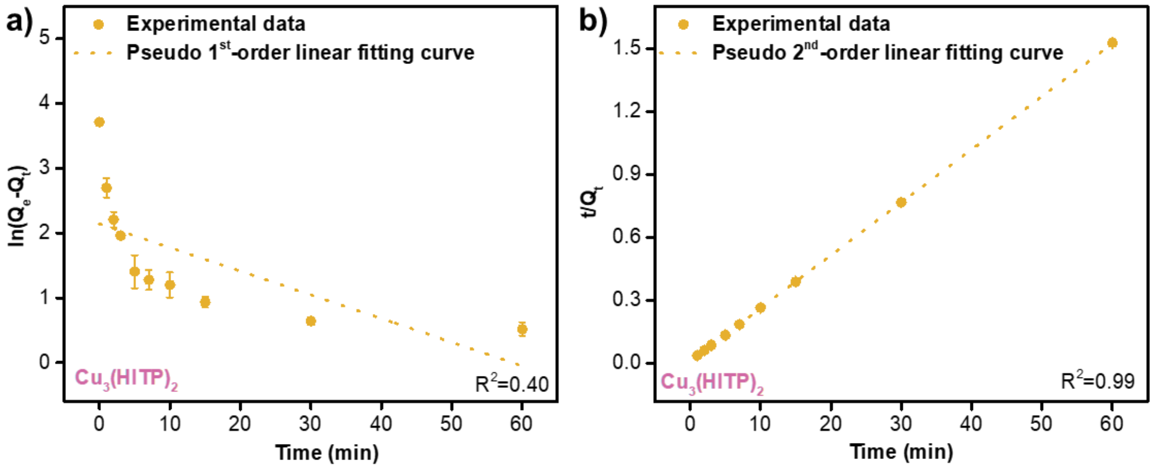

Figure S42. Kinetic data fitting of $Cr_2O_7^{2-}$ oxyanion via the a) pseudo-first and b) pseudo-second order models for $Cu_3(HITP)_2$ samples. Conditions: m_{MOF} = 2 mg, $V_{solution}$ = 3 mL, and T = 298 K at different time intervals. Error bars represent standard deviation from the mean value of three independent kinetic experiments.

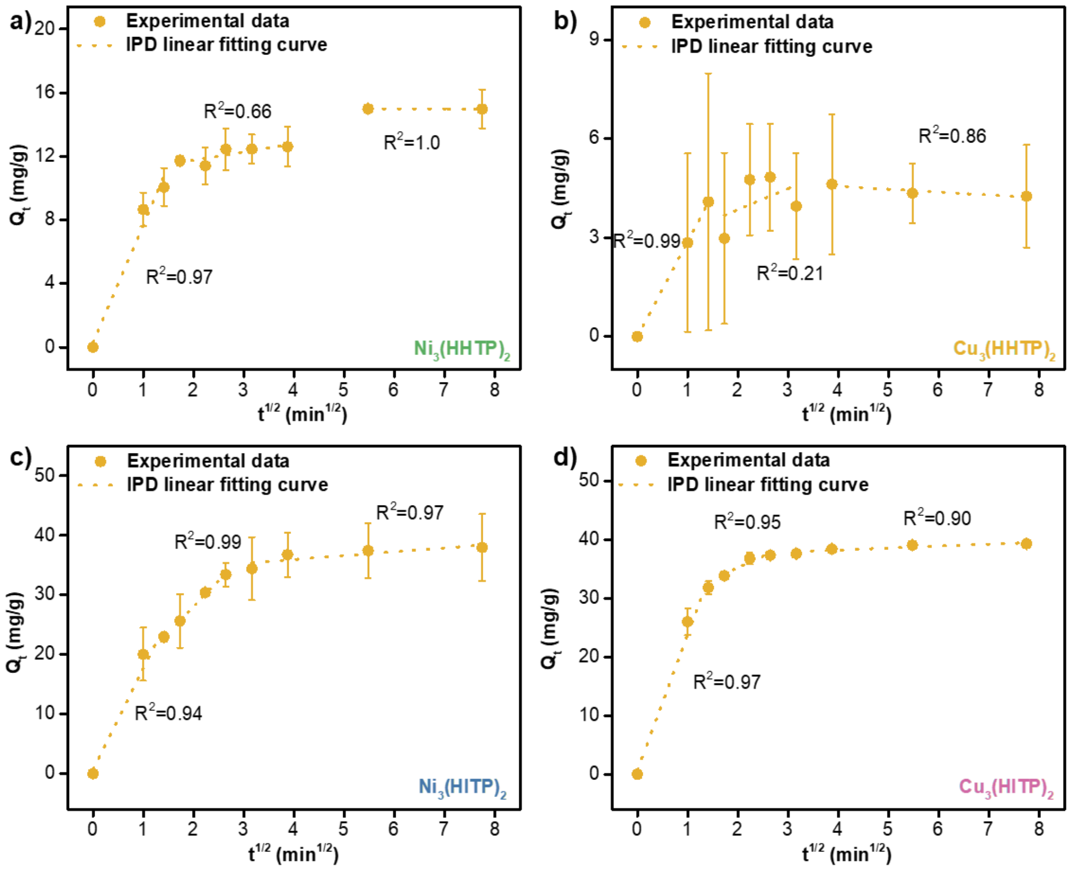

Figure S43. Intra-particle diffusion model of $Cr_2O_7^{2-}$ oxyanion on a) $Ni_3(HHTP)_2$, b) $Cu_3(HHTP)_2$, c) $Ni_3(HITP)_2$, and d) $Cu_3(HITP)_2$ MOFs.

3.5 Oxyanion removal under diverse aquatic environments

3.5.1 Effect of co-existing anions

The effect of co-existing anions on the removal efficiency of $Ni_3(HITP)_2$ was investigated by preparing 25 ppm oxyanion solutions co-existing with equimolar amounts of 1) NaCl, 2) Na_2CO_3, 3) Na_3PO_4, 4) CH_3COONa, 5) $NaNO_3$, and 6) Na_2SO_4. 2 mg of $Ni_3(HITP)_2$ was added to each solution (3 mL) and the resulting mixture was allowed to stir for 24 hours at room temperature. The MOF powders were separated from the solutions using 0.45 µm PTFE syringe filters and the supernatants were analyzed by ICP-MS. Results for these experiments are shown in **Figure 3b**.

3.5.2 Effect of pH

Adsorption experiments were carried out following procedure detained in **Section S3.1** with the exception that the initial concentration of oxyanions was set to 100 ppm. The pH of the solutions was adjusted by adding HCl and NaOH.

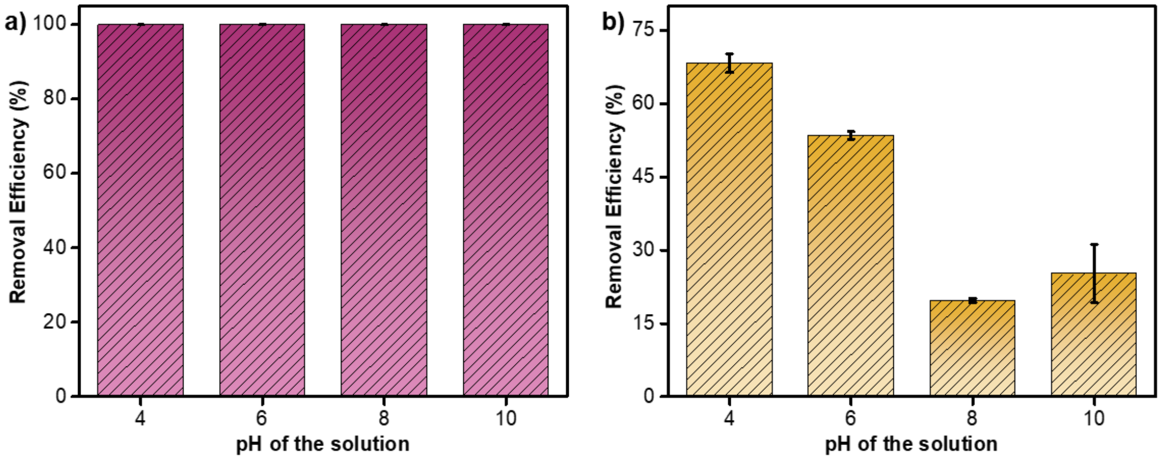

Figure S44. Removal efficiency of $Ni_3(HITP)_2$ towards a) Mn(VII) and b) Cr(VI) oxyanions at different pH ranges. Conditions: m_{MOF} = 2 mg, $V_{solution}$ = 3 mL, [oxyanion] = 100 ppm, and t = 24 hours.

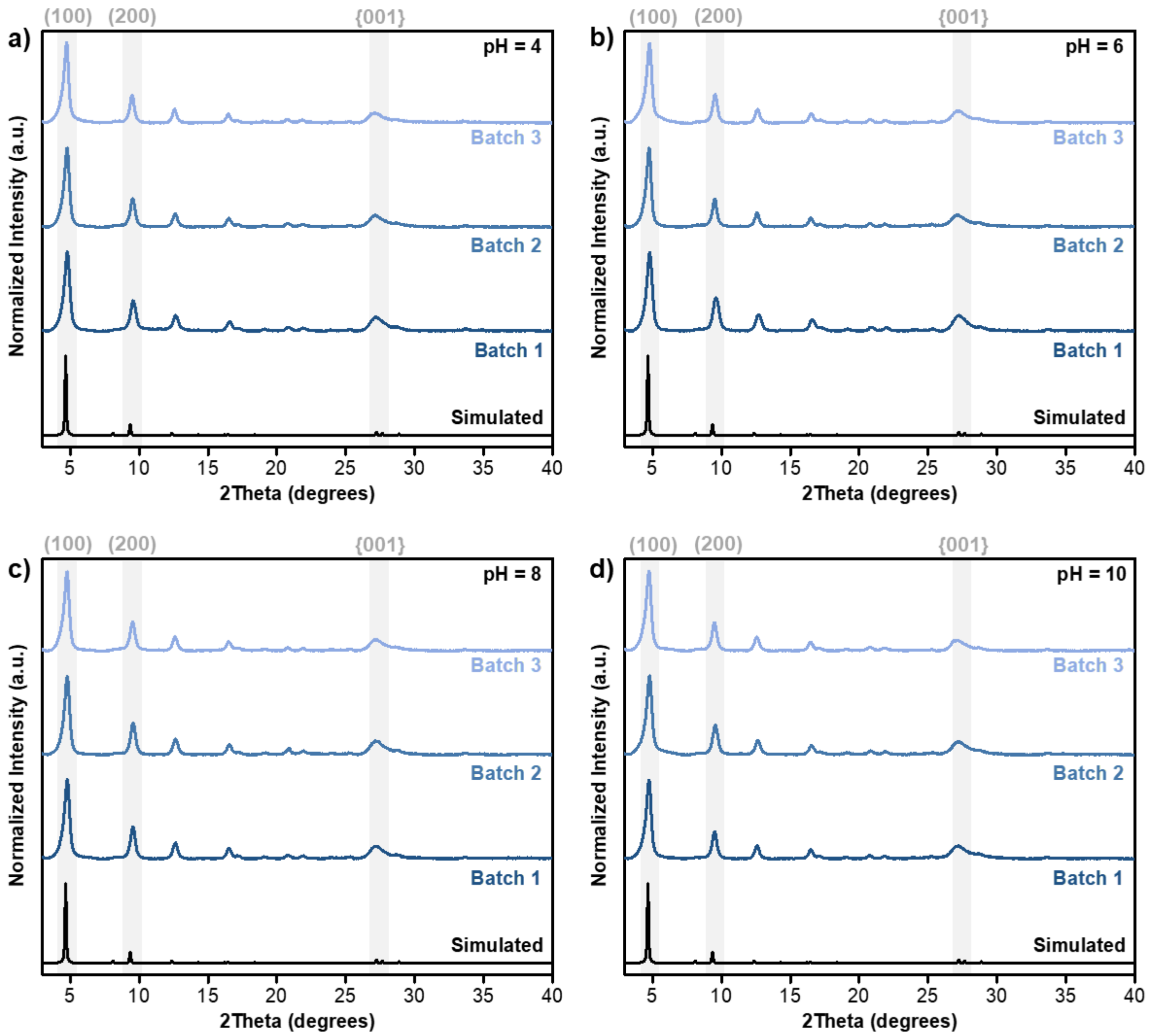

Figure S45. PXRD patterns of $Ni_3(HITP)_2$ MOFs after soaking for 24 hours in aqueous solutions with pH values of a) 4, b) 6, c) 8, and d) 10.

3.5.3 Selectivity towards different manganese and chromium species

We evaluated the selectivity of $Ni_3(HITP)_2$ towards manganese and chromium ions with distinct oxidation states, sizes and charges. Adsorption experiments were carried out using the same procedure described in **Section S3.1** at three different concentrations (20, 100, and 200 ppm).

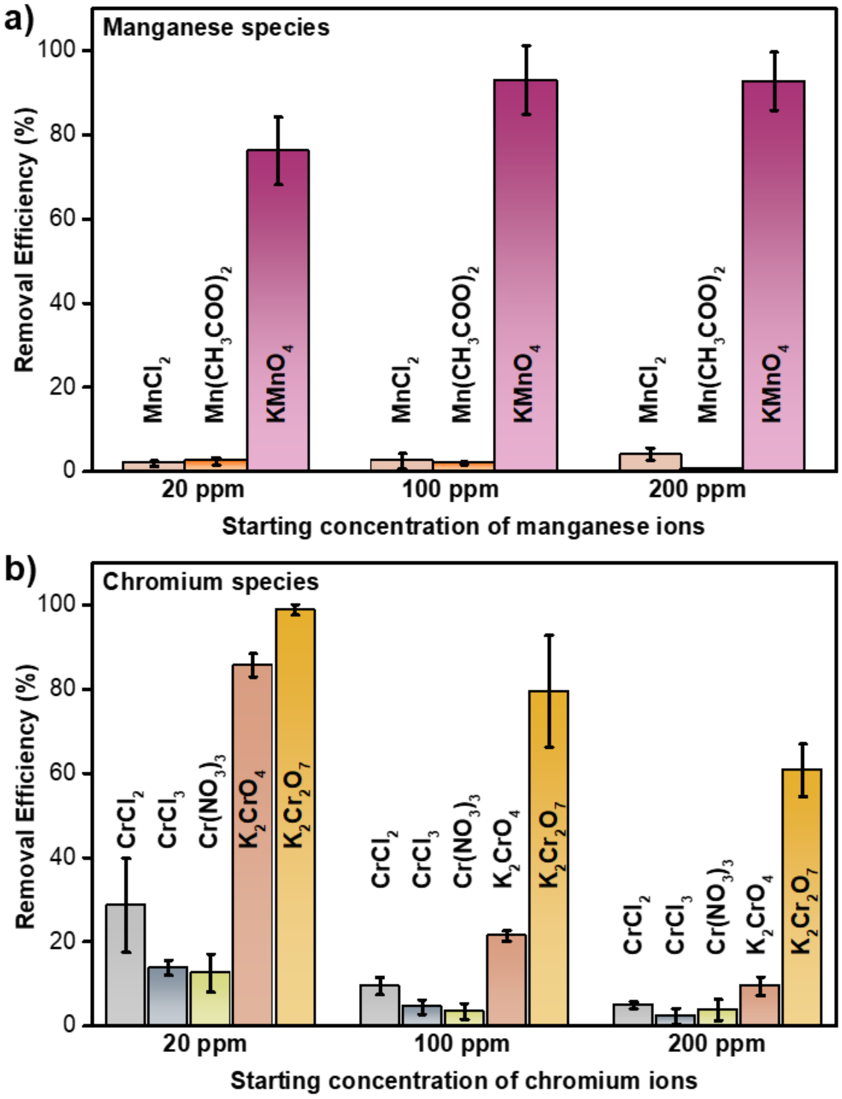

Figure S46. Removal efficiency of $Ni_3(HITP)_2$ towards different concentrations of a) manganese and b) chromium species possessing distinct charges and oxidation states.

3.5.4 Recyclability of $Ni_3(HITP)_2$

The reusability of $Ni_3(HITP)_2$ MOF was investigated over eights cycles. Following each oxyanion adsorption cycle, the MOF powder was separated from solutions by centrifugation, washed with DI water (5 mL) and ethanol (5 mL) two times, and finally soaked in 1 M aqueous HCl solution overnight. The MOF powder was then washed with DI water (10 mL) and dried in vacuum oven set at 72 °C before being used for the next cycle.

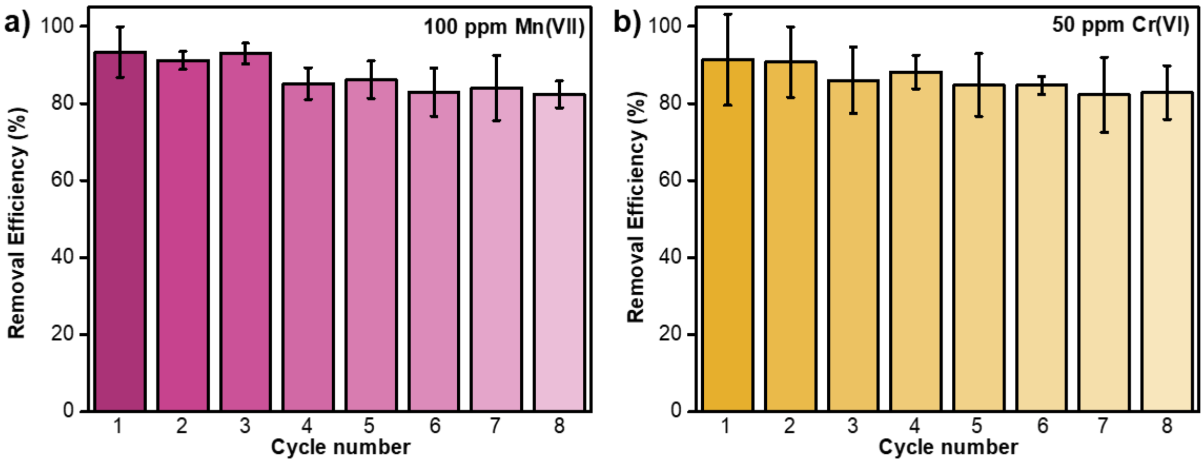

Figure S47. Regeneration performance of $Ni_3(HITP)_2$ towards a) 100 ppm MnO_4^- and b) and 50 ppm $Cr_2O_7^{2-}$ solutions.

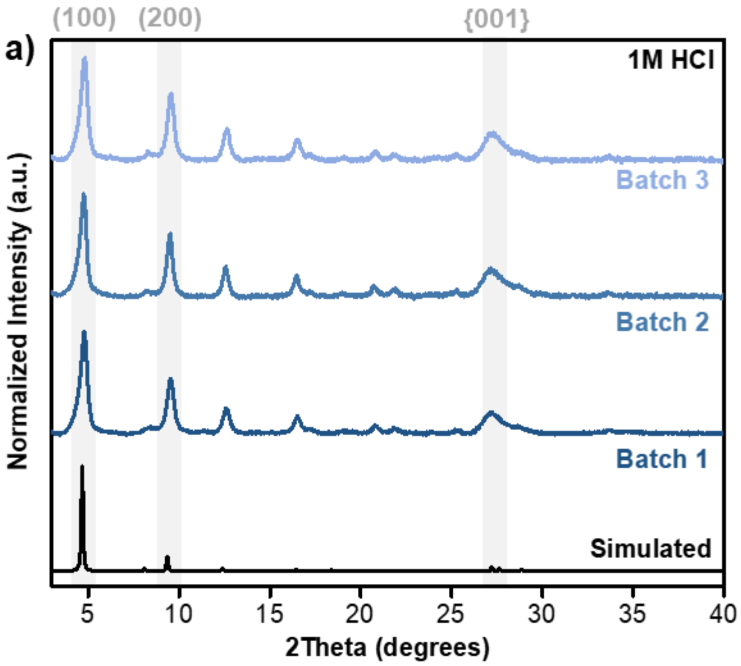

Figure S48. PXRD patterns of $Ni_3(HITP)_2$ MOFs after soaking for 24 hours in 1M HCl solution.

4. Mechanistic insights into the MOF-oxyanion interactions

4.1 ATR-FTIR spectroscopy

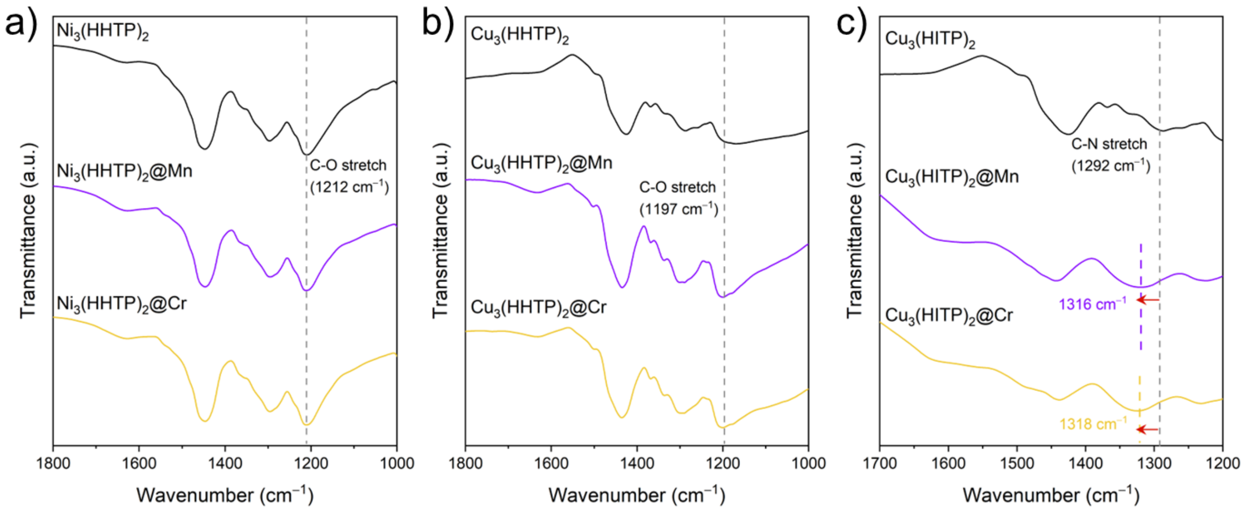

Figure S49. FT-IR spectra of pristine (black line), after MnO_4^- oxyanion (purple line) and $Cr_2O_7^{2-}$ oxyanion (yellow line) uptake for (a) $Ni_3(HHTP)_2$, (b) $Cu_3(HHTP)_2$ and (c) $Cu_3(HITP)_2$.

4.2 SEM-EDX

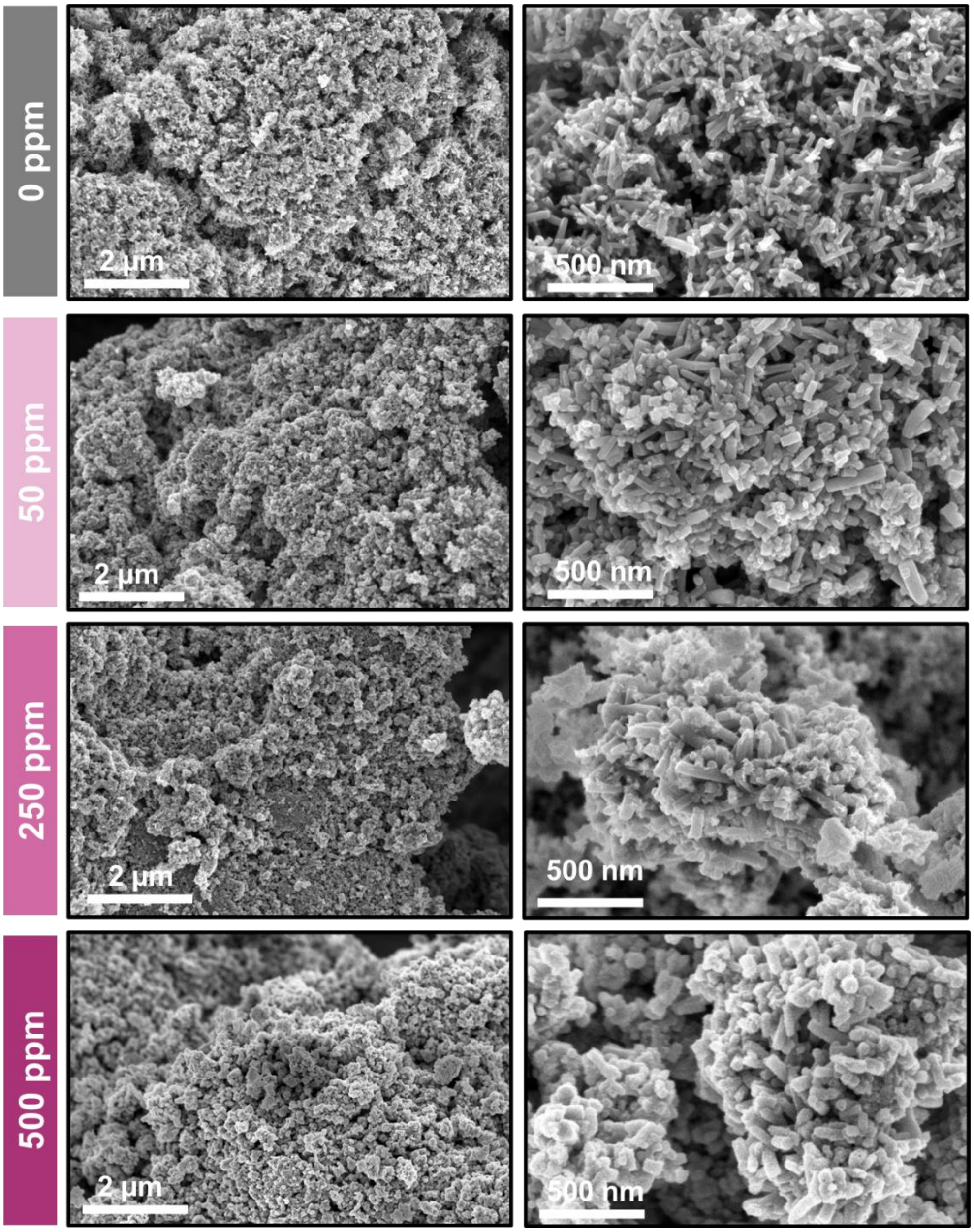

Figure S50. SEM micrographs of $Ni_3(HITP)_2$ crystals before after adsorption of 50, 250, and 500 ppm of MnO_4^- for 24 hours.

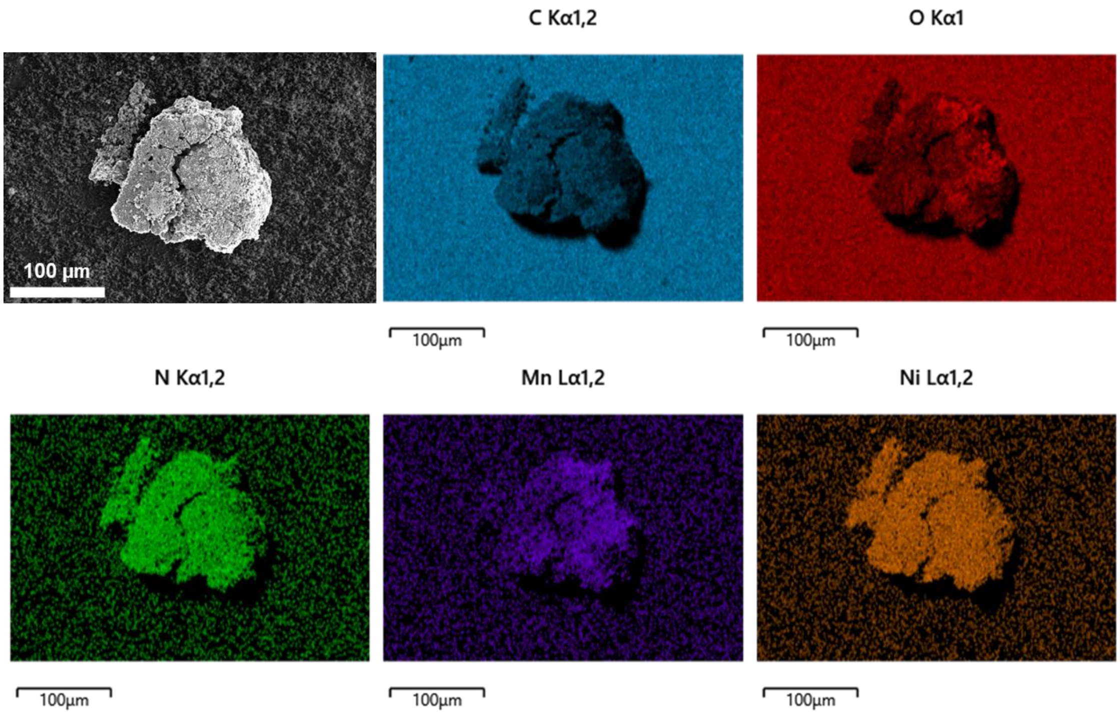

Figure S51. EDX elemental mapping images for $Ni_3(HITP)_2$ crystals following exposure to 50 ppm of MnO_4^- in DI water for 24 hours.

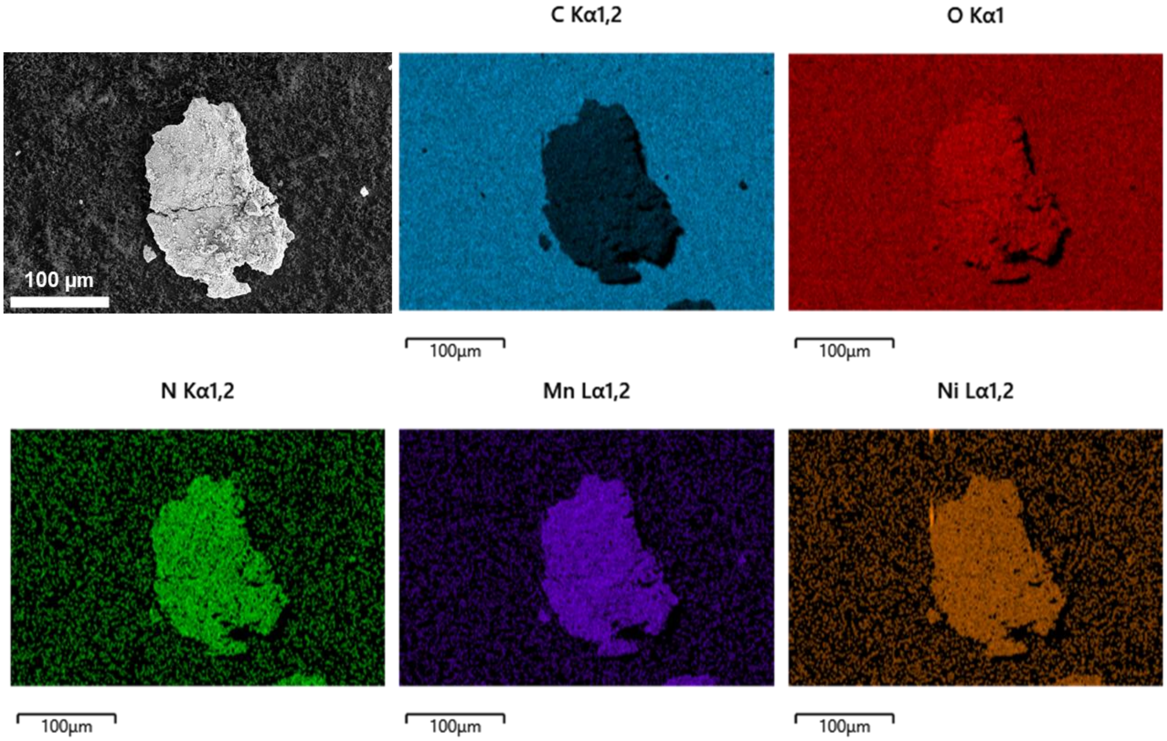

Figure S52. EDX elemental mapping images for $Ni_3(HITP)_2$ crystals following exposure to 250 ppm of MnO_4^- in DI water for 24 hours.

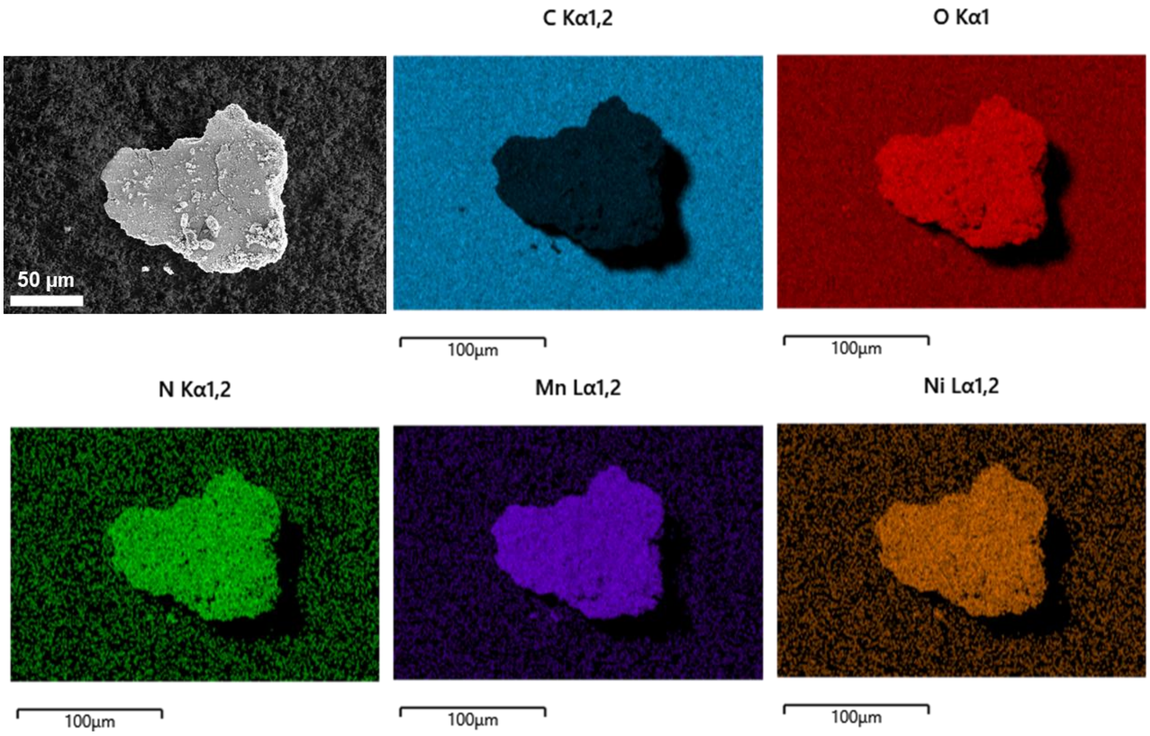

Figure S53. EDX elemental mapping images for $Ni_3(HITP)_2$ crystals following exposure to 500 ppm of MnO_4^- in DI water for 24 hours.

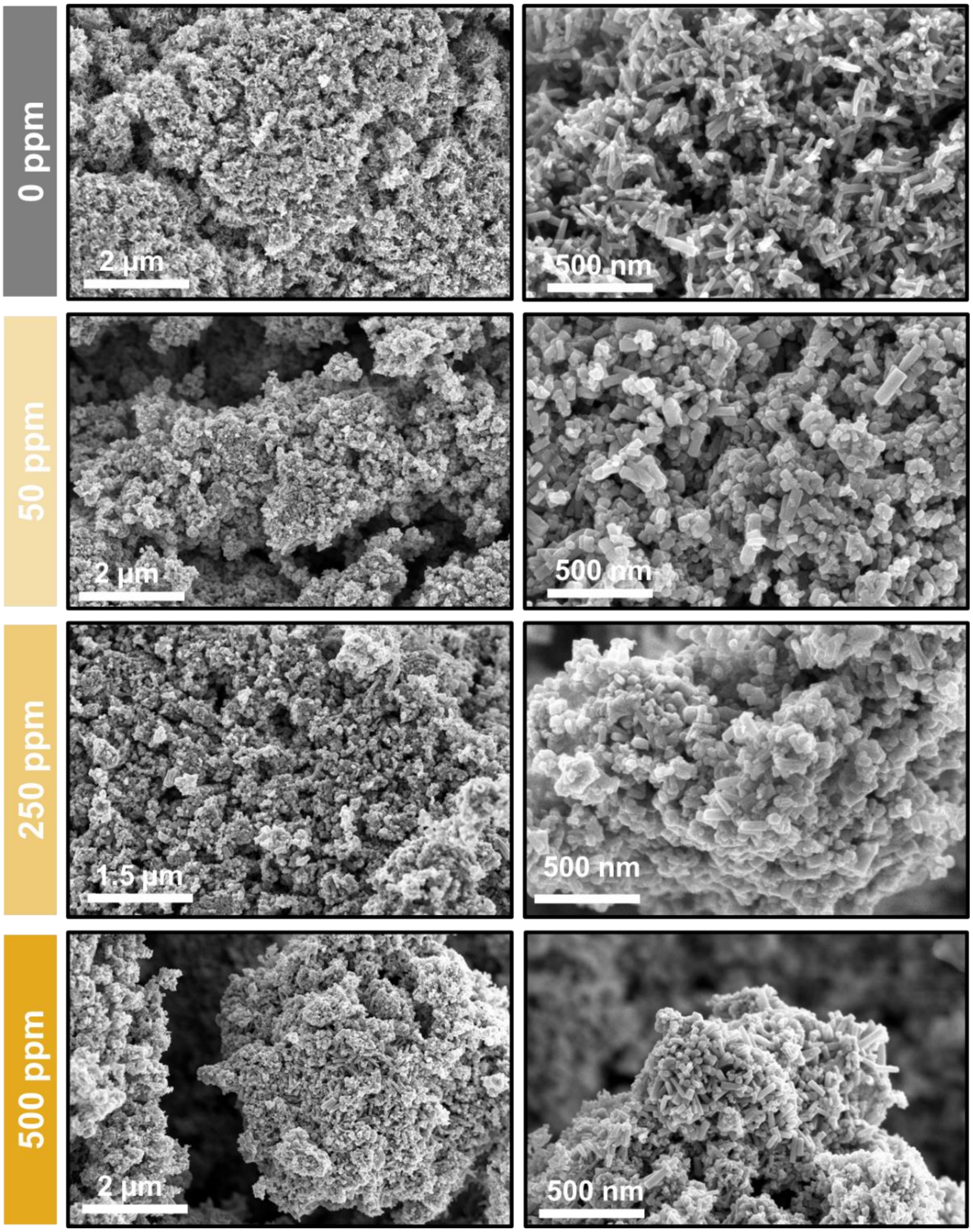

Figure S54. SEM micrographs of $Ni_3(HITP)_2$ crystals before and after adsorption of 50, 250, and 500 ppm of $Cr_2O_7^{2-}$ for 24 hours.

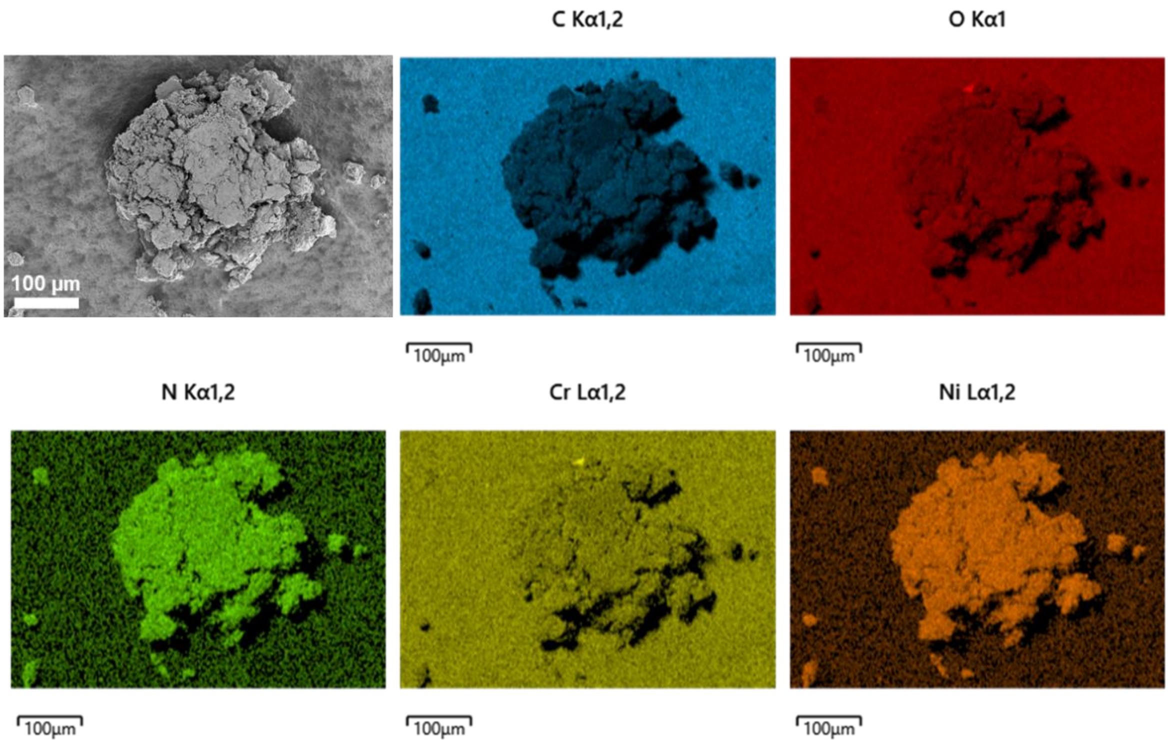

Figure S55. EDX Elemental mapping images of $Ni_3(HITP)_2$ crystals following exposure to 50 ppm of $Cr_2O_7^{2-}$ for 24 hours.

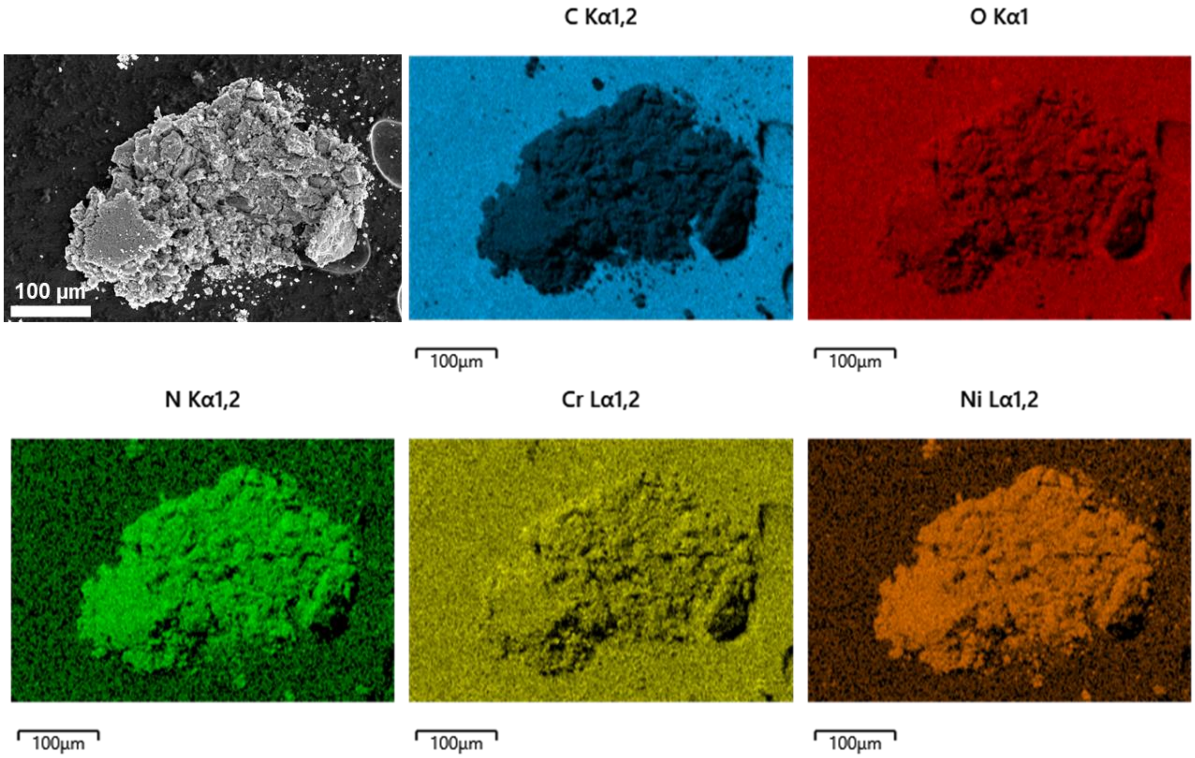

Figure S56. EDX Elemental mapping images of $Ni_3(HITP)_2$ crystals following exposure to 250 ppm of $Cr_2O_7^{2-}$ for 24 hours.

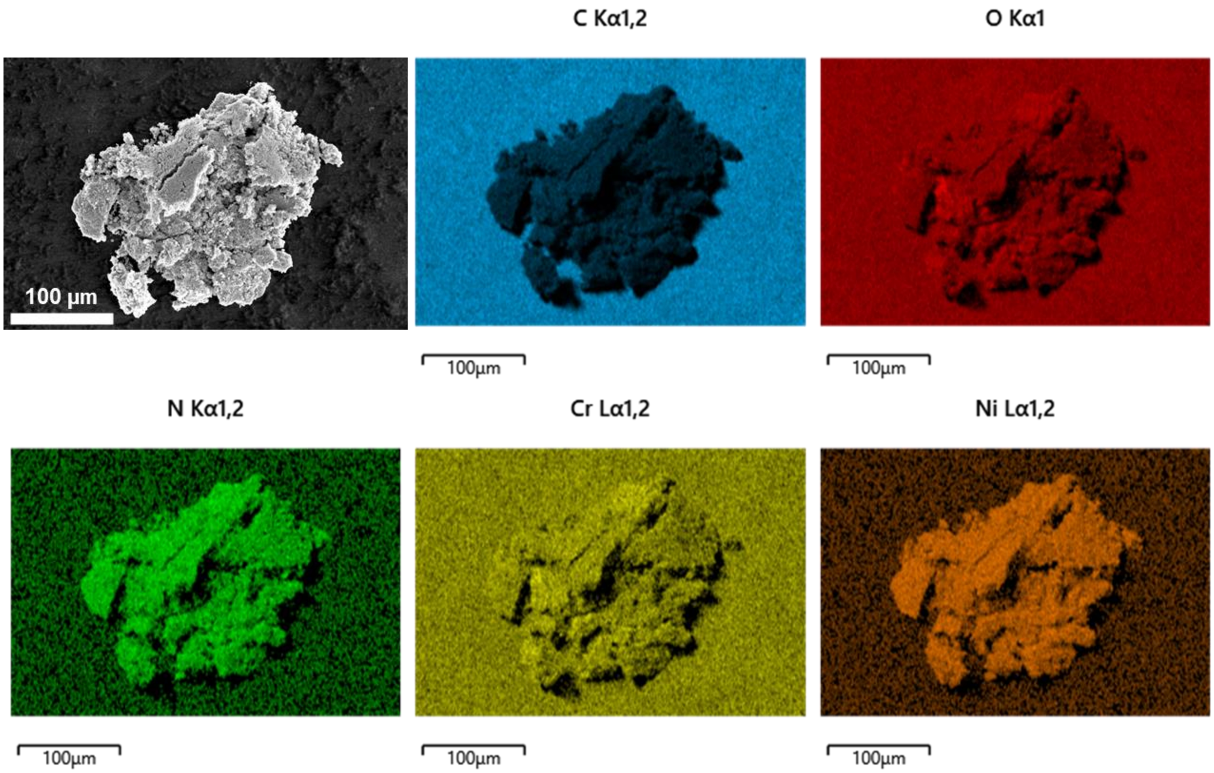

Figure S57. EDX Elemental mapping images of $Ni_3(HITP)_2$ crystals following exposure to 500 ppm of $Cr_2O_7^{2-}$ for 24 hours.

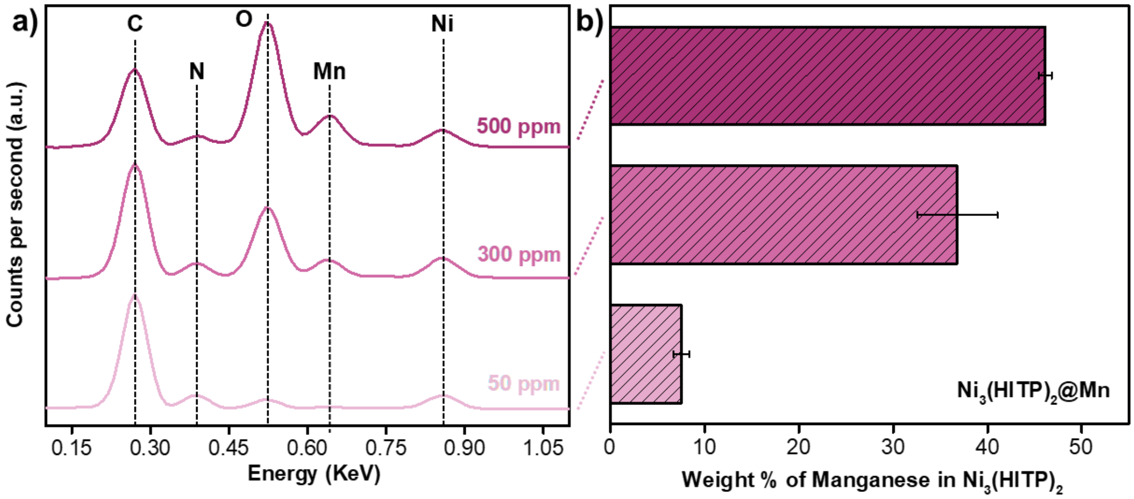

Figure S58. a) EDX spectra and b) weight % plots of $Ni_3(HITP)_2$ following adsorption of MnO_4^- at three different concentrations for 24 hours.

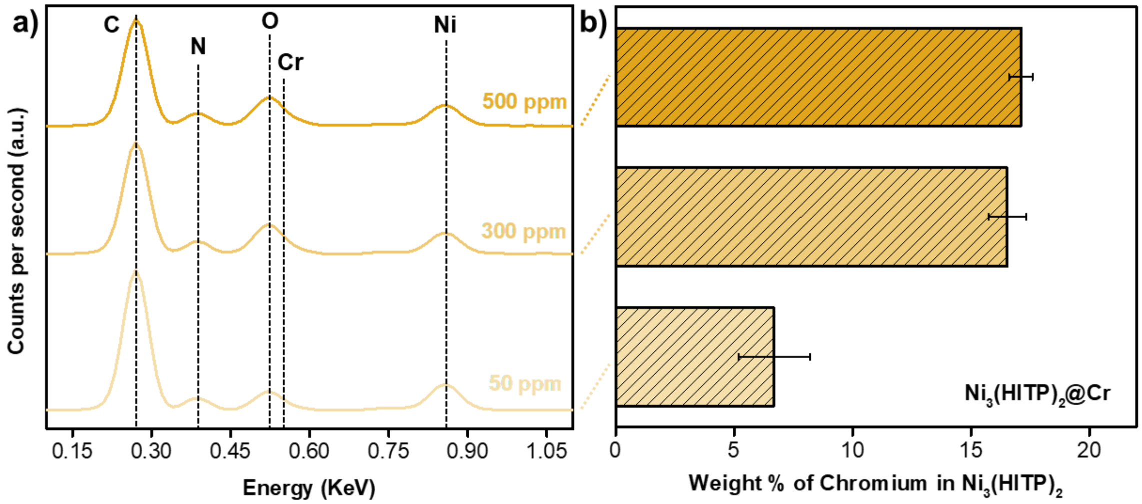

Figure S59. a) EDX spectra and b) weight % plots of $Ni_3(HITP)_2$ following adsorption of $Cr_2O_7^{2-}$ at three different concentrations for 24 hours.

4.3 XPS

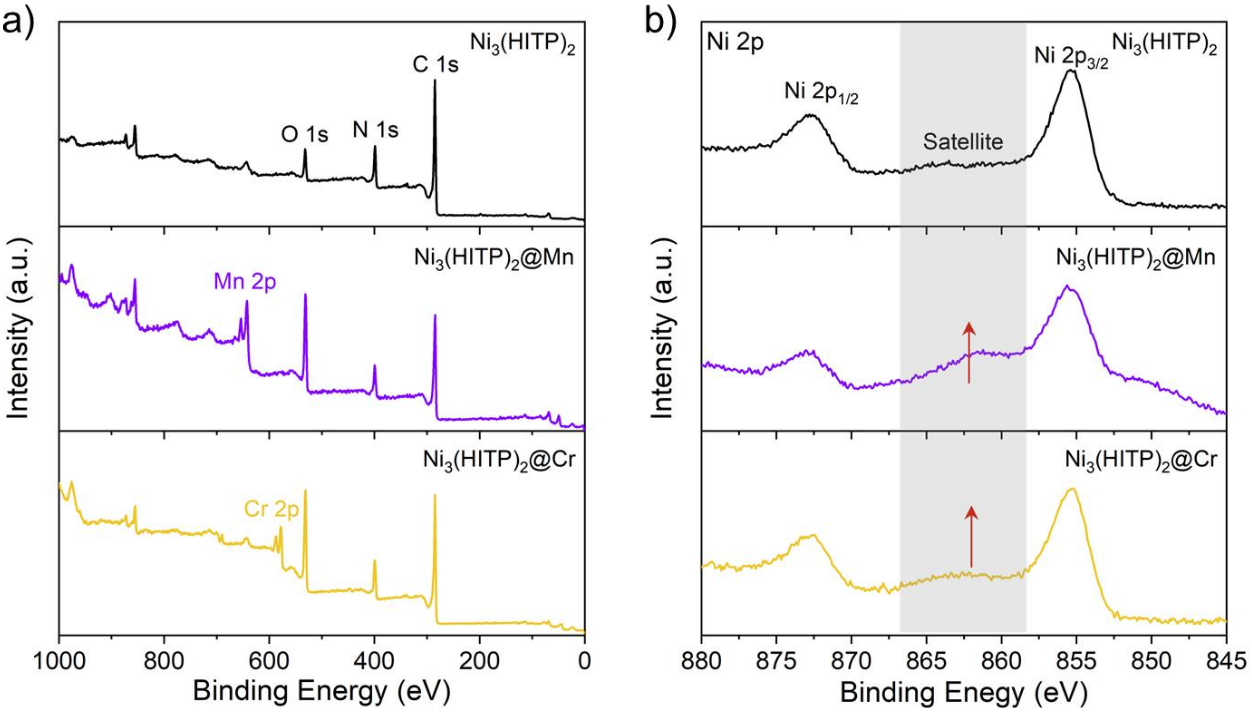

Figure S60. a) XPS survey spectra and b) high-resolution Ni 2p spectra of $Ni_3(HITP)_2$ MOF before and after adsorption of oxyanions.

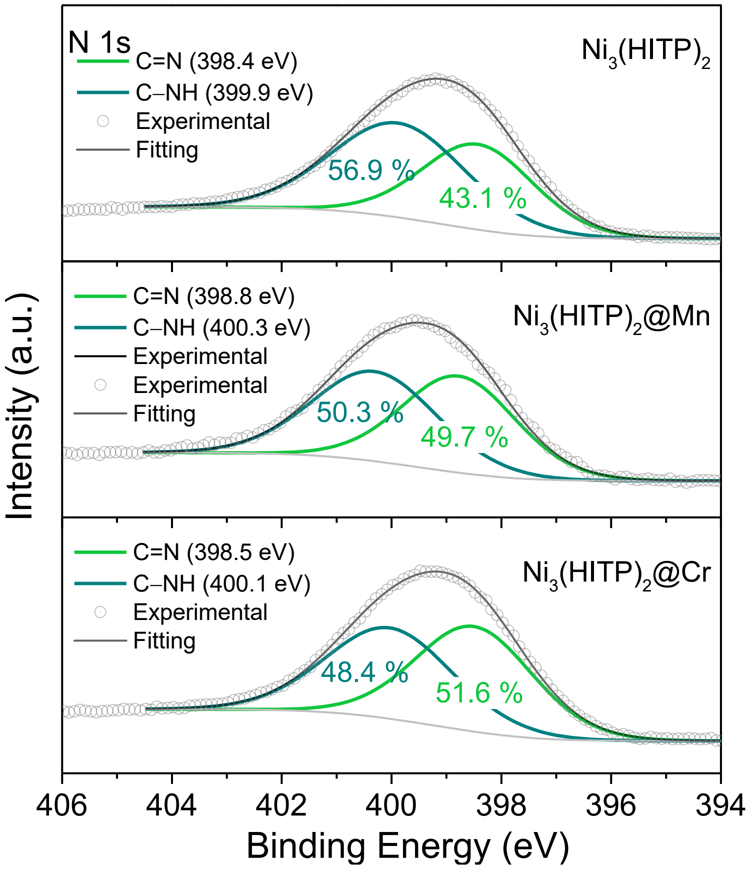

Figure S61. High-resolution N 2s XPS spectra of $Ni_3(HITP)_2$, before and after adsorption of MnO_4^- and $Cr_2O_7^{2-}$.

4.4 Additional Characterization

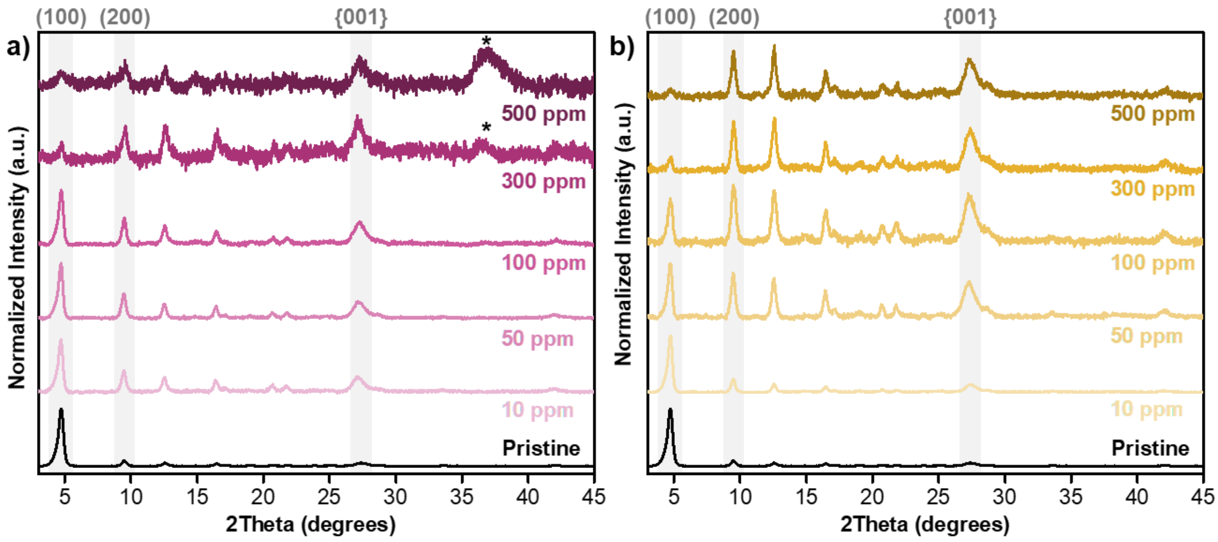

Figure S62. PXRD patterns of $Ni_3(HITP)_2$ after exposure to different concentrations of a) MnO_4^- and b) $Cr_2O_7^{2-}$ oxyanions for 24 hours. The asterisk "*" in part *a* indicates the formation of amorphous MnO_2 nanoparticles.

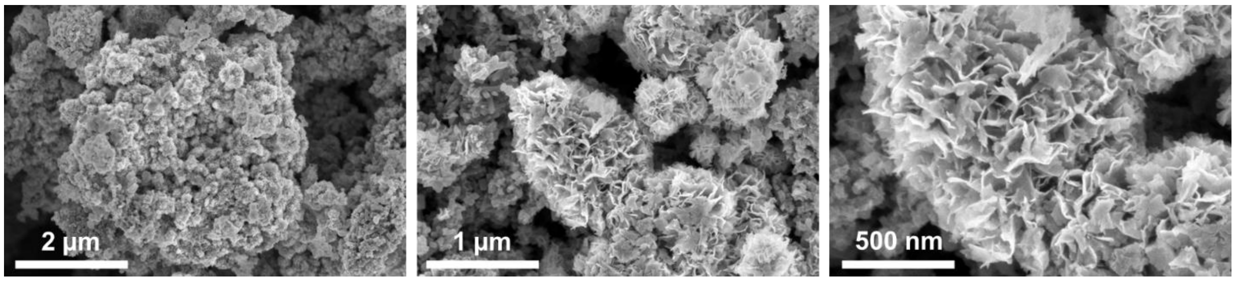

Figure S63. Representative SEM micrographs of $Ni_3(HITP)_2$ after adsorption of 1000 ppm of MnO_4^-, suggesting the formation of sheet-like structures of MnO_2.

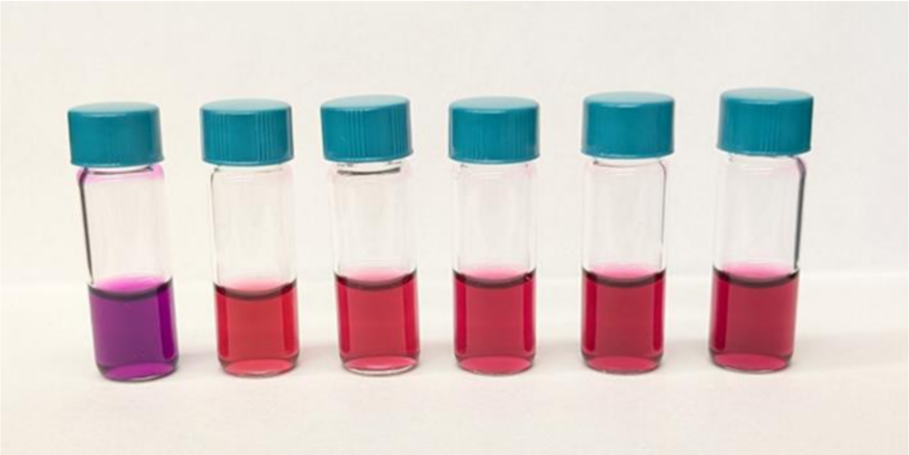

Figure S64. Optical photographs of 200 ppm MnO_4^- solutions before and after adsorption saturation experiments with $Ni_3(HITP)_2$. From left to right: stock solution before adsorption, and after adsorption for 32 hours, 24 hours, 9 hours, 6 hours, and 3 hours.

5. Simultaneous adsorption

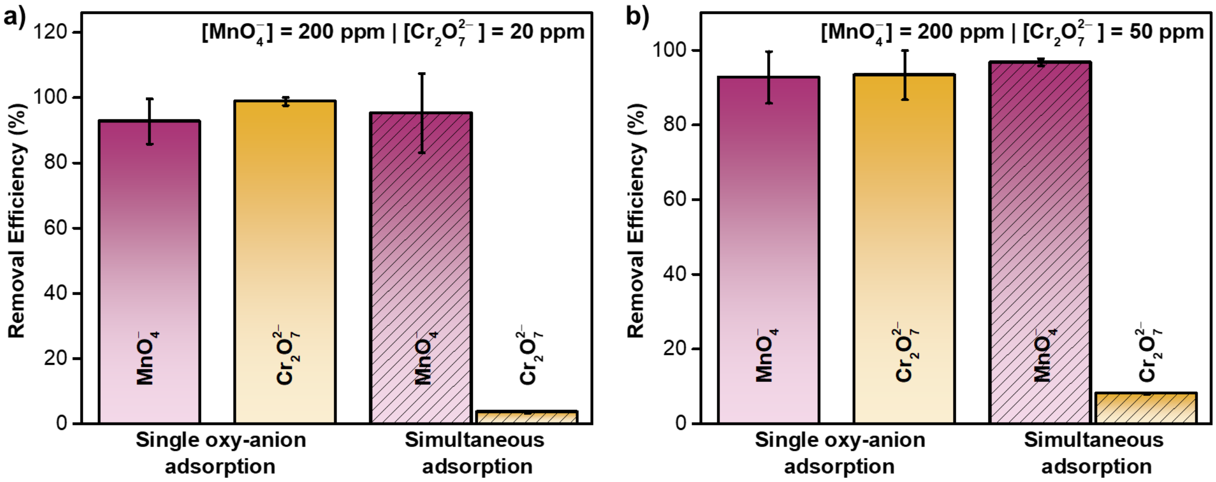

Figure S65. Comparison of the removal efficiency of $Ni_3(HITP)_2$ towards Mn(VII) and Cr(VI) in separate and mixed solutions. The starting concentration of Mn(VII) exceeds that of Cr(VI) in both plots.

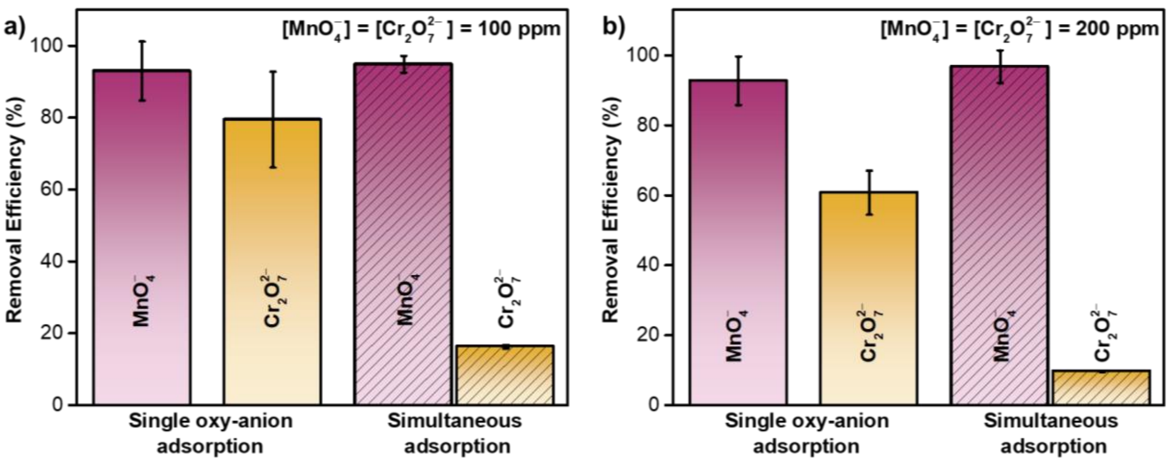

Figure S66. Comparison of the removal efficiency of $Ni_3(HITP)_2$ towards equimolar amounts of Mn(VII) and Cr(VI) in separate and mixed solutions.

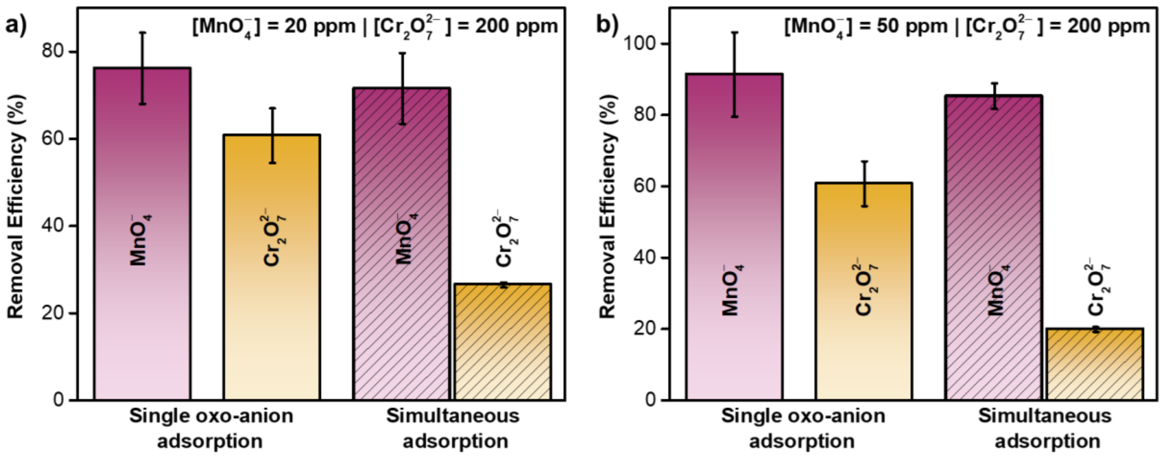

Figure S67. Comparison of the removal efficiency of $Ni_3(HITP)_2$ towards Mn(VII) and Cr(VI) in separate and mixed solutions. The starting concentration of Cr(VI) exceeds that of Mn(VII) in both plots.

6. Multi-scale modeling

A starting slipped-parallel arrangement of the $Ni_3(HITP)_2$ MOF was obtained from a CIF file built from PXRD measurements. Both geometry and unit cell parameters were optimized at the density functional theory level (DFT) level. The converged configuration was then used to estimate the interaction with adsorbate species such as $HMnO_4$ and $H_2Cr_2O_7$. In the case of the $H_2Cr_2O_7$, given its elongated structure, we had to double the unit cell in the z-direction. Besides considering the

slipped-parallel arrangement, we also examined ABC-type stacking sequences. All the simulations were carried out through the Quantum Espresso package[37] using the generalized gradient approximation (GGA) with the Perdew–Burke–Ernzerhof (PBE) functional.[38] Dispersion corrections were managed with the Grimme-D3 parametrization.[39] Wave function and charge density cutoffs for the plane-wave basis sets of 60 and 600 Ry were employed, respectively. Single-particle wave functions were calculated spin-unrestricted by applying smearing of the one-particle levels of 0.002 Ry. Periodic boundary conditions were applied in all directions. In the case of bilayers, the reciprocal space was sampled by a (1x1x3) k-point grid after verifying that a doubling of the k-point grid (2x2x6) did not introduce any quantitative difference. In the case of 4L-(1x1) cells, the reciprocal space was sampled at the gamma point only.

Non-periodic DFT calculations were carried out via the Gaussian16 software. considering finite-size models systems comprising 2 partial layers of the $Ni_3(HITP)_2$ material in a stacking configuration mimicking slipped-parallel arrangement. 6-31G(d,p), and def2-TZVPP for Ni and Mn basis sets were used, in conjunction with the hybrid B3PW91 xc-functional for the description of exchange and correlation effects.

The structural optimization of the 2L-(1x1) $Ni_3(HITP)_2$ unit cell produced the structure shown in the left panel of **Figure S68(a)**: optimized values of the unit cell parameters resulted in excellent agreement with the experimental data: lattice vectors were 21.88, 21.88, and 6.63 Å (to be compared with 21.85, 21.74 and 6.55 Å from the PXRD data), whereas optimized angles were 90.0, 90.0, and 59.9 degrees (to be compared with 90.0, 90.0 and 60.2 degrees from the PXRD data). These data agree with the previous literature.[40] The lowest-energy electronic potential energy surface (PES) was spin-unpolarized with a finite DOS at the Fermi level, which was in agreement with the metallic character of the MOF. Near the Fermi level, see left panel of **Figure S68(c)**, the DOS is dominated by linker states, whereas the contribution from the metal centers

is mainly located at lower energies. In **Figure S68(b)**, the 4L-(1x1) ABCB unit cell with individual layers organized in stacking sequences analogous to those of fcc crystals (ABC) is also shown. The adsorbate/MOF interaction is estimated by reporting adhesion energy: this quantity is calculated as the difference between the total energy of the complex and the energies of the two separate fragments (adsorbate and MOF). Negative values indicate favorable adsorption.

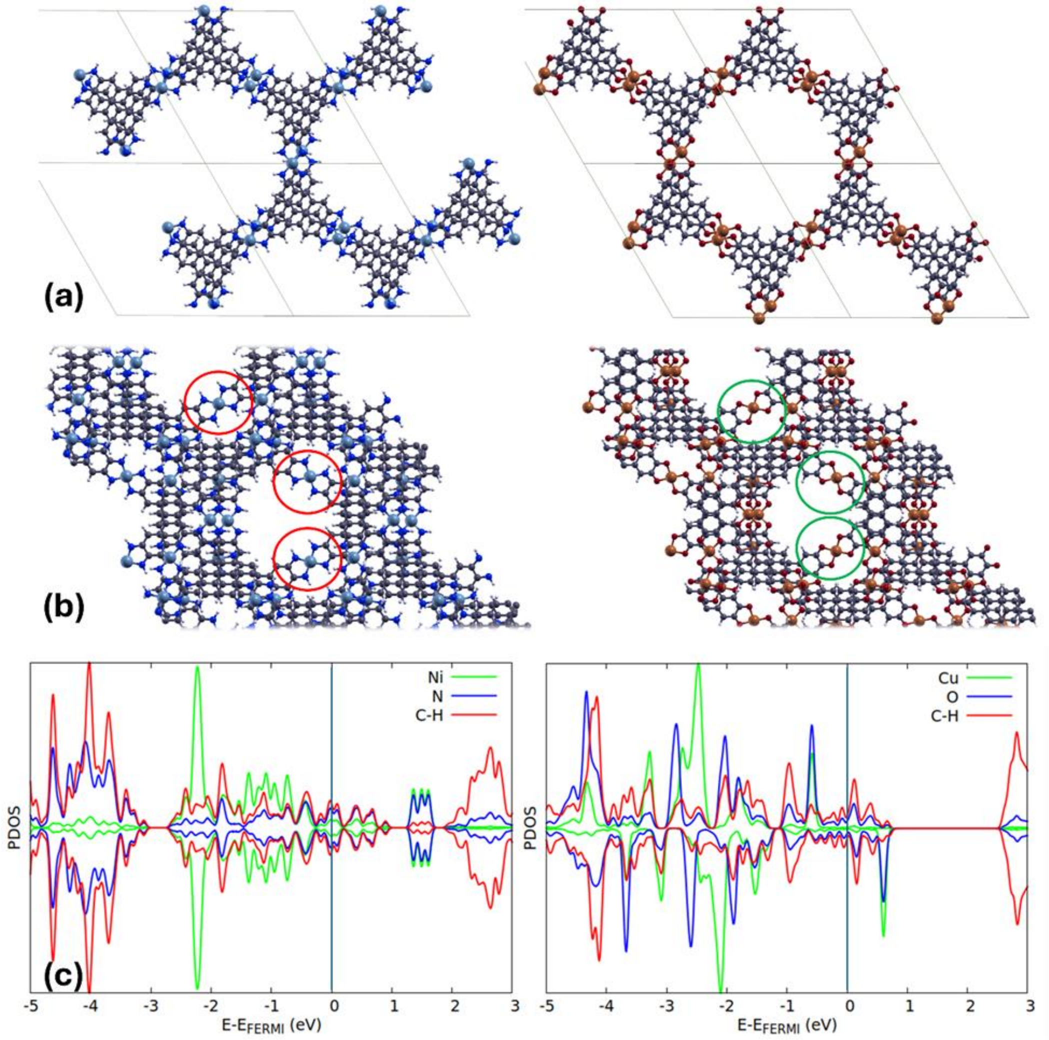

Figure S68. structure of the unit cells, replicated twice in both *x* and *y* directions, of the $Ni_3(HITP)_2$ (left) and $Cu_3(HHTP)_2$ (right) systems adopting a slipped-parallel arrangement (a) and ABC mode (b). Red and green circles highlight the presence of OMS (open-metal sites); (c) PDOS of the structures in (a), where the main atomic contributions are plotted. Color coding: Ni light blue, Cu orange, N blue, O red, C gray, and H white.

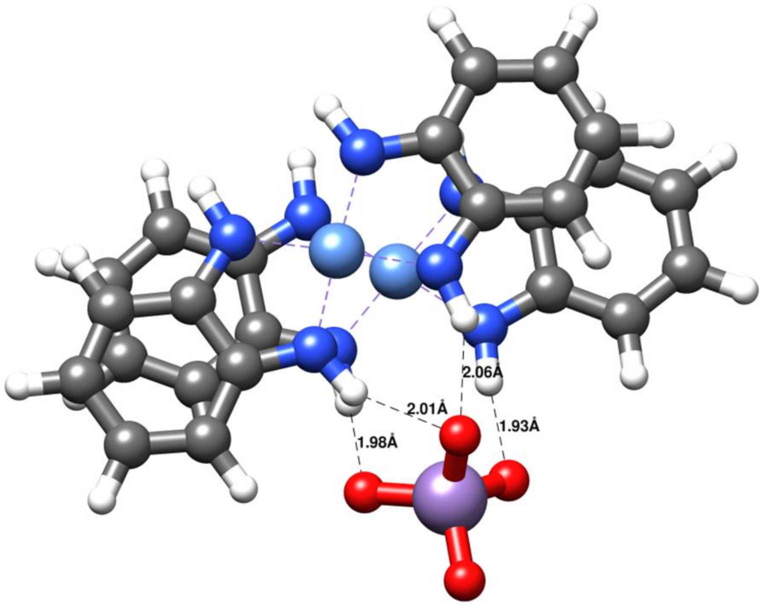

Figure S69. Finite models of two partial layers of the $Ni_3(HITP)_2$ material interacting with MnO_4^- in vacuo. Most characteristic distances (in Angstrom) between the adsorbate ions and the MOF are reported. Color coding: Ni light blue, Mn purple, N blue, O red, C gray, and H white.

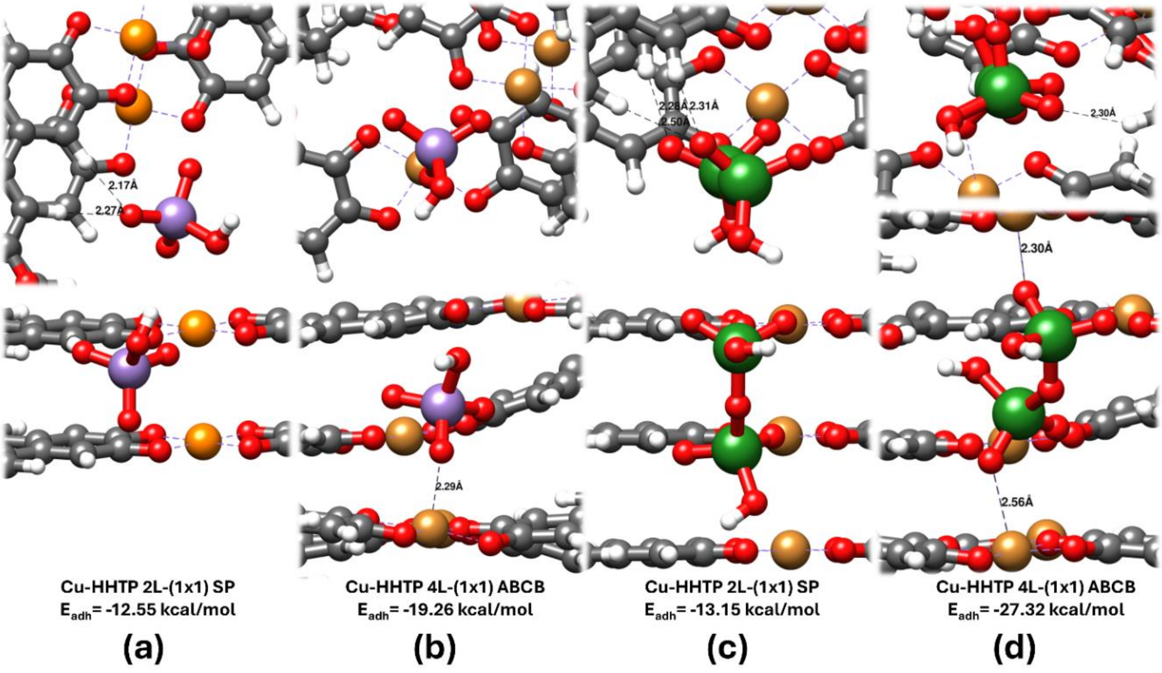

Figure S70. optimized configurations and correspondent adhesion energies (in kcal/mol) of the $HMnO_4$ and $H_2Cr_2O_7$ adsorbates interacting with the $Cu_3(HHTP)_2$ system, in both SP arrangement (a-b) and ABCB reconstructions (c-d). Color coding: O red, C gray, H white, Mn purple, and Cr green.

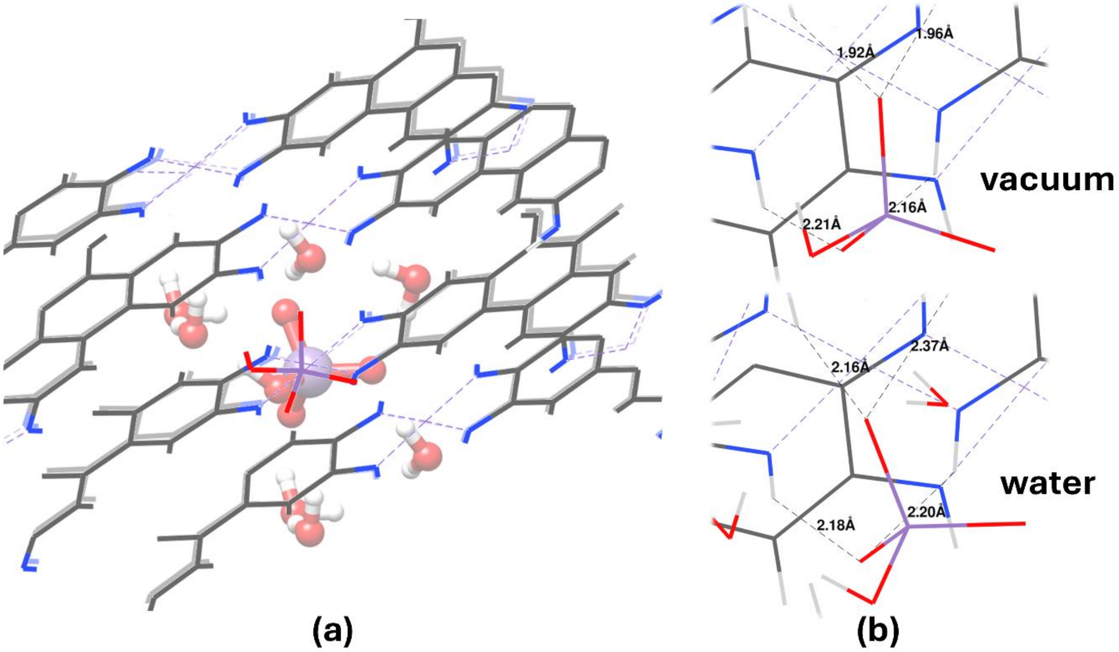

Figure S71. (a) superimposed optimized configurations of the $HMnO_4$ adsorbate on the MOF (in its slipped-parallel arrangement) in vacuo (wire representation) and when surrounded by four water molecules (ball and stick representation). (b) characteristic hydrogen bond distances (in Angstrom) in vacuum and in water. Color coding: Ni light blue, Cu orange, N blue, O red, C gray, and H white, Mn purple.

7. Electronic textiles based on $Ni_3(HITP)_2$

7.1 Fabrication method

$Ni_3(HITP)_2$ was deposited onto cotton fabrics using a layer-by-layer (LbL) approach. Plasma-cleaned 2 x 5 cm^2 cotton textile swatches were first immersed in 50 mL deionized (DI) water, ethanol, and acetone consecutively, undergoing ultrasonic cleaning for 10 minutes in each solvent before getting air-dried overnight. For the deposition process, the fabric swatches were first immersed in 10 mL of a 50 mM $Ni(OAc)_2$ aqueous solution for a few seconds, followed by drying in an oven set at 50 °C for 20 minutes. The swatches were then rinsed by immersion in 20 mL of DI water for 1 minute, dried again at 50 °C for 20 minutes, and subsequently immersed in 10 mL of a 1:1 DI water:DMF solution containing 20 mM HATP·6HCl and 100 equivalents of NaOAc (relative to the ligand) for a few seconds. The swatches were then dried in a vacuum oven at 50 °C for 60 minutes, followed by washing with DI water and ethanol, and drying again for 30 minutes.

This deposition cycle was repeated 10 times to achieve uniform MOF growth. The resulting textile swatches were then activated by soaking in DI water for 2 days, followed by solvent exchange with acetone for an additional 2 days. Finally, the swatches were dried in a vacuum oven set at 72 °C for 1 day before characterization and testing.

To determine the mass loading of $Ni_3(HITP)_2$ on the textile, we measured the mass of cotton fabric swatches (2 x 5 cm^2) before and after ten cycles of MOF deposition. The difference in mass was used to calculate the amount of MOF loaded per one cm^2. Based on four independent experiments, the average mass loading was calculated to be 8.5 ± 1.5 mg per cm^2. The individual results are summarized in **Table S1**.

Sample	Mass of 2 x 5 cm^2 bare swatch (mg)	Mass of swatch after MOF deposition (mg)	$Ni_3(HITP)_2$ loading (in mg per cm^2)
**Swatch 1**	58.8	162.4	10.3
**Swatch 2**	56.2	125.3	6.9
**Swatch 3**	55.4	133.6	7.8
**Swatch 4**	60.8	149.9	8.9

Table S1. Mass loading of $Ni_3(HITP)_2$ on cotton textile (in mg per cm^2) after 10 deposition cycles.

Cotton fabrics are rich in free, terminal hydroxyl groups, which serve as active sites for interactions with MOF precursors through both, intermolecular forces, and potential covalent coordination.[41, 42] We hypothesize that these surface hydroxyls facilitate adsorption of Ni^{2+} ions and promote coordination interactions, while also enabling hydrogen bonding and electrostatic interactions with the deprotonated HATP·6HCl ligand.[43-45] This interface likely contributes to the robust integration and growth of $Ni_3(HITP)_2$ on the cotton substrate. Additionally, we note that our LbL approach enabled the formation of $Ni_3(HITP)_2$ coatings on cotton without the need for chemical pretreatments such as carboxymethylation,[46, 47] silanization,[48] and mercerization,[49] which are commonly required in other systems involving different substrates, deposition methods, and MOF

materials. Likewise, the resulting MOF@textile composite exhibited outstanding structural stability (**Figure S81**), which we attribute to a combination of coordination bonding, hydrogen bonding, electrostatic interactions, and mechanical interlocking between the MOF layer and the fibrous cotton substrate.

Although the initial soaking step is performed at room temperature, the swatches are immediately transferred to an oven maintained at 50 °C, where the processes of nucleation and crystallization of $Ni_3(HITP)_2$ occur. This relatively mild temperature is sufficient to drive MOF formation, as prior reports in the literature have shown successful synthesis of bulk $Ni_3(HITP)_2$ at or near 65 °C. Moreover, several studies have demonstrated that $Ni_3(HITP)_2$ and related triphenylene-based MOFs can be synthesized under ambient conditions (~25 °C).[4, 50, 51] These findings support the feasibility of our low-temperature method and help explain the formation and stability of the MOF layer on the textile substrate.

The cyclic adsorption-desorption tests shown in **Figure 7d-e** were conducted using 1 cm x 1 cm swatches of $Ni_3(HITP)_2$@textile, each containing a MOF loading of ≈ 8.5 ± 1.5 mg. The swatches were placed in 20 mL vials containing 13 mL of 25 ppm oxyanion solutions (MnO_4^- or $Cr_2O_7^{2-}$), maintaining a solution volume-to-MOF mass ratio of approximately 1.5. The mixtures were stirred at room temperature for 24 hours. After adsorption, the solutions were filtered using PTFE syringe filters to remove any suspended MOF particles or textile fibers that may have frayed out during stirring. The filtrates were then analyzed by ICP-MS to quantify the residual concentration of the oxyanions. To regenerate the textile, each swatch was soaked in 0.1 M HCl for 4 hours on a shaker to desorb the adsorbed oxyanions. The textiles were then rinsed with deionized water followed by acetone, and dried in a vacuum oven at 70 °C for 12 hours. This adsorption-desorption cycle was repeated 32 times to evaluate the long-term recyclability of $Ni_3(HITP)_2$@textile towards both oxyanions.

7.2 Characterization

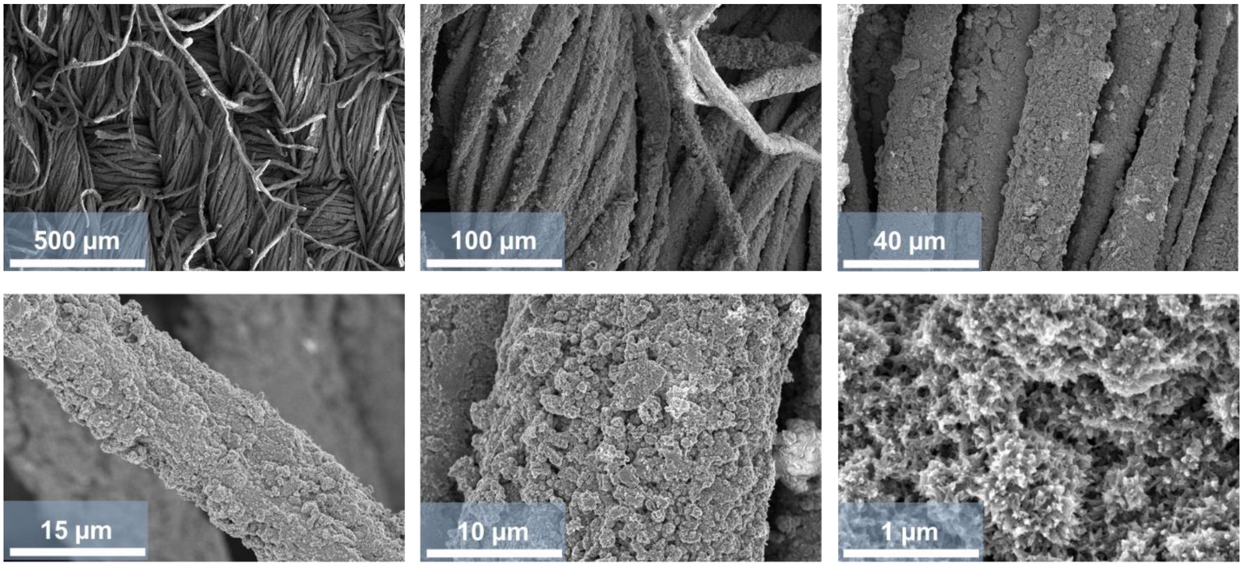

Figure S72. SEM micrographs of pristine $Ni_3(HITP)_2$@textile at different magnifications, indicating the formation of rod-like crystals uniformly coating the textile surface.

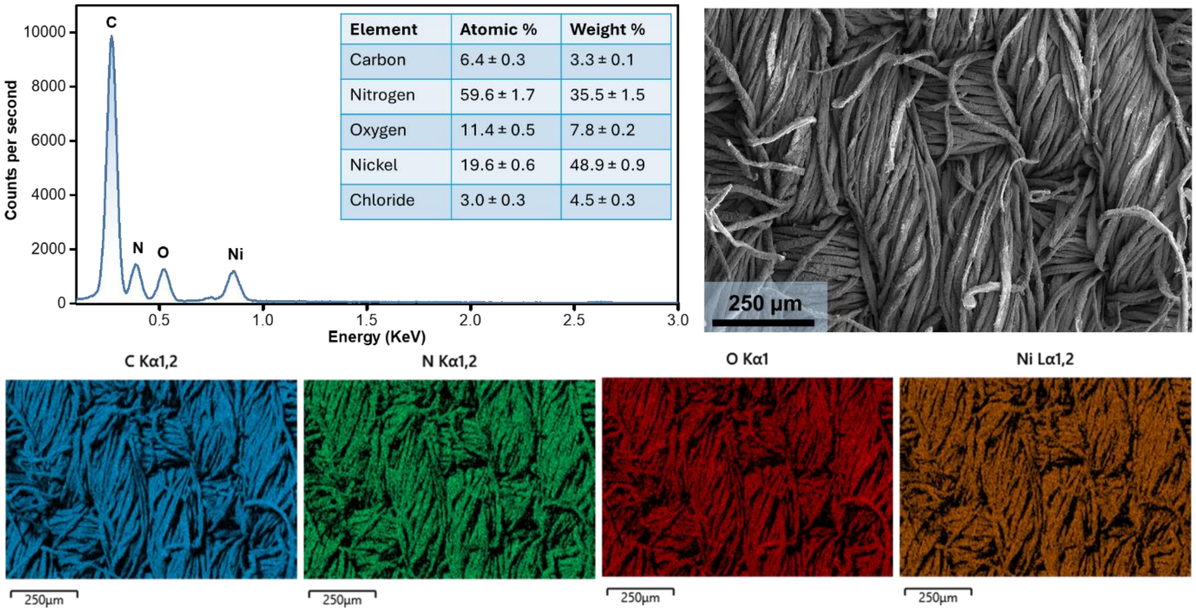

Element	Atomic %	Weight %
Carbon	6.4 ± 0.3	3.3 ± 0.1
Nitrogen	59.6 ± 1.7	35.5 ± 1.5
Oxygen	11.4 ± 0.5	7.8 ± 0.2
Nickel	19.6 ± 0.6	48.9 ± 0.9
Chloride	3.0 ± 0.3	4.5 ± 0.3

Figure S73. EDX spectrum and elemental mapping images of pristine $Ni_3(HITP)_2$@textile.

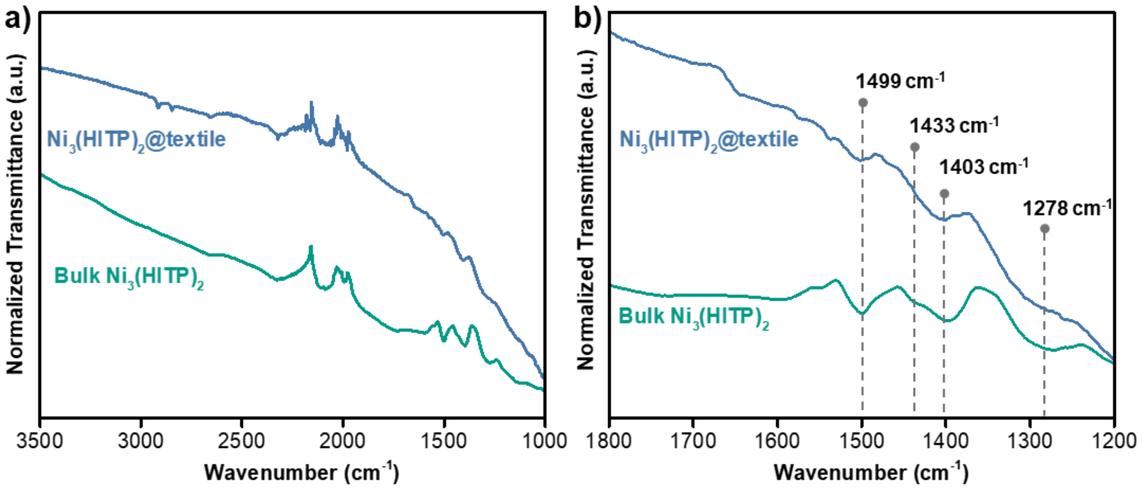

Figure S74. ATR-IR of bulk $Ni_3(HITP)_2$ and $Ni_3(HITP)_2$@textile.

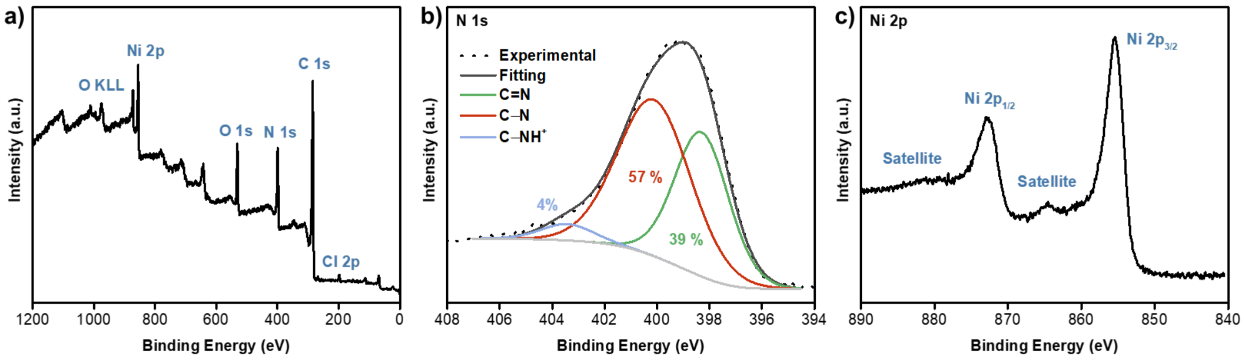

Figure S75. XPS measurements for $Ni_3(HITP)_2$@textile. (a) XPS survey spectrum, indicating the presence of C, N, O, Cl, and Ni. High-resolution spectra of b) N 1s and (c) Ni 2p elements.

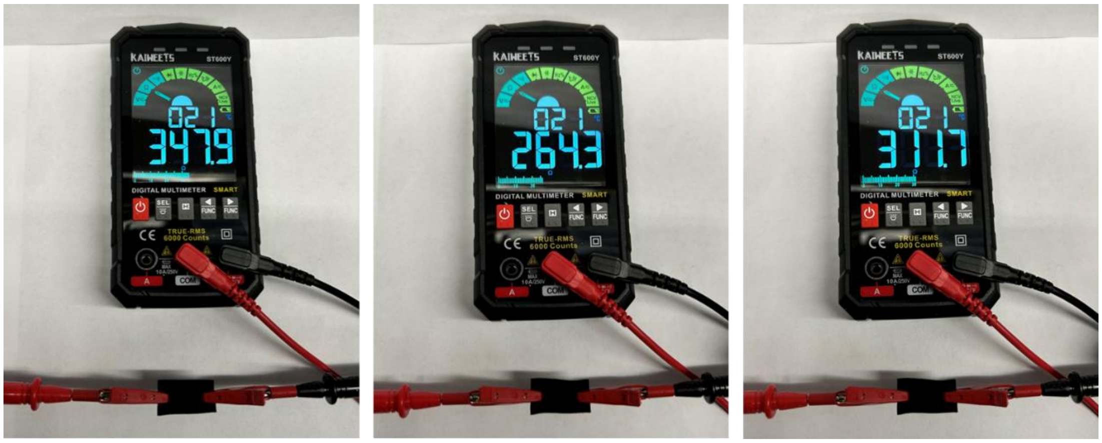

Figure S76. Sheet resistance values of flat swatches $Ni_3(HITP)_2$@textile (in Ω).

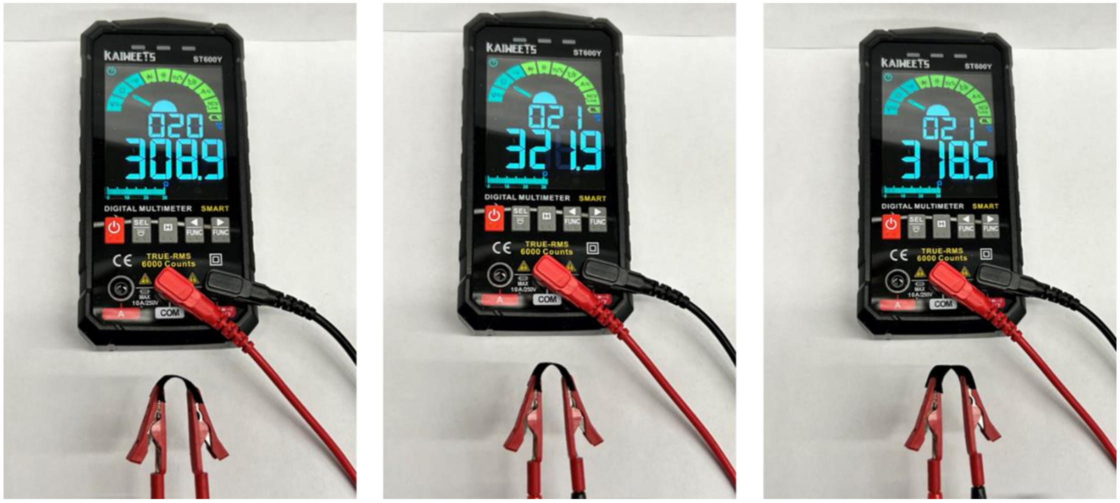

Figure S77. Sheet resistance values of $Ni_3(HITP)_2$@textile upon bending (in Ω).

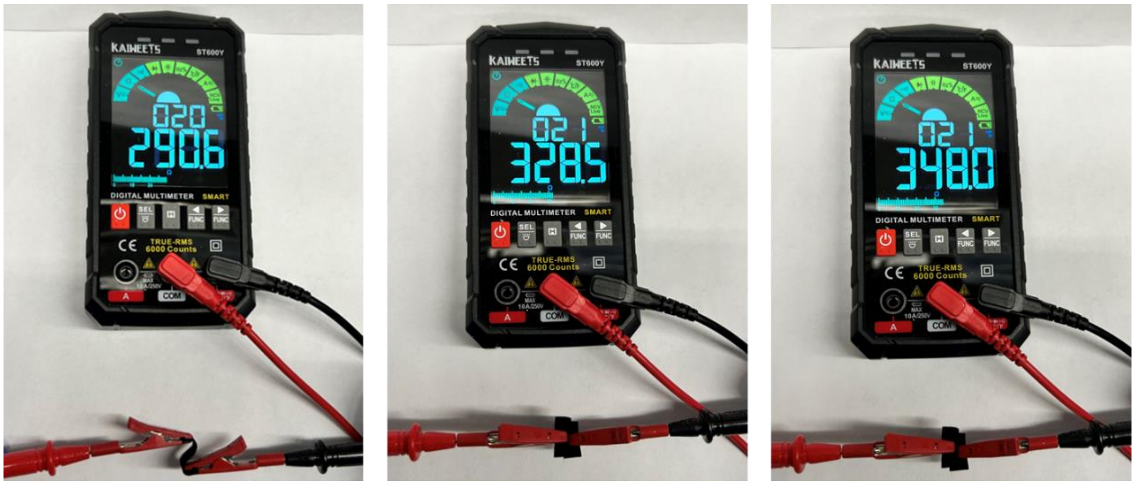

Figure S78. Sheet resistance values of $Ni_3(HITP)_2$@textile upon twisting (in Ω).

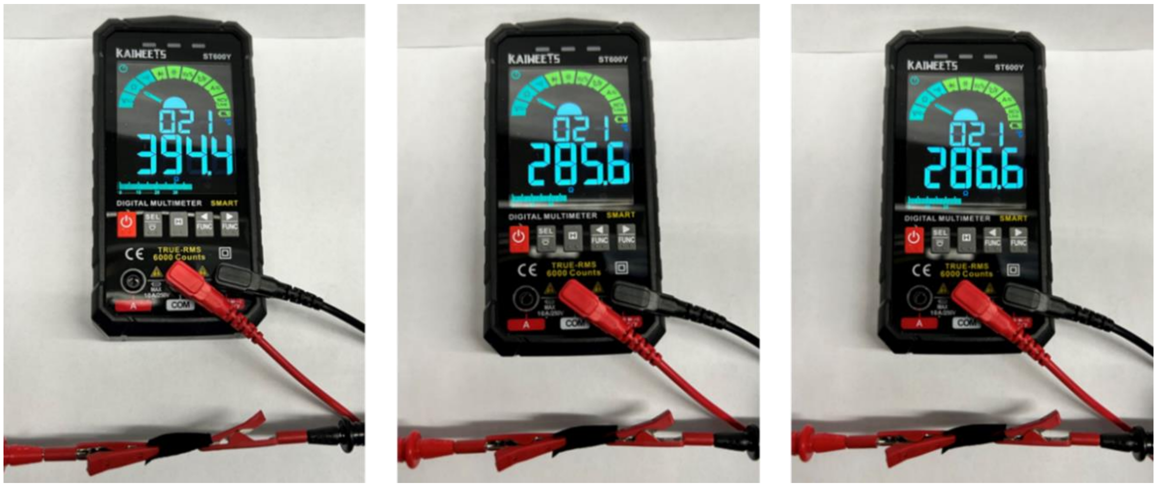

Figure S79. Sheet resistance values of curled swatches of $Ni_3(HITP)_2$@textile (in Ω).

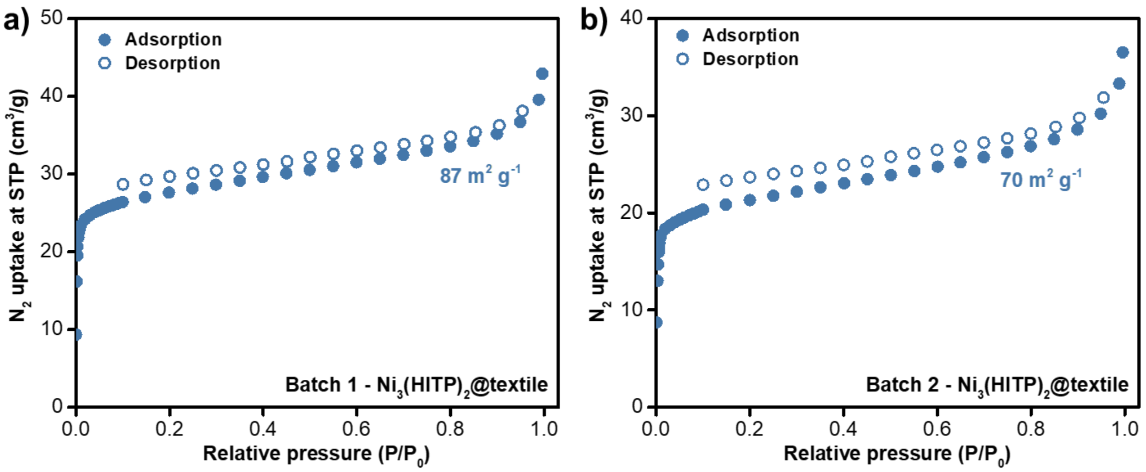

Figure S80. BET surface area measurements at 77K of two distinct batches of $Ni_3(HITP)_2$@textile.

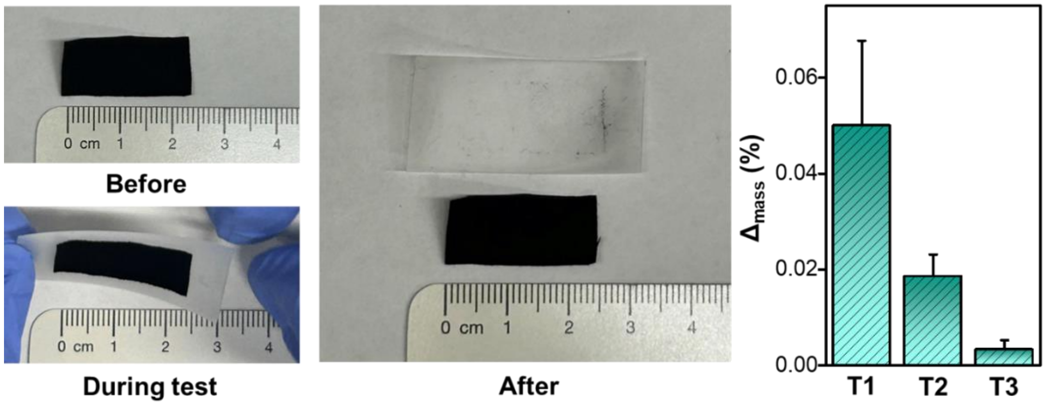

Figure S81. Photographs of $Ni_3(HITP)_2$@textile swatches during Scotch tape test (left) and change in mass after multiple tests on the same swatch (right).

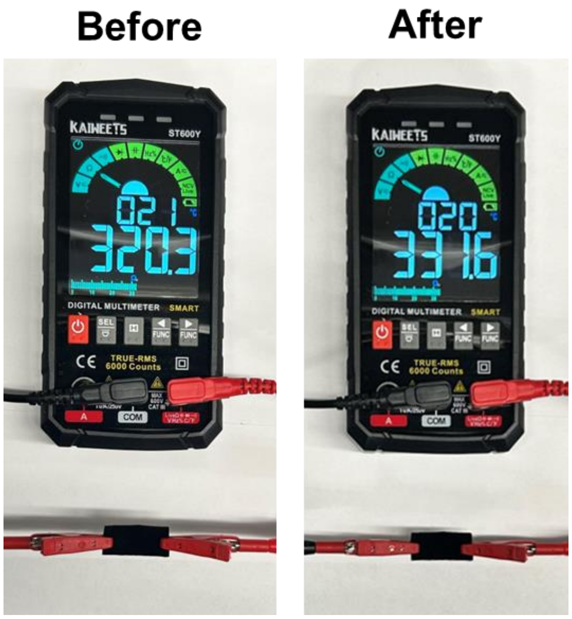

Figure S82. Sheet resistance values of a $Ni_3(HITP)_2$@textile swatch before (left) and after (right) the scotch tape test.

7.3 Filtration performance and characterization post-adsorption

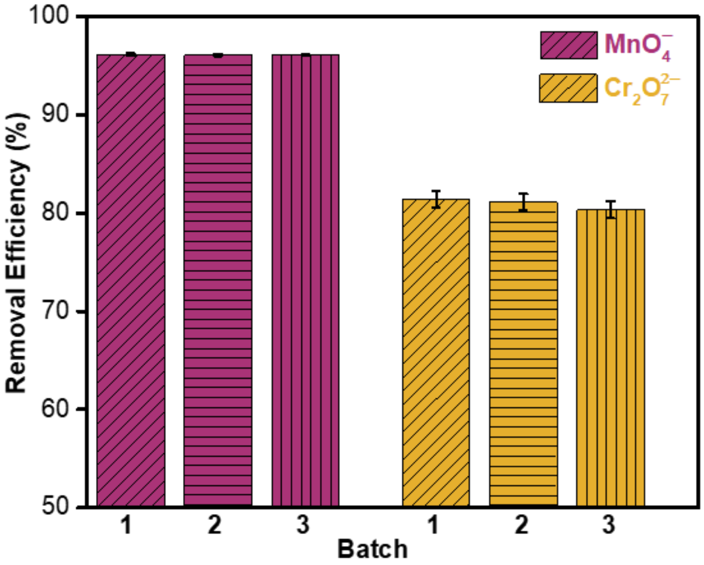

Figure S83. Removal efficiency of three distinct batches of $Ni_3(HITP)_2$@textile towards 10 ppm of MnO_4^- (violet) and 30 ppm of $Cr_2O_7^{2-}$ (yellow) using the filtration setup illustrated in **Figure 7a**.

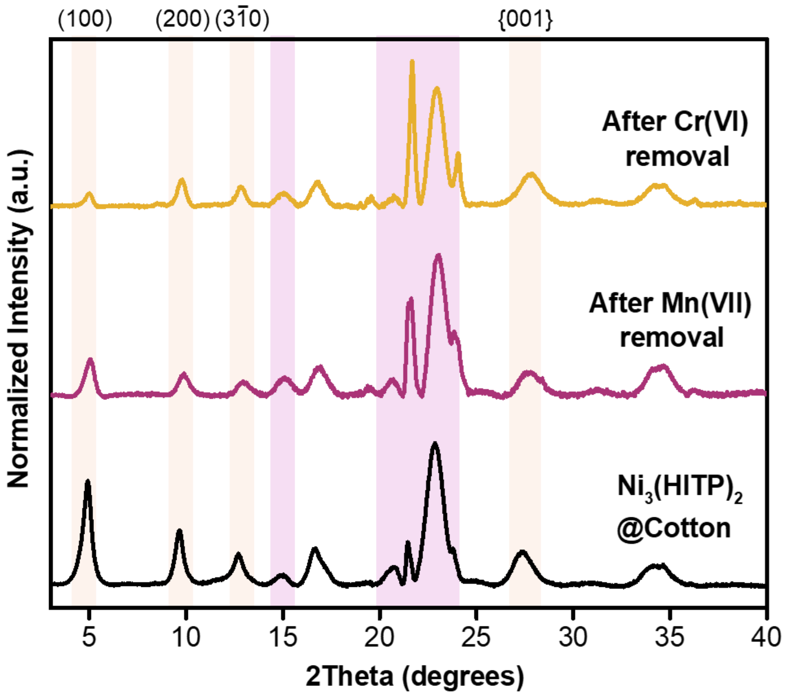

Figure S784. PXRD patterns of $Ni_3(HITP)_2$@textile before and after adsorption of 100 ppm of MnO_4^- and $Cr_2O_7^{2-}$.

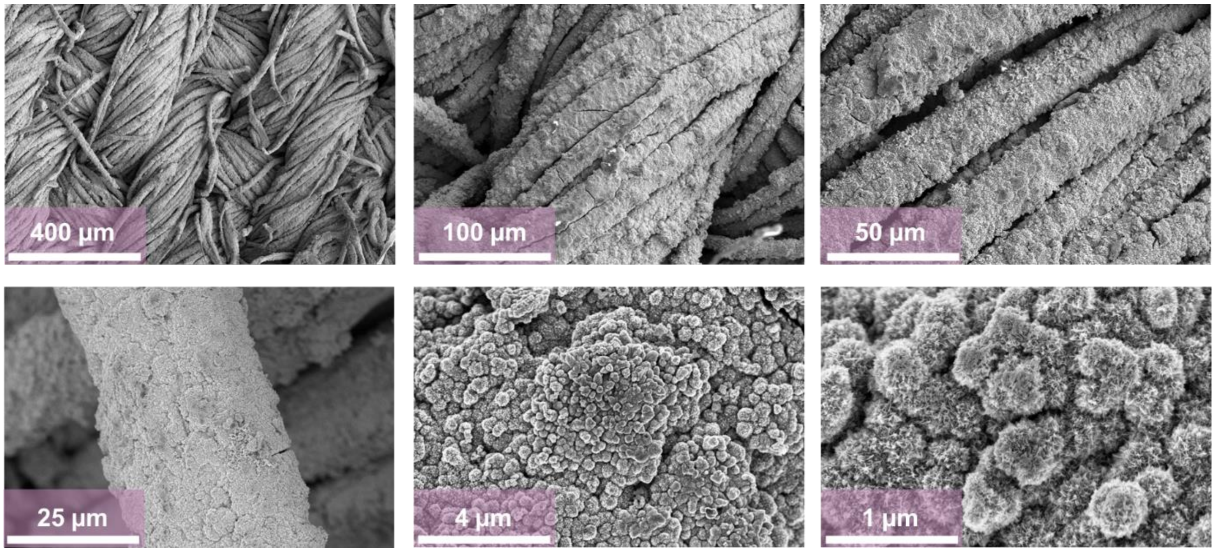

Figure S85. SEM micrographs of $Ni_3(HITP)_2$@textile at different magnifications after adsorption of 100 ppm of MnO_4^-.

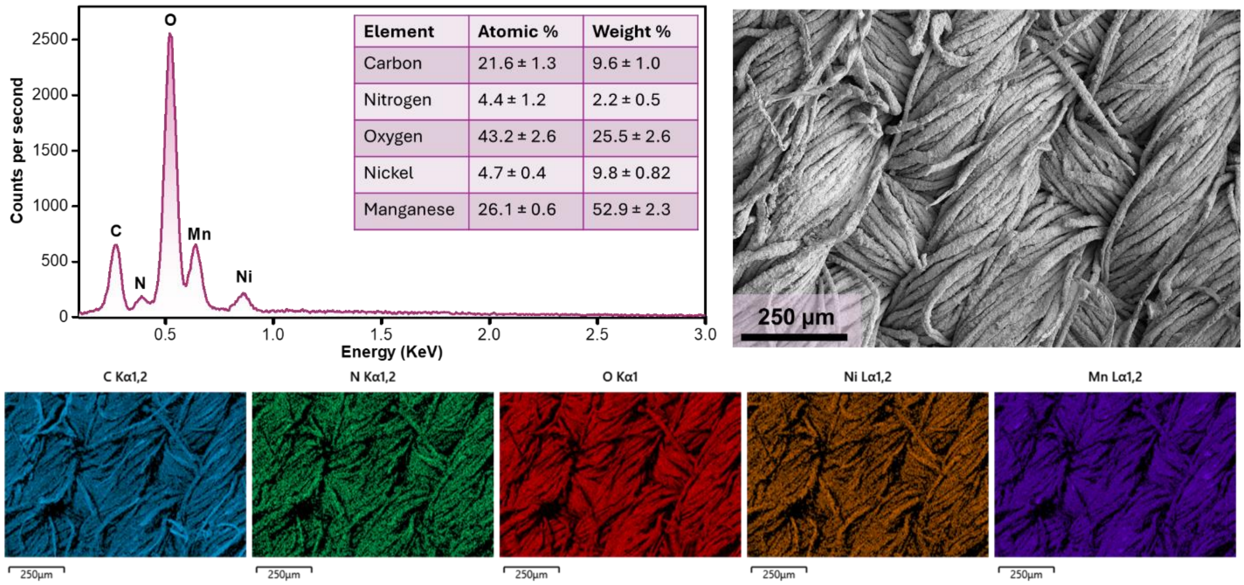

Figure S86. EDX spectrum and elemental mapping images of $Ni_3(HITP)_2$@textile after adsorption of 100 ppm of MnO_4^-.

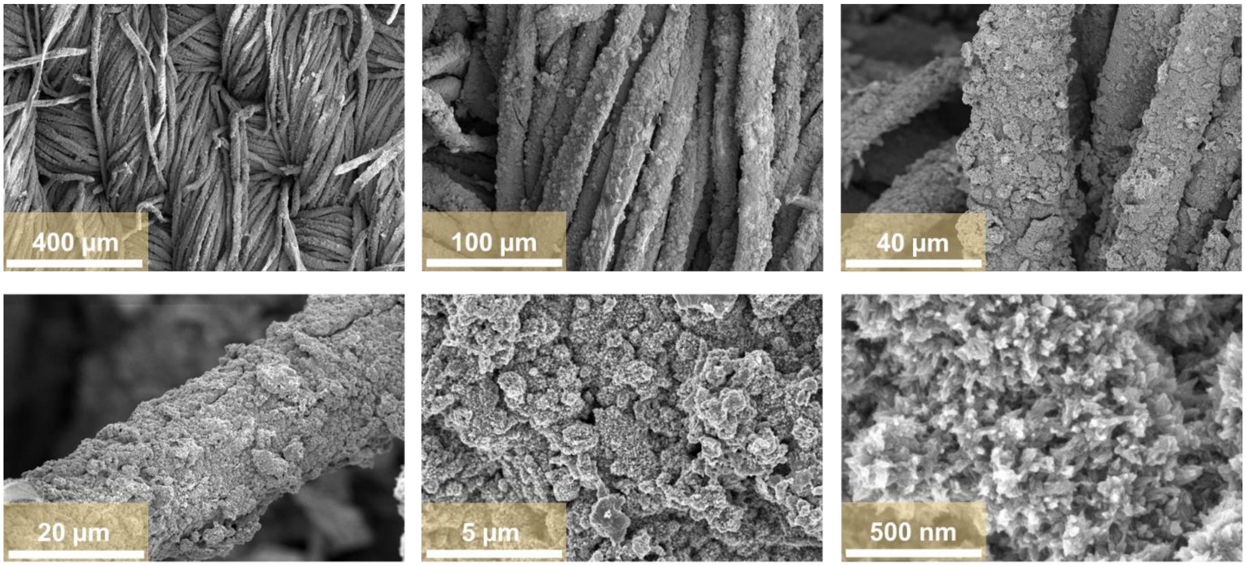

Figure S87. SEM micrographs of $Ni_3(HITP)_2$@textile at different magnifications following adsorption of 100 ppm of $Cr_2O_7^{2-}$.

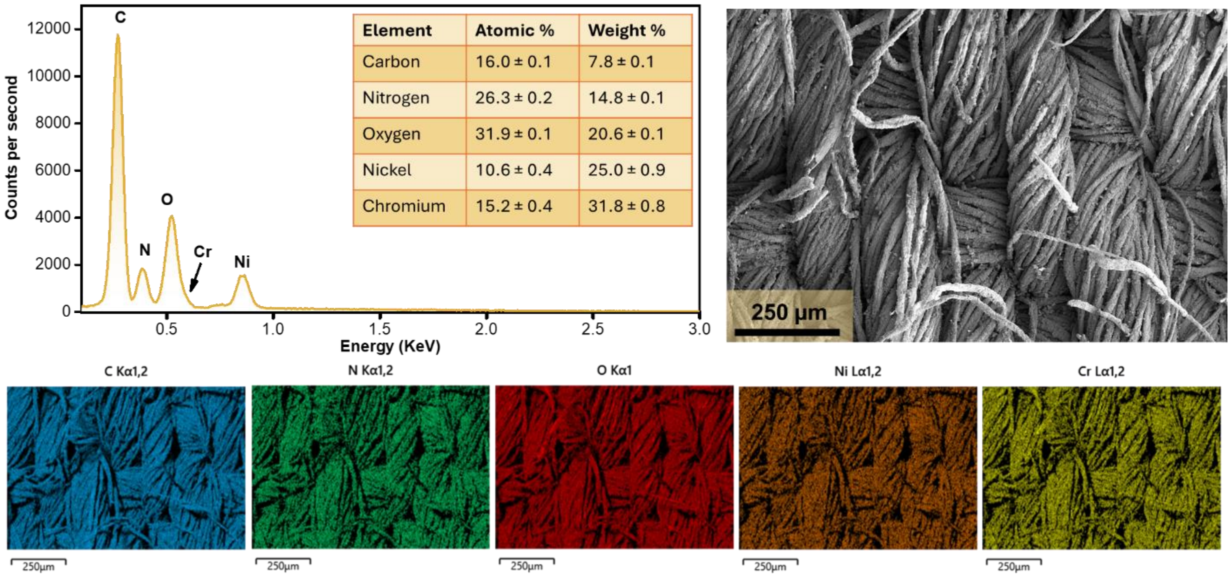

Figure S88. EDX spectrum and elemental mapping images of $Ni_3(HITP)_2$@textile after exposure to 100 ppm of $Cr_2O_7^{2-}$.

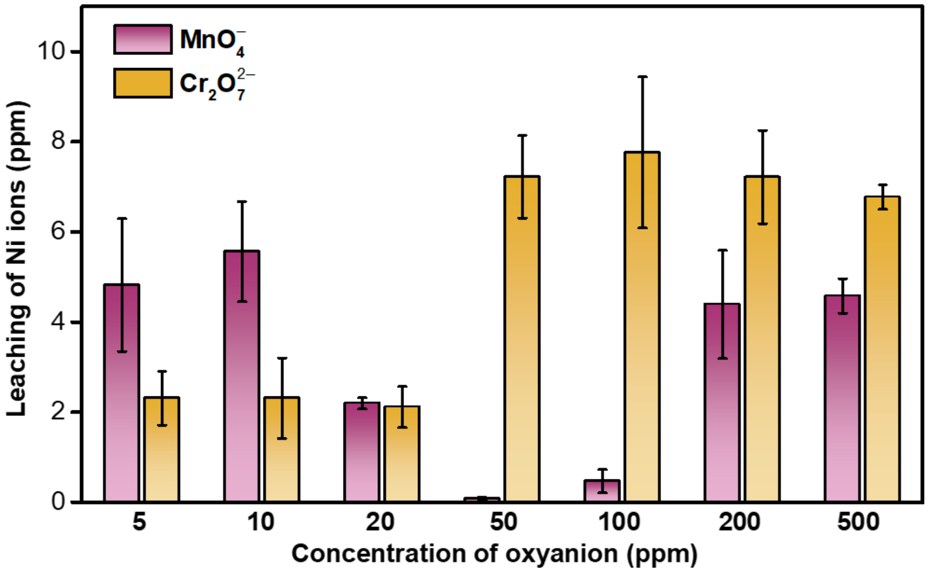

Figure S89. Leaching of nickel ions (in ppm) from bulk $Ni_3(HITP)_2$ after exposure to different concentrations of MnO_4^- and $Cr_2O_7^{2-}$ for 24 hours.

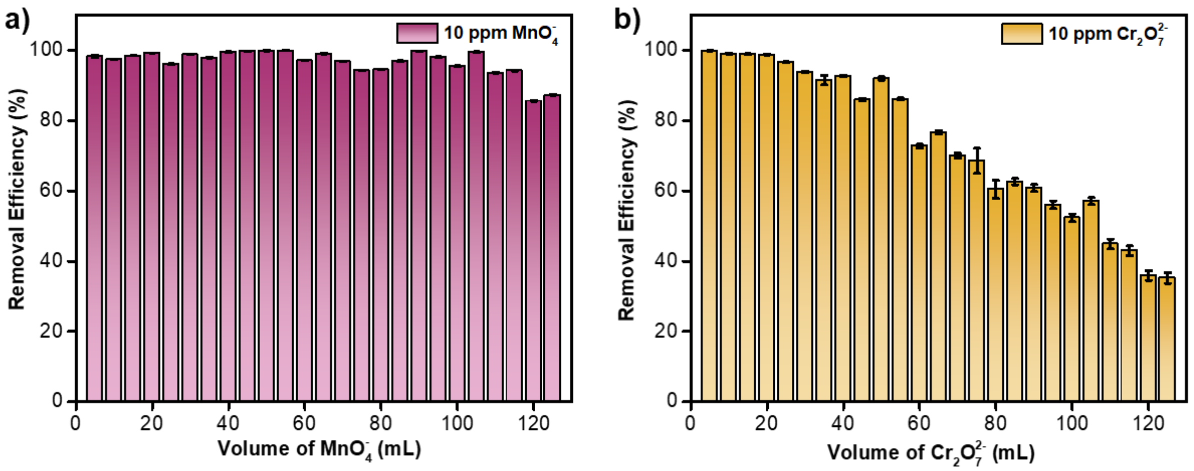

Figure S90. Continuous filtration performance of $Ni_3(HITP)_2$@textile composite (1 cm x 2 cm) upon the sequential addition of a) 5 mL of MnO_4^- (10 ppm) and b) 5 mL of $Cr_2O_7^{2-}$ (10 ppm).

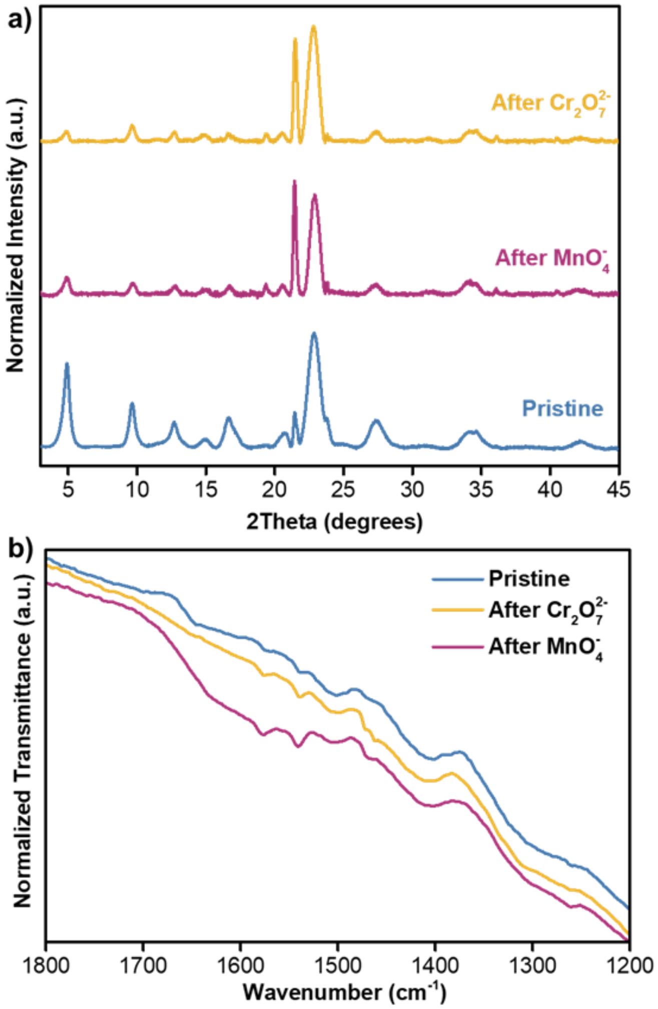

Figure S91. a) PXRD and b) ATR-IR measurements on $Ni_3(HITP)_2$@textiles following continuous adsorption of 10 ppm of MnO_4^- and $Cr_2O_7^{2-}$ (V = 125 mL).

8. Chemiresistive detection of oxyanions

8.1 Sensing setup

A line of liquid electrical tape coating was painted on each end of the $Ni_3(HITP)_2$@textile swatch (4.5 cm x 0.5 cm) approximately 3 mm from the edge, and allowed to dry. Carbon cloth was wrapped around each end of the textile and each wrapped end was connected to a Palmsens EmStat MUX8-R2 potentiostat (Palm Instruments, BV, Netherlands) with alligator clips. The textile was immersed in 10 mL DI water as shown below, and allowed to soak for at least 1 hr. After soaking, a driving voltage of 0.5 V was applied across the textile, and the textile equilibrated for at least 1 hour (or until the current response stabilized). Following equilibration, aliquots of stock solution were added to the solution while stirring at room temperature.

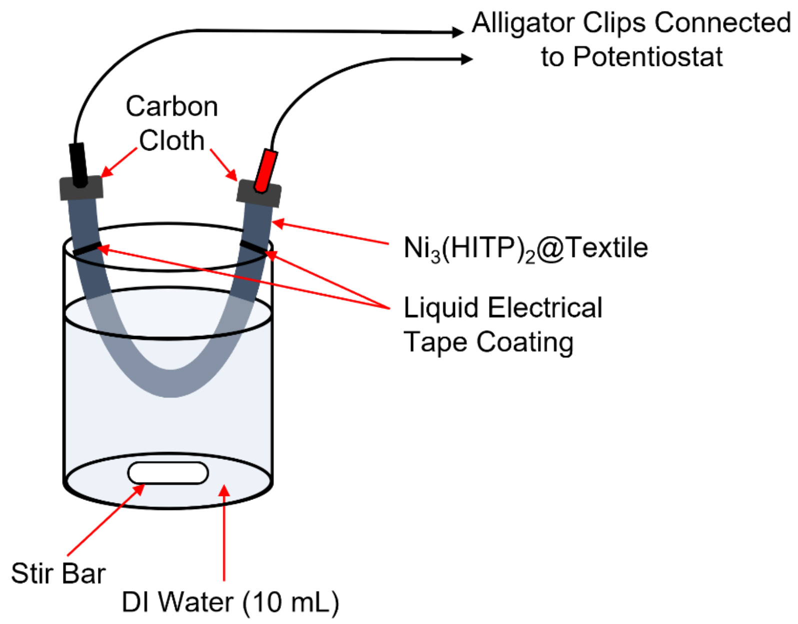

Figure S92. Illustration of the experimental setup for chemiresistive sensing of oxyanions using $Ni_3(HITP)_2$@textile.

8.2 Replicates of chemiresistive detection of MnO_4^-

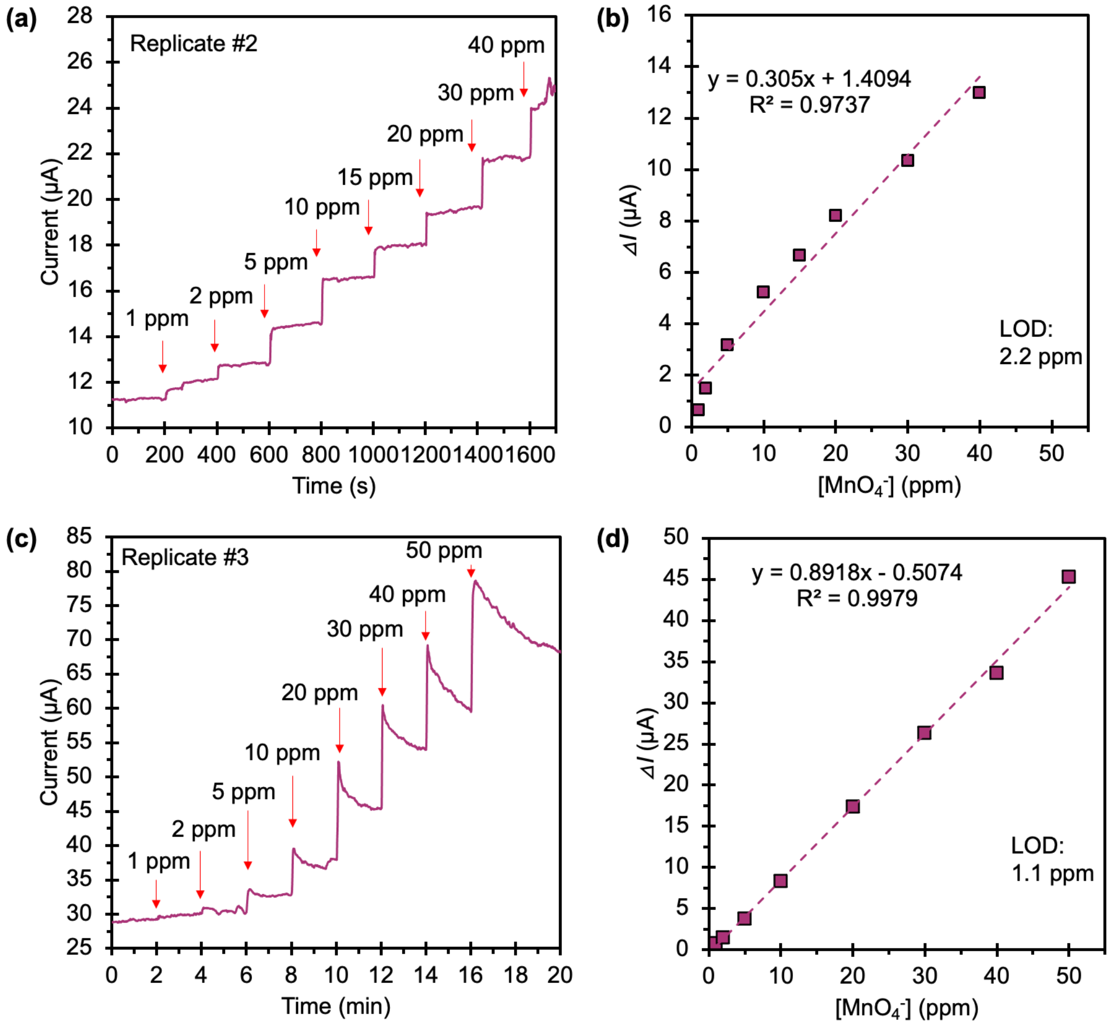

Figure S93. Additional replicates of chemiresistive detection of MnO_4^- with $Ni_3(HITP)_2$@textile swatches. (a,c) Current responses of textile swatches equilibrated in DI water at 0.5 V driving voltage to successive additions of MnO_4^-. (b,d) Response (change in current) vs. concentration for $Ni_3(HITP)_2$ textile swatches exposed to MnO_4^-. Calculated limits of detection (based on 3x S/N) are indicated on each plot.

8.3 Replicates of chemiresistive detection of $Cr_2O_7^{2-}$

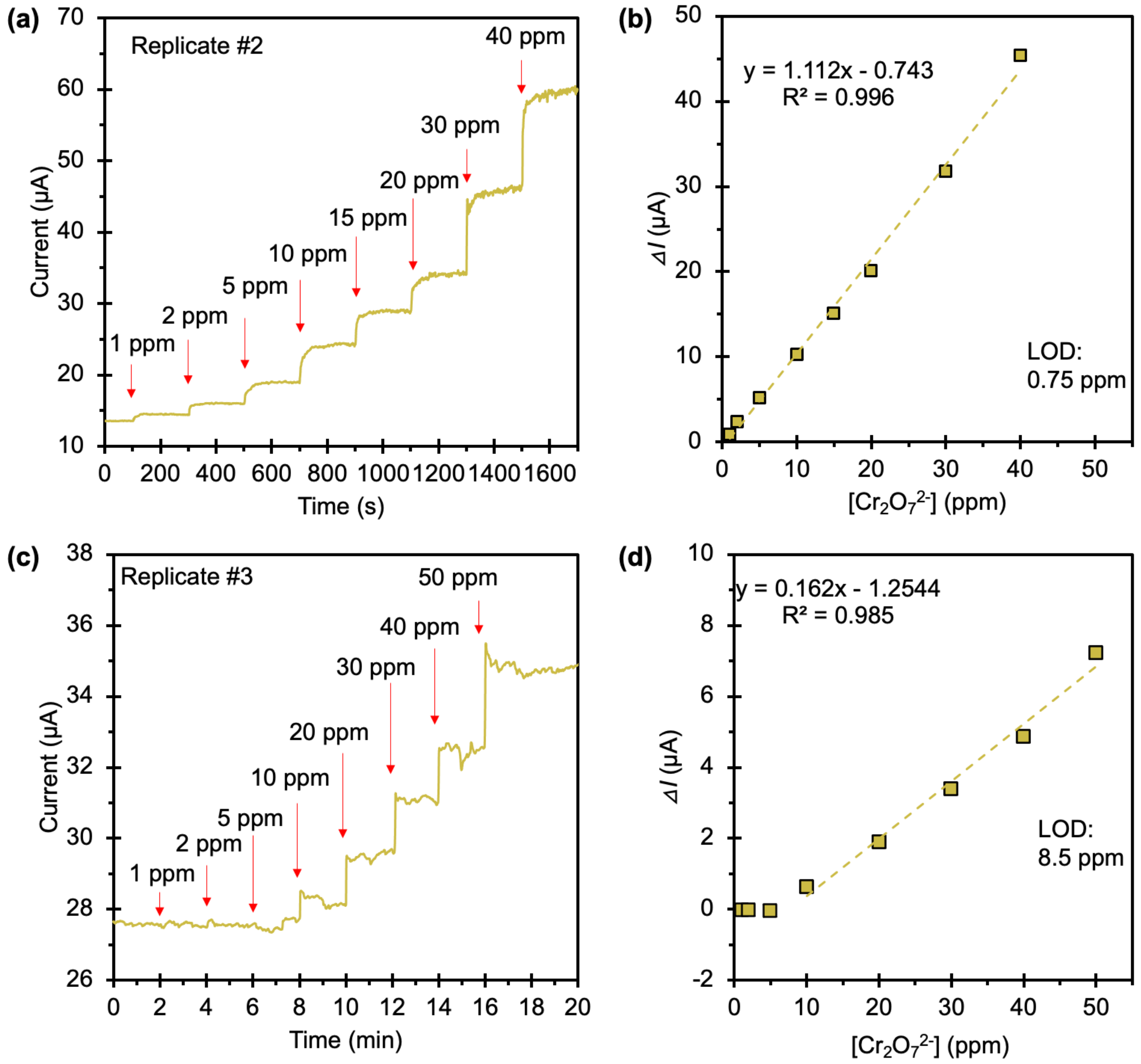

Figure S94. Additional replicates of chemiresistive detection of $Cr_2O_7^{2-}$ with $Ni_3(HITP)_2$@textile swatches. (a,c) Current responses of textile swatches equilibrated in DI water at 0.5 V driving voltage to successive additions of $Cr_2O_7^{2-}$. (b,d) Response (change in current) vs. concentration for $Ni_3(HITP)_2$ textile swatches exposed to $Cr_2O_7^{2-}$. Calculated limits of detection (based on 3x S/N) are indicated on each plot.

8.4 Calculation of the theoretical limit of detection (LoD)

8.4.1 Method 1

We calculated the theoretical limit of detection (LoD) for $Ni_3(HITP)_2$@textile swatches from these experiments using previously reported procedures.[52] First, we calculated the noise-based deviation in the current as the root mean squared (rms) value by fitting the first 121 data points of the baseline prior to oxyanion addition to a 5th order polynomial. We proceeded by calculating the sum of squared residuals (SSR) V_x^2 using **Equation S5** shown below, where y_i is measured current and y is the value calculated from the polynomial fit.

$$V_{x^2} = \sum(y_i - y)^2 \qquad \textbf{(Equation S5)}$$

We calculated the root-mean-square deviation (rms_{noise}) using **Equation S6**, where N is the number of data points of the baseline prior to oxyanion addition, which in all cases is 121.

$$rms_{noise} = \sqrt{\frac{V_{x^2}}{N}} \qquad \textbf{(Equation S6)}$$

Finally, the theoretical LoD was calculated from **Equation S7**, where *m* is the slope of the plots for concentration of oxyanion vs current change (μA) (**Figure 8b**).

$$LoD = 3.3\ x\ \frac{rms_{noise}}{m} \qquad \textbf{(Equation S7)}$$

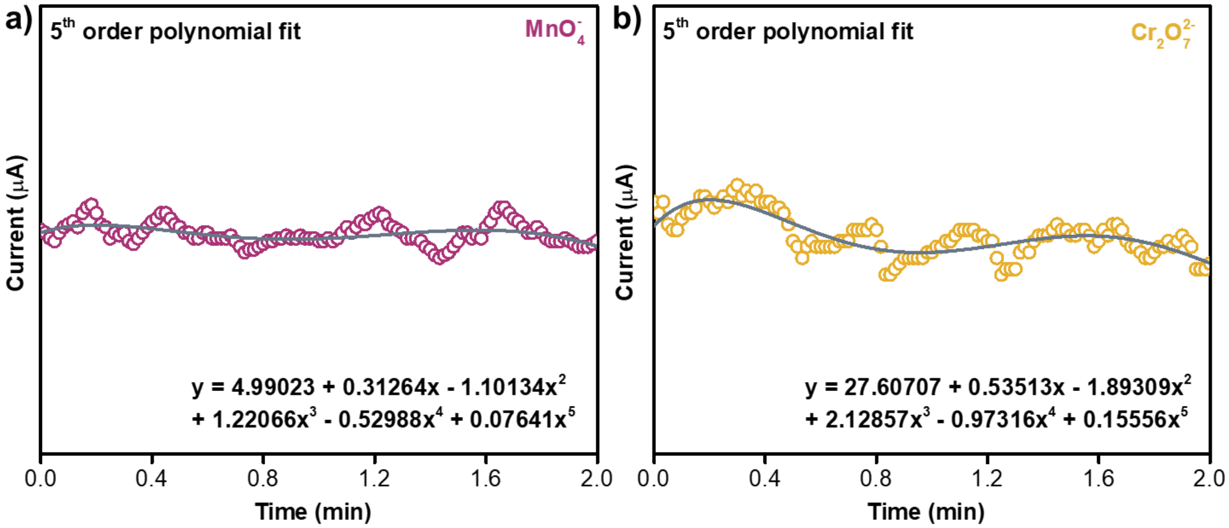

Figure S95. Representative 5th order polynomial fit for the first 121 baseline data points obtained for $Ni_3(HITP)_2$@textile swatches prior to the addition of a) MnO_4^- and b) $Cr_2O_7^{2-}$.

8.4.2 Method 2

The limit of detection (LOD) was determined from the signal-to-noise ratio (S/N)[53] as well as the slope and intercept of the linear straight line obtained from the concentration-dependent response plot (**Figure 8b**) according to **Equations S8 and S9**.

$$LOD = \frac{S/N - intercept}{slope} \qquad \textbf{(Equation S8)}$$

$$S/N = 3 \times \sigma_{baseline} \qquad \textbf{(Equation S9)}$$

where $\sigma_{baseline}$ is calculated as the standard deviation across the current values in the baseline (amperometric sensing experiment from 0 to 2 minutes).

Exemplary calculations:

For $KMnO_4$:

$$\sigma_{baseline} = 0.487$$

$$S/N = 3 \times 0.487 = 1.461$$

Using the equation of the linear fit in **Figure 8b**,

$$LOD = \frac{1.461 - 0.467}{0.299} = 3.31\, ppm$$

For $K_2Cr_2O_7$:

$\sigma_{baseline} = 0.0248$

$S/N = 3 \times\ 0.0248 = 0.0744$

Using the equation of the linear fit in **Figure 8b**,

$$LOD = \frac{0.0744 + 0.709}{0.132} = 5.93\, ppm$$

8.4.3 Comparison of LoDs

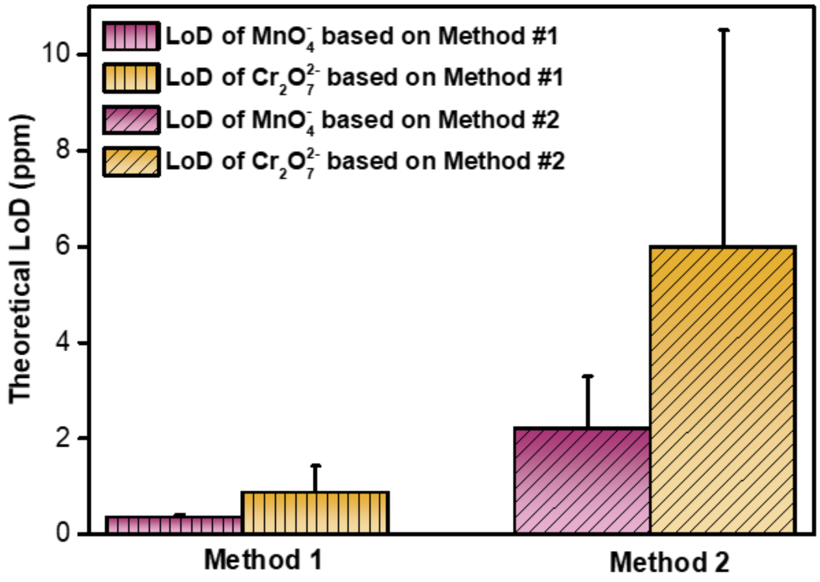

Figure S96. Comparison of the theoretical LoDs for a) MnO_4^- and b) $Cr_2O_7^{2-}$ determined using method #1 and method #2. To avoid overestimation, the LoDs obtained from method #2 are emphasized throughout the text.

8.5 Comparison of LoDs with literature

MOF material	Q_m (mg g^{-1})	LoD	Ref
MOR-2	193.7	4 ppb	14
ABT·2ClO$_4$	130	-	54
MONT-1	211.8	0.19 μM	22
Eu$_7$(mtb)$_5$(H$_2$O)$_{16}$]·NO$_3$·8DMA·18H$_2$O	9.7	0.56 ppb	55
NMOF-2	113.63	4 nM	56
BUT-39	215	1.5 μM	57
Nu-1000	76.8	1.8 μM	29
Th-BCTPE-1	1.05 *	4.6 nM	58
Hf-MOF-3	32	0.013 μM	59
Zr-MOF-3	30	0.019 μM	
Hf-MOF-2	153	0.188 μM	
Zr-MOF-2	149	0.244 μM	
Hf-MOF-1	27	0.138 μM	
Zr-MOF-1	28	0.138 μM	
[Cd(TIPA)$_2$(ClO$_4^-$)$_2$]·(DMF)$_3$(H$_2$O)	226.2	8 ppb	26
RhB@Zr-MOF	-	6.27 ppb	60
JLU-MOF60	149	0.38 μM	61
Dyes⊂MOF-801	83	0.03 mM	62
Ni$_3$(HITP)$_2$@textile	497	6 ppm	**This work**
SiO$_2$@NBDBIA **	363	25.2 ppb	63
CDs/zincone **	47.5	1 μM	64

Table S2. Comparison of Q_{max} and LoD of Ni$_3$(HITP)$_2$@textile towards $Cr_2O_7^{2-}$ with reported fluorescent MOFs used for dual sensing and capture. *Q_{max} value is reported in mol mol^{-1}. **Represents non-MOF materials.

MOF material	Q_e (mg g^{-1})	LoD	Ref
Eu-CMOF	1100	2.47 μM	[11]
Zn(II)-MOF	199.97	1.79 μM	[9]
$Ni_3(HITP)_2$@textile	827.3	2.2 ppm	**This work**
SiO_2@NBDBIA **	330	20.3 ppb	[63]
SiO_2@SFNO **	292	67 ppb	[65]

Table S3. Comparison of maximum adsorption capacity and detection limit of $Ni_3(HITP)_2$@textile towards MnO_4^- with reported fluorescent MOFs used for dual sensing and capture. **Represents non-MOF materials

8.6 Chemiresistive detection of oxyanions in the presence of an interference

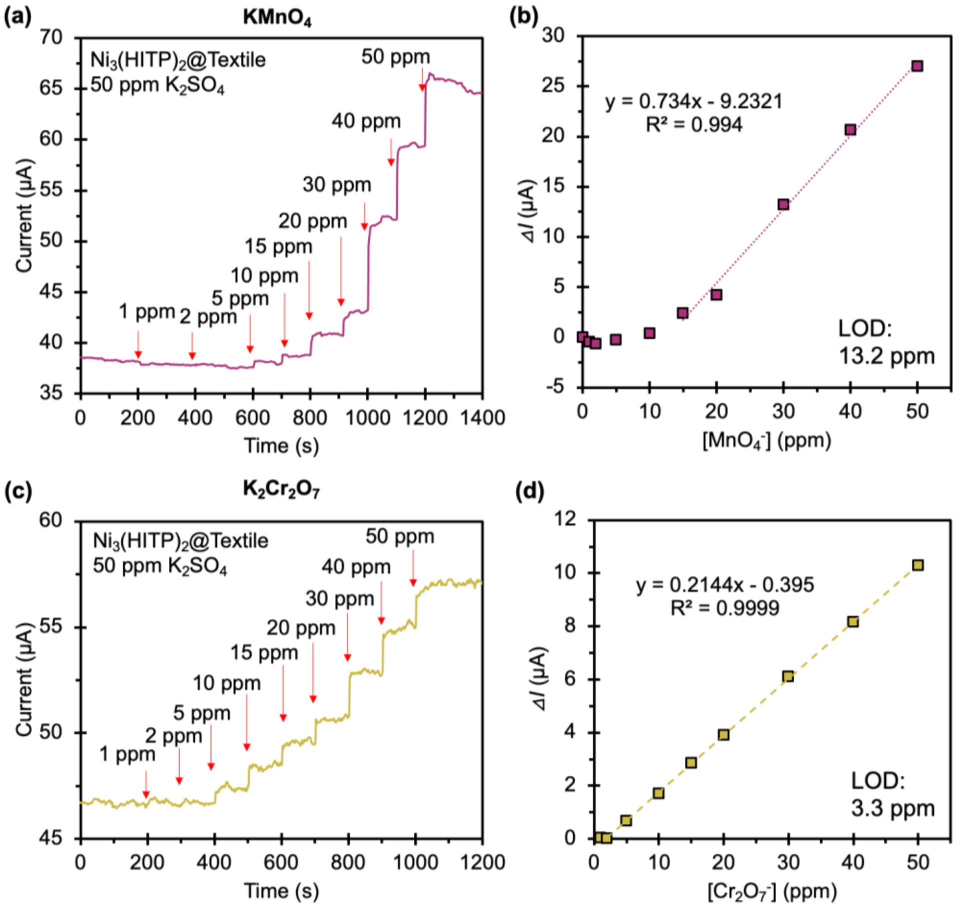

Figure S97. Chemiresistive detection of MnO_4^- and $Cr_2O_7^{2-}$ with $Ni_3(HITP)_2$ textile swatches in the presence of 50 ppm K_2SO_4. (a,c) Current responses of textile swatches equilibrated in DI water at 0.5 V driving voltage to successive additions of (a) MnO_4^- and (c) $Cr_2O_7^{2-}$. (b,d) Response (change in current) vs. concentration for $Ni_3(HITP)_2$ textile swatches exposed to (b) MnO_4^- and (d) $Cr_2O_7^{2-}$. Calculated limits of detection (based on 3x S/N) are indicated on each plot.

8.7 Control experiments

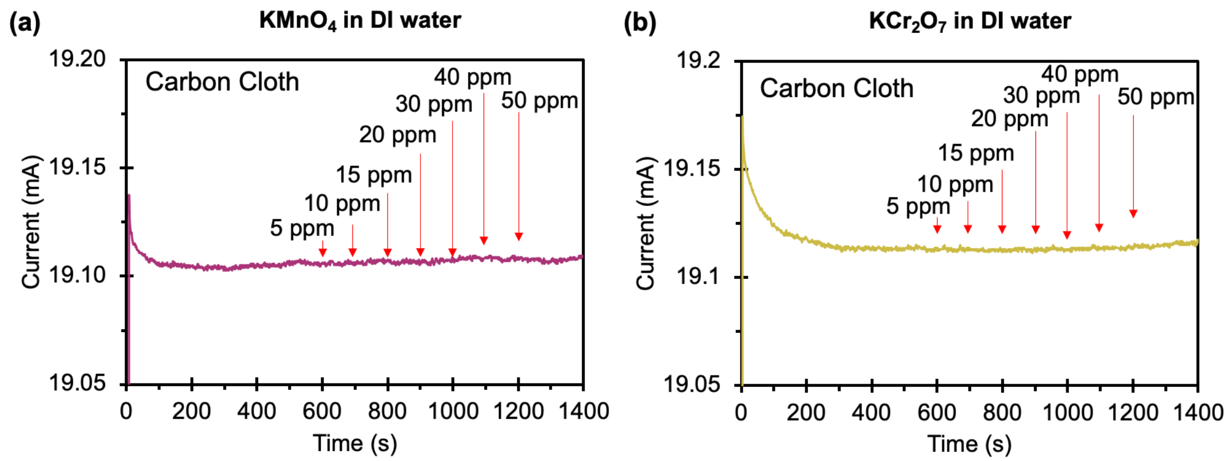

Figure S98. Current responses of carbon cloth swatches equilibrated in DI water at 0.05 V driving voltage to successive additions of (a) MnO_4^- and (b) $Cr_2O_7^{2-}$.

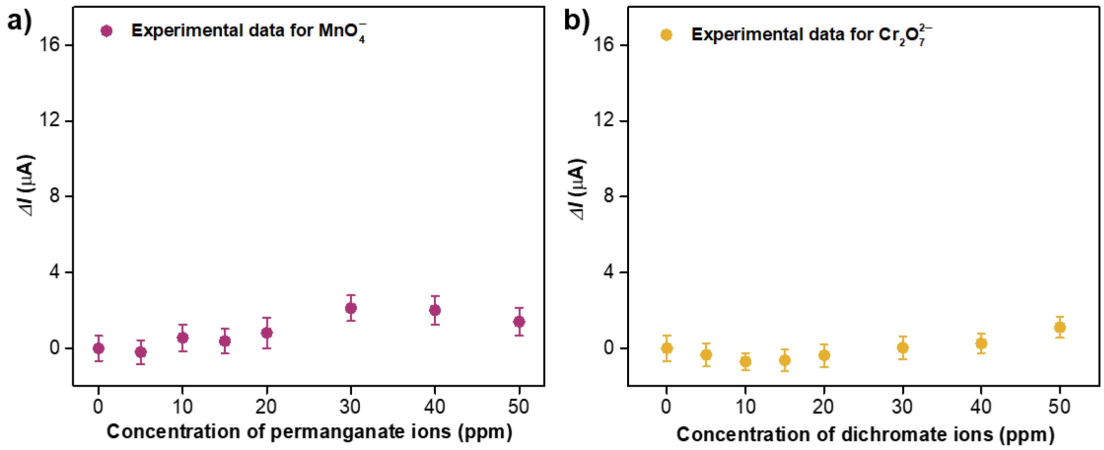

Figure S99. Correlation between response (change in current) and concentration for carbon cloth swatches exposed to different concentrations of a) MnO_4^- and b) $Cr_2O_7^{2-}$.

9. Impedance-Based Sensing with $Ni_3(HITP)_2$ films

A Gamry Instruments Interface 1010 Potentiostat was used for all electrochemical impedance measurements (Warminster, PA). Electrodes and electrochemical cells were purchased from CH Instruments (Austin, TX).

Preparation of $Ni_3(HITP)_2$ electrodes: 2 mg $Ni_3(HITP)_2$ powder were suspended in 1 mL DI water, and sonicated for 3 hrs until a homogeneous suspension was achieved. 2-2.5 uL drops of $Ni_3(HITP)_2$ suspension were successively dropcast onto the surface of a 3 mm dia. glassy carbon electrode (CH Instruments). Electrodes were air dried, and then used without further modification.

Electrochemical Sensing: All electrochemical experiments used Pt wire counter electrodes and Ag/AgCl electrodes filled with 1 M KCl. In a typical electrochemical experiment, 5 mL 0.1 M KCl was added to the electrochemical cell, electrodes were inserted and initial scans were run. Following initial scans, analyte was added to the desired concentration, and the system was equilibrated for 30 minutes, after which more scans were run. EIS experiments used the following parameters: Frequency range- 1 MHz–10 mHz, Amplitude- 10 mV, DC voltage- -0.3V, 0.0 V, or +0.3 V vs. Ag/AgCl.

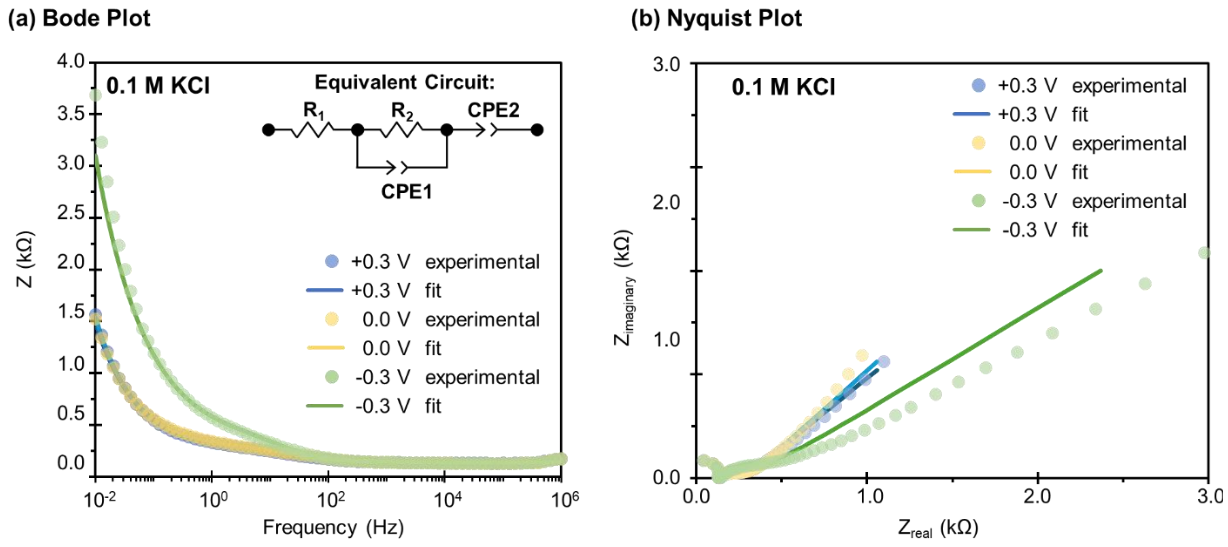

Figure S100. (a) Bode plot and (b) Nyquist plot for $Ni_3(HITP)_2$ electrode in 0.1 M KCl at -0.3V, 0.0V, and +0.3V vs. Ag/AgCl. Raw data (circles) was fitted to the equivalent circuit shown in the inset. Lines represent fitted data.

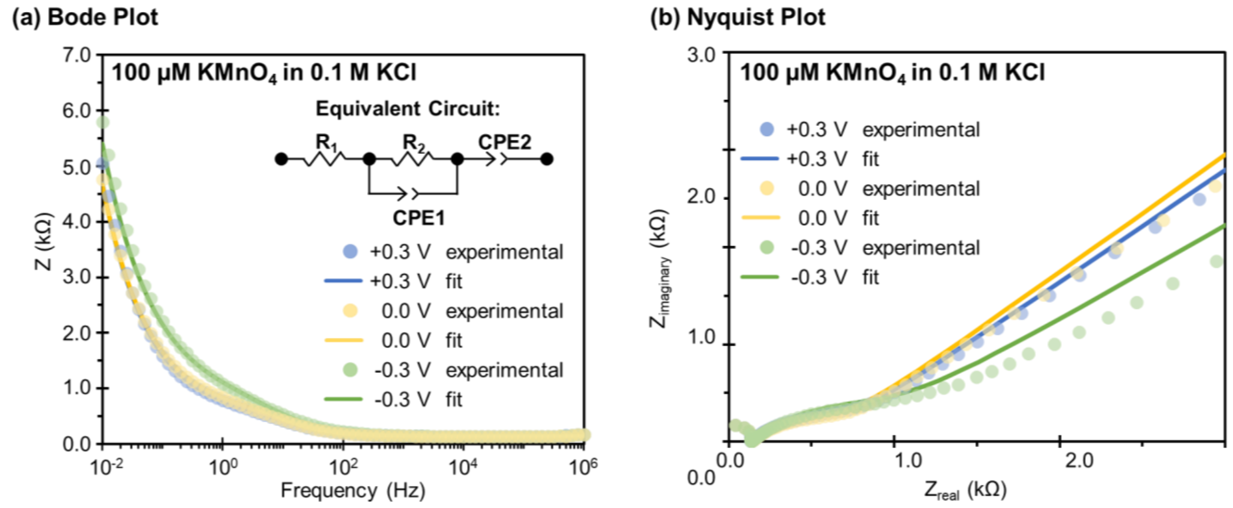

Figure S101. (a) Bode plot and (b) Nyquist plot for $Ni_3(HITP)_2$ electrode after 30 minutes in 100 µM $KMnO_4$ in 0.1 M KCl at -0.3V, 0.0V, and +0.3V vs. Ag/AgCl. Raw data (circles) was fitted to the equivalent circuit shown in the inset. Lines represent fitted data.

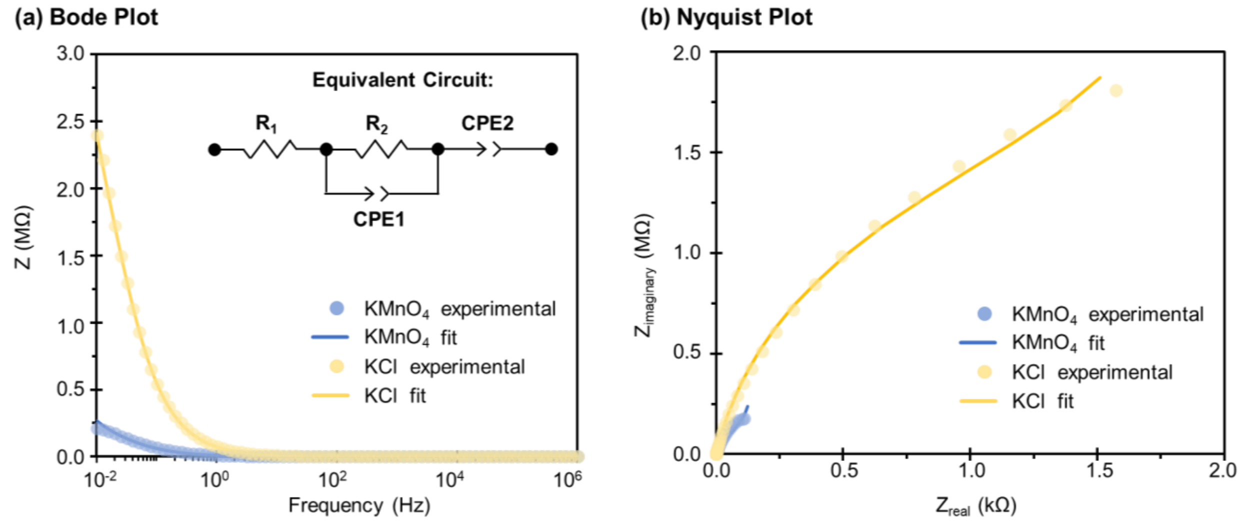

Figure S102. (a) Bode plot and (b) Nyquist plot for bare GCE electrode in 0.1 M KCl, and after 30 minutes in 100 µM $KMnO_4$ in 0.1 M KCl at 0.0V vs. Ag/AgCl. Raw data (circles) was fitted to the equivalent circuit shown in the inset. Lines represent fitted data.

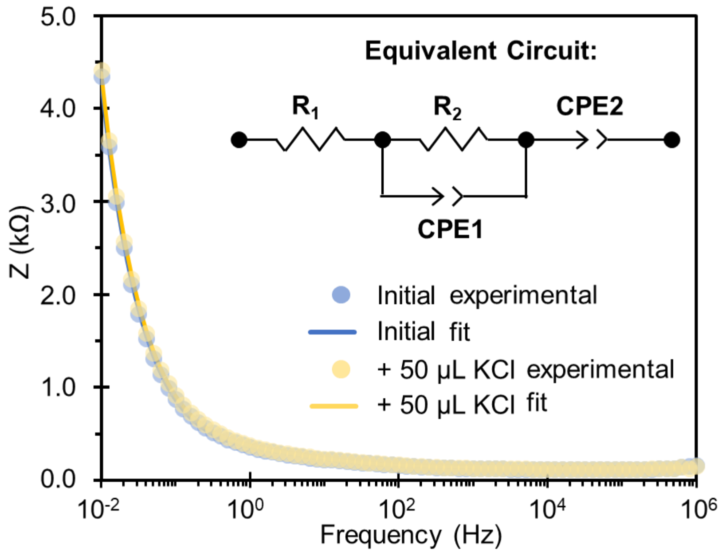

Figure S103. Bode plot for $Ni_3(HITP)_2$ electrode in 0.1 M KCl, and 30 minutes after an additional 50 µL 0.1 M KCl was added to the cell. DC voltage was 0.0V vs. Ag/AgCl. Raw data (circles) was fitted to the equivalent circuit shown in the inset. Lines represent fitted data.

10. References

1. Smith, M. K.; Mirica, K. A., Self-Organized Frameworks on Textiles (SOFT): Conductive Fabrics for Simultaneous Sensing, Capture, and Filtration of Gases. *J. Am. Chem. Soc.* **2017,** *139* (46), 16759-16767.

2. Stolz, R. M.; Kolln, A. F.; Rocha, B. C.; Brinks, A.; Eagleton, A. M.; Mendecki, L.; Vashisth, H.; Mirica, K. A., Epitaxial Self-Assembly of Interfaces of 2D Metal–Organic Frameworks for Electroanalytical Detection of Neurotransmitters. *ACS Nano* **2022,** *16* (9), 13869-13883.

3. Chen, T.; Dou, J.-H.; Yang, L.; Sun, C.; Libretto, N. J.; Skorupskii, G.; Miller, J. T.; Dincă, M., Continuous Electrical Conductivity Variation in M3(Hexaiminotriphenylene)2 (M = Co, Ni, Cu) MOF Alloys. *J. Am. Chem. Soc.* **2020,** *142* (28), 12367-12373.

4. Ambrogi, E. K.; Damacet, P.; Stolz, R. M.; Mirica, K. A., Mechanistic Insight into the Formation and Deposition of Conductive, Layered Metal–Organic Framework Nanocrystals. *ACS Nano* **2025,** *19* (1), 1383-1395.

5. Yang, L.; Dincă, M., Redox Ladder of Ni3 Complexes with Closed-Shell, Mono-, and Diradical Triphenylene Units: Molecular Models for Conductive 2D MOFs. *Angew. Chem. Int. Ed.* **2021,** *60* (44), 23784-23789.

6. Yang, L.; Oppenheim, J. J.; Dincă, M., Strong magnetic exchange coupling in a radical-bridged trinuclear nickel complex. *Dalton Trans.* **2022,** *51* (22), 8583-8587.

7. Debela, T. T.; Yang, M. C.; Hendon, C. H., Ligand-Mediated Hydrogenic Defects in Two-Dimensional Electrically Conductive Metal–Organic Frameworks. *J. Am. Chem. Soc.* **2023,** *145* (20), 11387-11391.

8. Fei, H.; Bresler, M. R.; Oliver, S. R. J., A New Paradigm for Anion Trapping in High Capacity and Selectivity: Crystal-to-Crystal Transformation of Cationic Materials. *J. Am. Chem. Soc.* **2011,** *133* (29), 11110-11113.

9. Kaur, H.; Walia, S.; Karmakar, A.; Krishnan, V.; Koner, R. R., Water-stable Zn-based metal-organic framework with hydrophilic-hydrophobic surface for selective adsorption and sensitive

detection of oxo-anions and pesticides in aqueous medium. *Journal of Environmental Chemical Engineering* **2022,** *10* (1), 106667.
10. Fajal, S.; Mandal, W.; Mollick, S.; More, Y. D.; Torris, A.; Saurabh, S.; Shirolkar, M. M.; Ghosh, S. K., Trap inlaid cationic hybrid composite material for efficient segregation of toxic chemicals from water. *Angew. Chem. Int. Ed.* **2022,** *61* (32), e202203385.
11. Li, X.; Zhang, S.; Zhang, L.; Yang, Y.; Zhang, K.; Cai, Y.; Xu, Y.; Gai, Y.; Xiong, K., Viologen-Based Cationic Metal–Organic Framework for Antibiotics Detection and MnO4– Removal in Water. *Cryst. Growth Des.* **2022,** *22* (7), 3991-3997.
12. Desai, A. V.; Sharma, S.; Roy, A.; Ghosh, S. K., Probing the role of anions in influencing the structure, stability, and properties in neutral N-Donor linker based metal–organic frameworks. *Cryst. Growth Des.* **2019,** *19* (12), 7046-7054.
13. Kaur, H.; Chandel, S. S.; Karmakar, A.; Sinha-Ray, S.; Krishnan, V.; Koner, R. R., Mercapto-decorated Zn-based metal-organic framework embedded nanofibrous membrane for oxo-anions treatment in aqueous solution. *Chem. Eng. J.* **2022,** *443*, 136212.
14. Rapti, S.; Sarma, D.; Diamantis, S. A.; Skliri, E.; Armatas, G. S.; Tsipis, A. C.; Hassan, Y. S.; Alkordi, M.; Malliakas, C. D.; Kanatzidis, M. G.; Lazarides, T.; Plakatouras, J. C.; Manos, M. J., All in one porous material: exceptional sorption and selective sensing of hexavalent chromium by using a Zr4+ MOF. *J. Mater. Chem. A.* **2017,** *5* (28), 14707-14719.
15. Fu, H.-R.; Xu, Z.-X.; Zhang, J., Water-Stable Metal–Organic Frameworks for Fast and High Dichromate Trapping via Single-Crystal-to-Single-Crystal Ion Exchange. *Chem. Mater.* **2015,** *27* (1), 205-210.
16. Zhang, Q.; Yu, J.; Cai, J.; Zhang, L.; Cui, Y.; Yang, Y.; Chen, B.; Qian, G., A porous Zr-cluster-based cationic metal–organic framework for highly efficient Cr2O72− removal from water. *Chem. Commun.* **2015,** *51* (79), 14732-14734.
17. Desai, A. V.; Manna, B.; Karmakar, A.; Sahu, A.; Ghosh, S. K., A water-stable cationic metal–organic framework as a dual adsorbent of oxoanion pollutants. *Angew. Chem.* **2016,** *128* (27), 7942-7946.
18. Rapti, S.; Pournara, A.; Sarma, D.; Papadas, I. T.; Armatas, G. S.; Hassan, Y. S.; Alkordi, M. H.; Kanatzidis, M. G.; Manos, M. J., Rapid, green and inexpensive synthesis of high quality UiO-66 amino-functionalized materials with exceptional capability for removal of hexavalent chromium from industrial waste. *Inorganic Chemistry Frontiers* **2016,** *3* (5), 635-644.
19. Li, L.-L.; Feng, X.-Q.; Han, R.-P.; Zang, S.-Q.; Yang, G., Cr(VI) removal via anion exchange on a silver-triazolate MOF. *J. Hazard. Mater.* **2017,** *321*, 622-628.
20. Zheng, T.-R.; Qian, L.-L.; Li, M.; Wang, Z.-X.; Li, K.; Zhang, Y.-Q.; Li, B.-L.; Wu, B., A bifunctional cationic metal–organic framework based on unprecedented nonanuclear copper(ii) cluster for high dichromate and chromate trapping and highly efficient photocatalytic degradation of organic dyes under visible light irradiation. *Dalton Trans.* **2018,** *47* (27), 9103-9113.
21. Li, C.-P.; Zhou, H.; Wang, S.; Chen, J.; Wang, Z.-L.; Du, M., Highly efficient Cr2O72− removal of a 3D metal-organic framework fabricated by tandem single-crystal to single-crystal transformations from a 1D coordination array. *Chem. Commun.* **2017,** *53* (66), 9206-9209.
22. Ding, B.; Guo, C.; Liu, S. X.; Cheng, Y.; Wu, X. X.; Su, X. M.; Liu, Y. Y.; Li, Y., A unique multi-functional cationic luminescent metal–organic nanotube for highly sensitive detection of dichromate and selective high capacity adsorption of Congo red. *RSC Adv.* **2016,** *6* (40), 33888-33900.
23. Zhu, K.; Chen, C.; Xu, H.; Gao, Y.; Tan, X.; Alsaedi, A.; Hayat, T., Cr(VI) Reduction and Immobilization by Core-Double-Shell Structured Magnetic Polydopamine@Zeolitic Idazolate Frameworks-8 Microspheres. *ACS Sustain. Chem. Eng.* **2017,** *5* (8), 6795-6802.

24. Nasrollahpour, A.; Moradi, S. E., Hexavalent chromium removal from water by ionic liquid modified metal-organic frameworks adsorbent. *Microporous Mesoporous Mater.* **2017,** *243*, 47-55.
25. Lv, X.-X.; Shi, L.-L.; Li, K.; Li, B.-L.; Li, H.-Y., An unusual porous cationic metal–organic framework based on a tetranuclear hydroxyl-copper (II) cluster for fast and highly efficient dichromate trapping through a single-crystal to single-crystal process. *Chem. Commun.* **2017,** *53* (11), 1860-1863.
26. Fu, H.-R.; Zhao, Y.; Zhou, Z.; Yang, X.-G.; Ma, L.-F., Neutral ligand TIPA-based two 2D metal–organic frameworks: ultrahigh selectivity of C2H2/CH4 and efficient sensing and sorption of Cr(vi). *Dalton Trans.* **2018,** *47* (11), 3725-3732.
27. Yang, X.; Yan, C.; Li, Z.; Li, X.; Yu, Q.; Sang, T.; Gai, Y.; Zhang, Q.; Xiong, K., Viologen-Based Cationic Metal–Organic Framework for Efficient Cr2O72– Adsorption and Dye Separation. *Inorg. Chem.* **2021,** *60* (8), 5988-5995.
28. Zhang, C.; Liu, Y.; Sun, L.; Shi, H.; Shi, C.; Liang, Z.; Li, J., A Zwitterionic Ligand-Based Cationic Metal-Organic Framework for Rapidly Selective Dye Capture and Highly Efficient Cr2O72− Removal. *Chem. Eur. J* **2018,** *24* (11), 2718-2724.
29. Lin, Z.-J.; Zheng, H.-Q.; Zheng, H.-Y.; Lin, L.-P.; Xin, Q.; Cao, R., Efficient Capture and Effective Sensing of Cr2O72– from Water Using a Zirconium Metal–Organic Framework. *Inorg. Chem.* **2017,** *56* (22), 14178-14188.
30. Liu, J.; Ye, Y.; Sun, X.; Liu, B.; Li, G.; Liang, Z.; Liu, Y., A multifunctional Zr (iv)-based metal–organic framework for highly efficient elimination of Cr (vi) from the aqueous phase. *J. Mater. Chem. A.* **2019,** *7* (28), 16833-16841.
31. Xue, H.; Huang, X.-S.; Yin, Q.; Hu, X.-J.; Zheng, H.-Q.; Huang, G.; Liu, T.-F., Bimetallic cationic metal–organic frameworks for selective dye adsorption and effective Cr2O72–removal. *Cryst. Growth Des.* **2020,** *20* (8), 4861-4866.
32. Yuan, S.; Qin, J.-S.; Zou, L.; Chen, Y.-P.; Wang, X.; Zhang, Q.; Zhou, H.-C., Thermodynamically Guided Synthesis of Mixed-Linker Zr-MOFs with Enhanced Tunability. *J. Am. Chem. Soc.* **2016,** *138* (20), 6636-6642.
33. Zhang, X.; Fan, W.; Fu, M.; Jiang, W.; Lu, K.; Wang, Y.; Sun, D., Optimizing zirconium metal–organic frameworks through steric tuning for efficient removal of Cr2O72−. *Chem. Commun.* **2020,** *56* (72), 10513-10516.
34. Yang, Z.-h.; Cao, J.; Chen, Y.-p.; Li, X.; Xiong, W.-p.; Zhou, Y.-y.; Zhou, C.-y.; Xu, R.; Zhang, Y.-r., Mn-doped zirconium metal-organic framework as an effective adsorbent for removal of tetracycline and Cr(VI) from aqueous solution. *Microporous Mesoporous Mater.* **2019,** *277*, 277-285.
35. He, T.; Zhang, Y.-Z.; Kong, X.-J.; Yu, J.; Lv, X.-L.; Wu, Y.; Guo, Z.-J.; Li, J.-R., Zr (IV)-based metal-organic framework with T-shaped ligand: unique structure, high stability, selective detection, and rapid adsorption of Cr2O72–in water. *ACS Appl. Mater. Interfaces.* **2018,** *10* (19), 16650-16659.
36. Guo, X.; Wang, J., A general kinetic model for adsorption: Theoretical analysis and modeling. *J. Mol. Liq.* **2019,** *288*, 111100.
37. Giannozzi, P.; Andreussi, O.; Brumme, T.; Bunau, O.; Nardelli, M. B.; Calandra, M.; Car, R.; Cavazzoni, C.; Ceresoli, D.; Cococcioni, M., Advanced capabilities for materials modelling with Quantum ESPRESSO. *J. Phys.: Condens. Matter* **2017,** *29* (46), 465901.
38. Perdew, J. P.; Burke, K.; Ernzerhof, M., Generalized gradient approximation made simple. *Phys. Rev. Lett.* **1996,** *77* (18), 3865.
39. Grimme, S.; Antony, J.; Ehrlich, S.; Krieg, H., A consistent and accurate ab initio parametrization of density functional dispersion correction (DFT-D) for the 94 elements H-Pu. *J. Chem. Phys.* **2010,** *132* (15).

40. Sheberla, D.; Sun, L.; Blood-Forsythe, M. A.; Er, S.; Wade, C. R.; Brozek, C. K.; Aspuru-Guzik, A.; Dincă, M., High Electrical Conductivity in Ni3(2,3,6,7,10,11-hexaiminotriphenylene)2, a Semiconducting Metal–Organic Graphene Analogue. *J. Am. Chem. Soc.* **2014,** *136* (25), 8859-8862.
41. Eagleton, A. M.; Ambrogi, E. K.; Miller, S. A.; Vereshchuk, N.; Mirica, K. A., Fiber Integrated Metal-Organic Frameworks as Functional Components in Smart Textiles. *Angew. Chem. Int. Ed.* **2023,** *62* (49), e202309078.
42. Jiao, C.; Liu, D.; Chen, X.; Chen, J.; Ye, D., Durable, multifunctional cotton fabrics with in situ deposited micro/nanomaterials for effective self–cleaning, oil–water separation and antibacterial activity. *Int. J. Biol. Macromol.* **2024,** *269*, 131848.
43. Tang, P.; Jiang, Q.; Chen, W.; Wu, R.; Han, J.; Zhang, C., Innovative design of superhydrophobic and antimicrobial cotton fabrics: A multistage roughness structure approach with dual ZIF particles for sustainable salt-free dyeing. *Industrial Crops and Products* **2024,** *221*, 119342.
44. Ma, K.; Idrees, K. B.; Son, F. A.; Maldonado, R.; Wasson, M. C.; Zhang, X.; Wang, X.; Shehayeb, E.; Merhi, A.; Kaafarani, B. R.; Islamoglu, T.; Xin, J. H.; Farha, O. K., Fiber Composites of Metal–Organic Frameworks. *Chem. Mater.* **2020,** *32* (17), 7120-7140.
45. Ma, K.; Islamoglu, T.; Chen, Z.; Li, P.; Wasson, M. C.; Chen, Y.; Wang, Y.; Peterson, G. W.; Xin, J. H.; Farha, O. K., Scalable and Template-Free Aqueous Synthesis of Zirconium-Based Metal–Organic Framework Coating on Textile Fiber. *J. Am. Chem. Soc.* **2019,** *141* (39), 15626-15633.
46. Li, W.; Zhang, Y.; Yu, Z.; Zhu, T.; Kang, J.; Liu, K.; Li, Z.; Tan, S. C., In Situ Growth of a Stable Metal–Organic Framework (MOF) on Flexible Fabric via a Layer-by-Layer Strategy for Versatile Applications. *ACS Nano* **2022,** *16* (9), 14779-14791.
47. Qin, H.; Lv, Y.; Nakane, K., In situ growth of Bi-MOF on cotton fabrics via ultrasonic synthesis strategy for recyclable photocatalytic textiles. *RSC Adv.* **2024,** *14* (16), 11513-11523.
48. Yu, B.; Wang, P.; Wang, L.; Yang, F.; Zhang, G.; Cai, Z.; Chen, M.; Zhu, X.; Fu, B., In situ growth of ZIF-67 on silanization-modified cellulose fibers with enhanced toluene adsorption property and hydrophobicity. *Sep. Purif. Technol.* **2025,** *360*, 131222.
49. Kim, M.-K.; Kim, S. H.; Park, M.; Ryu, S. G.; Jung, H., Degradation of chemical warfare agents over cotton fabric functionalized with UiO-66-NH2. *RSC Adv.* **2018,** *8* (72), 41633-41638.
50. Damacet, P.; Shehayeb, E. O.; Mirica, K. A., Controlling the Spatiotemporal Self-Organization of Stimuli-Responsive Nanocrystals under Out-of-Equilibrium Conditions. *J. Am. Chem. Soc.* **2025,** *147* (2), 1584-1594.
51. Behboudikhiavi, S.; Chanteux, G.; Babu, B.; Faniel, S.; Marlec, F.; Robert, K.; Magnin, D.; Lucaccioni, F.; Omale, J. O.; Apostol, P., Direct Electrodeposition of Electrically Conducting Ni3 (HITP) 2 MOF Nanostructures for Micro-Supercapacitor Integration. *Small* **2024,** *20* (36), 2401509.
52. Ammu, S.; Dua, V.; Agnihotra, S. R.; Surwade, S. P.; Phulgirkar, A.; Patel, S.; Manohar, S. K., Flexible, All-Organic Chemiresistor for Detecting Chemically Aggressive Vapors. *J. Am. Chem. Soc.* **2012,** *134* (10), 4553-4556.
53. Li, H.; Wu, Y.; Xu, Z.; Wang, Y., Controllable preparation of a Cu NCs@ Zn-MOF hybrid with dual emission induced by an ion exchange strategy for the detection of explosives. *ACS Sens.* **2024,** *9* (9), 4701-4710.
54. Li, X.; Xu, H.; Kong, F.; Wang, R., A cationic metal–organic framework consisting of nanoscale cages: capture, separation, and luminescent probing of Cr2O72− through a single-crystal to single-crystal process. *Angew. Chem. Int. Ed.* **2013,** *52* (51), 13769-13773.
55. Liu, W.; Wang, Y.; Bai, Z.; Li, Y.; Wang, Y.; Chen, L.; Xu, L.; Diwu, J.; Chai, Z.; Wang, S., Hydrolytically Stable Luminescent Cationic Metal Organic Framework for Highly Sensitive and Selective Sensing of Chromate Anions in Natural Water Systems. *ACS Appl. Mater. Interfaces.* **2017,** *9* (19), 16448-16457.

56. Mukherjee, S.; Ganguly, S.; Samanta, D.; Das, D., Sustainable Green Route to Synthesize Functional Nano-MOFs as Selective Sensing Probes for CrVI Oxoanions and as Specific Sequestering Agents for Cr2O72–. *ACS Sustain. Chem. Eng.* **2020,** *8* (2), 1195-1206.
57. He, T.; Zhang, Y.-Z.; Kong, X.-J.; Yu, J.; Lv, X.-L.; Wu, Y.; Guo, Z.-J.; Li, J.-R., Zr(IV)-Based Metal-Organic Framework with T-Shaped Ligand: Unique Structure, High Stability, Selective Detection, and Rapid Adsorption of Cr2O72– in Water. *ACS Appl. Mater. Interfaces.* **2018,** *10* (19), 16650-16659.
58. Li, Z.-J.; Ju, Y.; Wu, X.-L.; Li, X.; Qiu, J.; Li, Y.; Zhang, Z.-H.; He, M.-Y.; Zhang, L.; Wang, J.-Q.; Lin, J., Topological control of metal–organic frameworks toward highly sensitive and selective detection of chromate and dichromate. *Inorganic Chemistry Frontiers* **2023,** *10* (6), 1721-1730.
59. Wu, K.; Zheng, J.; Huang, Y.-L.; Luo, D.; Li, Y. Y.; Lu, W.; Li, D., Cr2O72− inside Zr/Hf-based metal–organic frameworks: highly sensitive and selective detection and crystallographic evidence. *Journal of Materials Chemistry C* **2020,** *8* (47), 16974-16983.
60. Li, Q.; Li, D.; Wu, Z.-Q.; Shi, K.; Liu, T.-H.; Yin, H.-Y.; Cai, X.-B.; Fan, Z.-L.; Zhu, W.; Xue, D.-X., RhB-Embedded Zirconium–Biquinoline-Based MOF Composite for Highly Sensitive Probing Cr(VI) and Photochemical Removal of CrO42–, Cr2O72–, and MO. *Inorg. Chem.* **2022,** *61* (38), 15213-15224.
61. Liu, J.; Ye, Y.; Sun, X.; Liu, B.; Li, G.; Liang, Z.; Liu, Y., A multifunctional Zr(iv)-based metal–organic framework for highly efficient elimination of Cr(vi) from the aqueous phase. *J. Mater. Chem. A.* **2019,** *7* (28), 16833-16841.
62. Yoo, J.; Ryu, U.; Kwon, W.; Choi, K. M., A multi-dye containing MOF for the ratiometric detection and simultaneous removal of Cr2O72− in the presence of interfering ions. *Sensor Actuat B: Chem* **2019,** *283*, 426-433.
63. Yadav, S.; Choudhary, N.; Bhai, S.; Bhojani, G.; Chatterjee, S.; Ganguly, B.; Paital, A. R., Recyclable Functionalized Material for Sensitive Detection and Exceptional Sorption of Hexavalent Chromium and Permanganate Ions with Biosensing Applications. *ACS Appl. Bio Mater.* **2021,** *4* (8), 6430-6440.
64. Xue, D.; Yu, F.; Zhang, Z.; Yang, Y., One-step synthesis of carbon dots embedded zincone microspheres for luminescent detection and removal of dichromate anions in water. *Sensor Actuat B: Chem* **2019,** *279*, 130-137.
65. Chatterjee, S.; Qin, J.; Li, X.; Liang, F.; Rai, D. K.; Yang, Y.-W., Safranin O-functionalized cuboid mesoporous silica material for fluorescent sensing and adsorption of permanganate. *J. Mater. Chem. B.* **2020,** *8* (11), 2238-2249.
